# Coiling directions in the planktonic foraminifer *Pulleniatina*: A complex eco-evolutionary dynamic spanning millions of years

**DOI:** 10.1371/journal.pone.0249113

**Published:** 2021-04-13

**Authors:** Paul N. Pearson, Luke Penny

**Affiliations:** School of Earth and Ocean Sciences, Cardiff University, Cardiff, United Kingdom; Baylor University, UNITED STATES

## Abstract

Planktonic foraminifera are heterotrophic sexually reproducing marine protists with an exceptionally complete fossil record that provides unique insights into long-term patterns and processes of evolution. Populations often exhibit strong biases towards either right (dextral) or left (sinistral) shells. Deep-sea sediment cores spanning millions of years reveal that some species show large and often rapid fluctuations in their dominant coiling direction through time. This is useful for biostratigraphic correlation but further work is required to understand the population dynamical processes that drive these fluctuations. Here we address the case of coiling fluctuations in the planktonic foraminifer genus *Pulleniatina* based on new high-resolution counts from two recently recovered sediment cores from either side of the Indonesian through-flow in the tropical west Pacific and Indian Oceans (International Ocean Discovery Program Sites U1486 and U1483). We use single-specimen stable isotope analyses to show that dextral and sinistral shells from the same sediment samples can show significant differences in both carbon and oxygen isotopes, implying a degree of ecological separation between populations. In one case we detect a significant difference in size between dextral and sinistral specimens. We suggest that major fluctuations in coiling ratio are caused by cryptic populations replacing one another in competitive sweeps, a mode of evolution that is more often associated with asexual organisms than with the classical ‘biological species concept’.

## Introduction

Populations of most species of planktonic foraminifera are distributed globally at very high abundance within broad climatic zones, forming a major part of open marine ecosystems [1]. They are carried passively by the ocean current systems, including through the gateways that link the major ocean basins. Each organism secretes a shell of calcium carbonate composed of inter-connected chambers. Most species add their chambers in a trochospiral (expanding helical) arrangement, thereby defining a coiling direction. The flux of dead shells to the sea floor is a significant part of the global carbonate budget such that about a third of the solid surface of the planet is covered with a drape of sediment composed largely of planktonic foraminifer shells and the remains of other mineralizing plankton [1]. This sediment accumulates slowly–typically at the rate of a few centimetres per thousand years–but can build up continuously for millions of years, attaining thicknesses of hundreds of metres. Coring of these sediments worldwide has produced a uniquely detailed and exceptionally complete fossil record, making the group important for studying patterns and processes of micro- and macroevolution [2].

Here we examine the fossil record of *Pulleniatina*, one of the most common genera of planktonic foraminifera in the tropical oceans. We focus on an enigmatic feature of the record, namely that there are frequent significant fluctuations in the dominant coiling direction of the lineage through time. Sediment cores from the Pacific and Indian Oceans recently recovered by International Ocean Discovery Program (IODP) Expedition 363 allow us to study the phenomenon in greater detail than has hitherto been possible. We seek to better characterise the temporal and spatial pattern of coiling ratio variation in *Pulleniatina* and investigate whether significant morphological and/or habitat differences existed between left and right coiling forms in the past. Our fundamental objective is to understand the mechanism behind these coiling reversals and to explore its evolutionary implications.

### Species concepts

In this paper we consider issues related to species and evolution in the fossil record, a field in which there is potential for terminological confusion, hence it is important to define some terms at the outset. For the purpose of this paper we adopt Barraclough’s recent definition of the species as “an independently evolving group of organisms that is genetically and phenotypically distinct from other groups” (p. 7 in [3]). This definition is a carefully reworded version of Simpson’s [4] classic ‘evolutionary species concept’. The definition is broad and, we believe, useful, because it can be applied to fossil as well as living populations and it leaves open the interesting question of what processes maintain the cohesion of evolving species in the long term. Because the definition requires phenotypic distinctiveness, it follows that reproductive isolation of a population is not in itself sufficient to define a species [3]. Another important part of the definition is the present participle “evolving” which implies an ongoing process that is captured in a snapshot of time. A third significant aspect of the definition is that it refers to entities in nature and not to taxonomic units at the species level. All this has implications for how we apply the species concept to the fossil record.

Because species are required to be morphologically distinct from one another in Barraclough’s definition, morphology can be used as a means of identifying individuals to the correct taxon at species level. A fundamental aim of biological systematics is to describe the diversity that exists in nature. By the rules of zoological nomenclature, each named species-taxon must be defined by a unique Linnean binomial which is associated with a curated type specimen. We can reasonably aspire to achieve a one-to-one match between the number of species that actually exists in nature and the number of species-taxa that are formally recognized according to the rules of systematics. Biologists currently recognize about 50 living species-taxa of planktonic foraminifera by their clearly distinguishable shell morphology [1]. The taxonomy of planktonic foraminifera is very mature and the diversity is relatively modest such that we do not expect any new biological species to be discovered in the future except insofar as there is always scope for either ‘splitting’ or lumping’ when interpreting what constitutes “phenotypically distinct” in the definition.

Genetic studies have revealed a large amount of diversity within most (but not all) biological species, leading to claims that there are multiple ‘cryptic species’ in the ocean, typically between two and ten per biological species currently known [5–14]. These genotypes may be reproductively isolated from each other and it is suggested that some may have deep (millions of years) estimated divergence times in the past [6, 7, 11]. Subtle morphological differences may be associated with them [5, 6] but not enough for them to be considered morphologically ‘distinct’ as required by the species definition adopted herein, although there may be borderline cases [6]. Similar observations have been made on other marine plankton such as diatoms [15] and radiolaria [16] and in many other types of organism [17]. We refer to these units as ‘cryptic genotypes’ insofar as species cannot be cryptic according to the definition we use.

Barraclough’s species definition refers to “independently evolving” groups of organisms. The fossil record can potentially add information here because it records the long-term temporal dimension of the evolutionary process. However, complications arise when populations are traced through the sedimentary record over evolutionary time, potentially over millions of years, as is the case with planktonic foraminifera. In this study, the evolutionary lineages that lead up to modern species are referred to as ‘species-lineages’ or just ‘lineages’ following Simpson [4]. Species-lineages are in effect the branches on the phylogenetic tree which, in the planktonic foraminifera, by virtue of the excellent fossil record, can be traced through the strata and do not need to be indirectly inferred, for instance through the methods of cladistics analysis [18]. Such lineages may branch in speciation events, when one phenotypically distinct entity becomes two [19], and any amount of permanent evolutionary modification can in principle occur through time [2]. Equally, lineages may go extinct and disappear from the stratigraphic record altogether [2].

The formal rules of the International Code of Zoological Nomenclature (www.iczn.org/code) are widely applied to fossils in the same way as to living species but the fact of morphological evolution presents a particular challenge in paleontology. Whereas a classification scheme of typologically defined species-taxa might correspond to a set of real-world species at any moment in time (such as the present, as discussed above), evolution in effect provides a permanently moving target for systematics in the fossil record. The description of species-taxa and development of a coherent taxonomy is a historical process that often begins with sparse information and proceeds with ever more detailed geographical and temporal resolution. The end result of this process is a tendency to fill in the gaps revealing species-taxa that chop evolving lineages up into arbitrary segments [20]. Such taxonomies are ubiquitous in palaeontology and useful, for instance in biostratigraphy. We refer to the formally defined species-taxa of the fossil record as ‘morphospecies’ and uphold a fundamental distinction between that concept and the evolutionary lineage [2, 20]. The first and last occurrences of morphospecies in the fossil record are best thought of as taxonomically defined waypoints, often arbitrary in the sense that different workers could operate different taxonomic schemes with equal effectiveness.

### Previous work on *Pulleniatina* and its coiling

**Fig 1:**
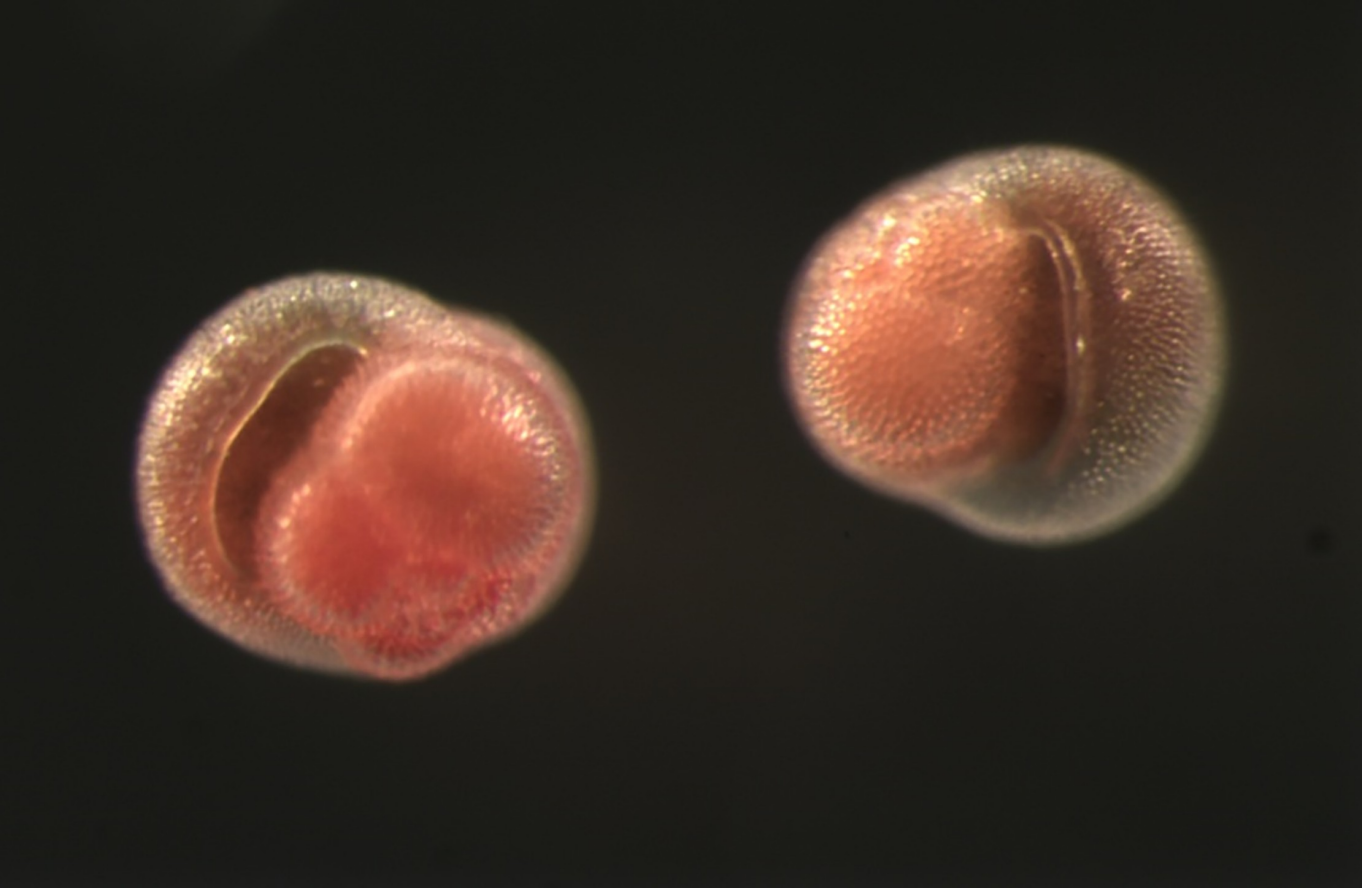
Live *Pulleniatina obliquiloculata* from the Sargasso Sea. Width of specimens approximately 400 μm (credit: Colomban DeVargas).

*Pulleniatina obliquiloculata* (Fig 1) is a non-spinose planktonic foraminifer that is very abundant in tropical and sub-tropical waters worldwide [21]. It is generally regarded as the only living species in the genus. Plankton tow (e.g. [22, 23]) and geochemical (e.g. [22, 24]) data indicate that it exploits subsurface thermocline environments, typically 50–150 m deep, depending on local conditions and seasonality. It is herbivorous, feeding on diatoms and chrysophyte algae [25] which often peak in abundance at the deep chlorophyll maximum (DCM) where upwardly diffusing nutrients encounter the base of the euphotic zone, stimulating primary production.

The DCM is a dynamic and complex environment in which phytoplankton and zooplankton groups including other planktonic foraminifer species are vertically stratified [26]. Sediment trap counts suggest that *Pulleniatina* abundance varies markedly with oceanographic conditions and seasonal fluctuations, with a typical production of dead specimens sinking out of the DCM into the deep ocean in the region of 20 shells per m^2^ per day (e.g., [27–30]). The life cycle of most species is tied to the lunar cycle, so a simple calculation across the area of the tropical oceans produces a very rough estimate of the standing stock at 10^16^ individuals. The actual numbers are likely to be higher, depending on poorly known factors such as juvenile mortality rates and predation by organisms such as tunicates, pteropods, and crustaceans, as well as dissolution in the water column [31].

Like other planktonic foraminifera, the shells of modern *Pulleniatina* are composed of successive chambers. Early chambers are added in a regular trochospiral, but in the adult form the coiling axis changes, becoming oblique to the original axis, producing a chamber arrangement that has been called ’streptospiral’ [32]. This character is the reason for the specific name *obliquiloculata* (p. 368 in [33]). In some large specimens the final few chambers seem to settle into a new plane of coiling that engulfs the earlier whorls at approximately right angles to the initial trochospiral. In such forms the outer part of the test can appear virtually planispiral and bilaterally symmetrical. This near-planispiral morphospecies has been called *Pulleniatina finalis* in the fossil record and is useful for biostratigraphy [32] but there is currently no evidence that it defines a distinct biological species. Instead it seems to be an extreme morphology caused by the addition of one or two more chambers than is usually the case. Even in these cases it is generally possible to detect the initial coiling direction by examining the earlier-formed chambers in the region of the aperture. To our knowledge, all modern specimens of *Pulleniatina* are dextrally coiled.

Genetic sequencing has revealed substantial cryptic diversity of the Ribosomal RNA genes, as is the case with most other planktonic foraminifera. Initial investigations led to the suggestion that there were three distinct cryptic genotypes designated Type I, Type IIa and Type IIb [11]. However as more sequences have been published, the clustering into genotypes became less apparent, such that algorithms failed to produce a division of the *P*. *obliquiloculata* sequences into statistically significant sub-groups [9]. A recent review finds “5 clusters and 4 isolated sequences” (Ref [9], p. 7) which may hint that the actual number of distinct genotypes is more than three. On the other hand, the same study suggests that “non-concerted evolution of SSU [Small Subunit Ribosomal RNA gene] copies within genomes may have generated the widespread sorting of clones from the same individuals into different clusters” (Ref [9], p. 10) which would be problematic for identifying genuinely distinct populations. It should be noted that there has not so far been any attempt to investigate whether “*finalis*”-type shells map on to any of the putative genetic clusters.

Despite the possibility that not all the known genetic diversity in the Ribosomal RNA genes is related to separate populations, some or most of it certainly is because the cryptic genotypes are to some extent geographically distinct. Ujiié and Ishitani [11] have shown that Type I occurs widely across the entire tropical oceans, but Type IIa is absent in the Atlantic and Indian Oceans and Type IIb is rare there. Evidently, populations of Type IIb do not survive transit to the Indian Ocean via the Indonesian Throughflow [34] to reproduce in detectable numbers. In the Pacific, Types IIa and IIb have overlapping distributions but Type IIa prefers more equatorial waters [11]. These geographic patterns suggest that a degree of environmental specificity or competitive exclusion currently operates between genotypes of *Pulleniatina*. In this respect, *P*. *obliquiloculata* is like other planktonic foraminifer species in which the different genotypes often show geographic or depth habitat differences [5–10]. Ujiié and Ishitani [11] estimated divergence times between the genotypes using the molecular clock calibrated to the fossil record. This led them to estimate that Type I and Type II diverged as long ago as ~ 3.1 Ma and Types IIa and IIb diverged ~ 1.4 Ma. These surprisingly ancient divergence time estimates are discussed further below in the light of the fossil evidence presented here.

**Fig 2:**
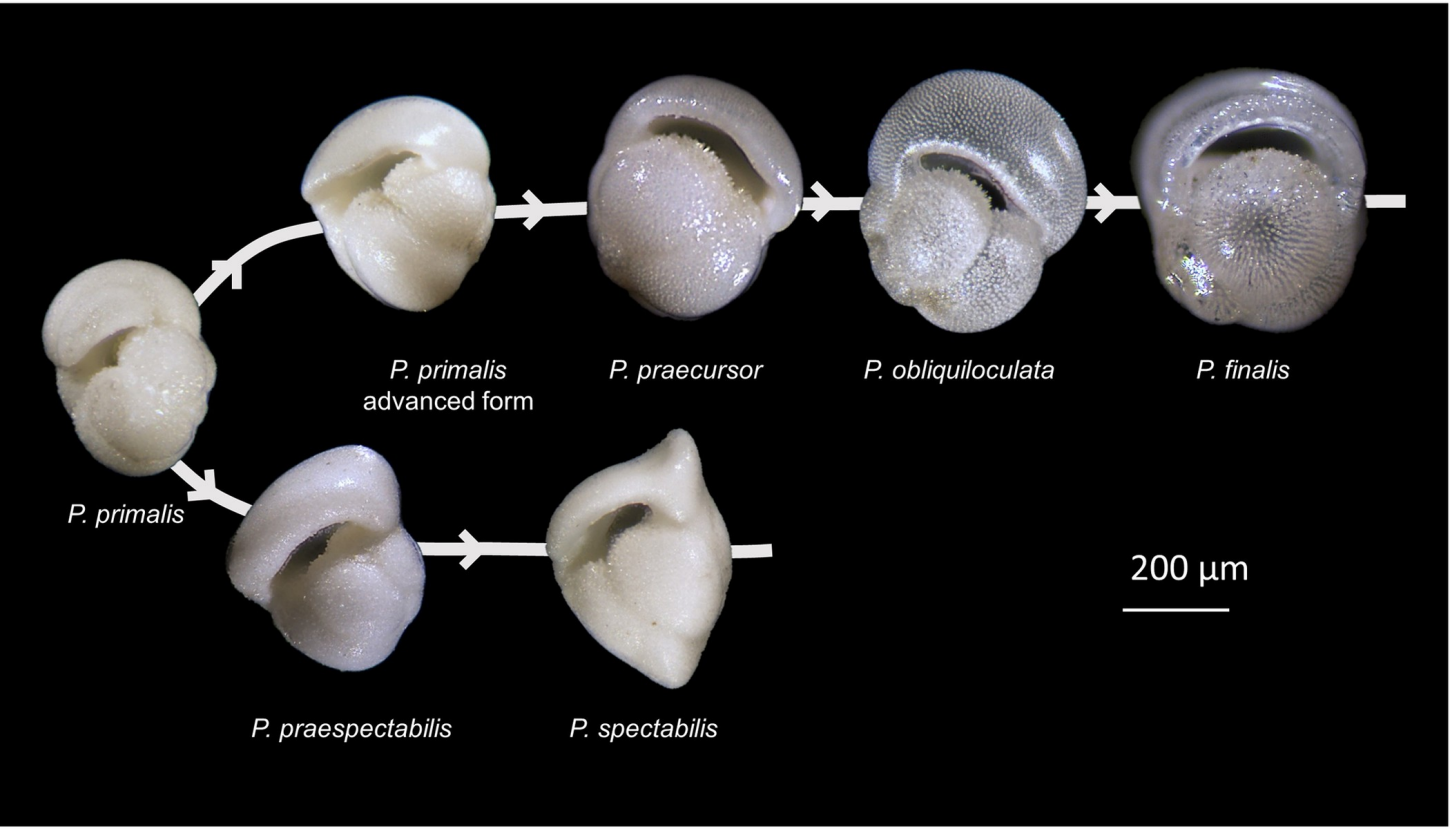
Examples of fossil *Pulleniatina* morphospecies arranged in two lineages. ‘*P*. *primalis*’ from upper Miocene Biozone M13b at level of first appearance approximately 6.4 Ma showing transition from *Neogloboquadrina acostaensis*, Sample U1488A–21H–4, 79–81 cm, Eauripik Rise, western tropical Pacific Ocean. ‘*P*. *primalis* advanced form’ from Pliocene Biozone PL2 (lower part) approximately 4.2 Ma, Sample U1488A–12H–CC, Eauripik Rise, western tropical Pacific Ocean. ‘*P*. *praecursor*’ from Pliocene Biozone PL2 (lower part) approximately 4.2 Ma, Sample 873B–3H–3, 10–12 cm, Wodejebato Guyot, western tropical Pacific Ocean. ‘*P*. *obliquiloculata*’ and ‘*P*. *finalis*’ from Pleistocene Biozone PT1 approximately 0.8 Ma, Sample U1483A–11H–2, 50–52 cm, off northwest Australian margin, eastern tropical Indian Ocean. ‘*P*. *praespectabilis*’ from Pliocene Biozone PL1 approximately 4.8 Ma, Sample 873B–3H–5, 10–12 cm, Wodejebato Guyot, western tropical Pacific Ocean. ‘*P*. *spectabilis*’ from Pliocene Biozone PL2 (lower part) approximately 4.2 Ma, Sample 873B–3H–3, 10–12 cm, Wodejebato Guyot, western tropical Pacific Ocean. Photographs obtained with a z-stacking light microscope.

Although the very deep ocean >~4.5 km is corrosive to calcium carbonate, many sites have been cored on submarine plateaus, ridge flanks and seamounts where planktonic foraminifer shells are preserved. Fossil *Pulleniatina* occurs continuously at high abundance in tropical sediments of the Indo-Pacific for its entire 6.4 million year stratigraphic range. In the Atlantic Ocean, in contrast, *Pulleniatina* became locally extinct at 3.41 Ma and was almost entirely absent until 2.26 Ma, after which it was permanently established once again [35]. Populations of hundreds of specimens can be sampled at will by washing a few cubic centimetres of sediment from any level in many of the existing deep-sea cores. The fossil record of *Pulleniatina* has been studied intensively since the 1960s, revealing significant morphological evolution which has allowed subdivision into a number of formal taxa that have biostratigraphic utility [32, 36–38]. The taxonomy preferred here includes six such morphospecies arranged in two lineages (Fig 2).

**Fig 3:**
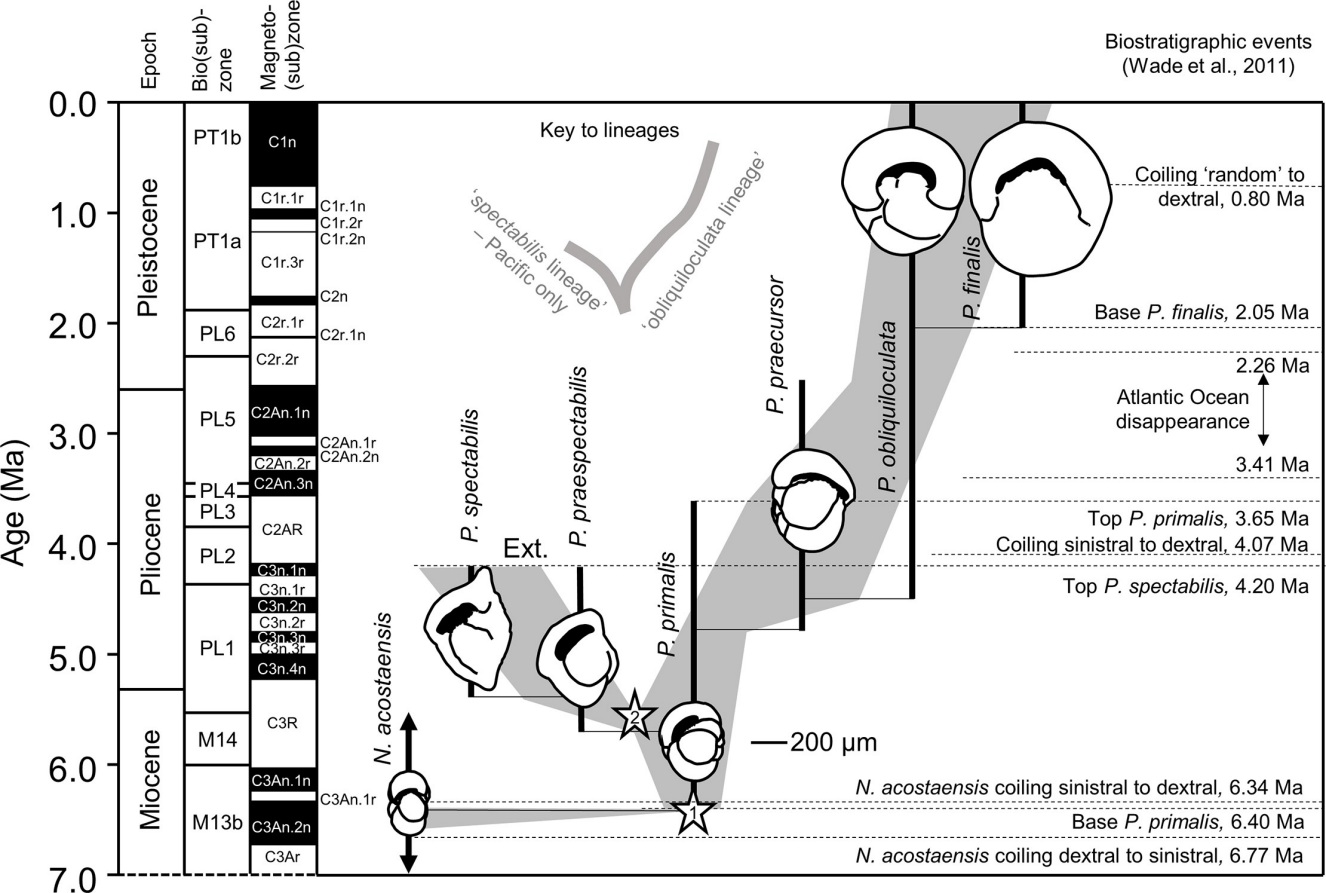
Stratigraphic record of *Pulleniatina* according to the standard taxonomy, with suggested lineage phylogeny. Vertical bars represent the stratigraphic ranges of named morphospecies. Horizontal lines represent supposed ‘evolutionary’ relationships between morphospecies. Grey shading represents apparent intergradation between morphospecies. Stars represent lineage cladogenetic events: 1, when *Pulleniatina primalis* diverged from *Neogloboquadrina acostaensis* and 2, when the *spectabilis* lineage diverged from the main lineage. ‘Ext.’ represents extinction of the *spectabilis* lineage. Biostratigraphic zones and events are from the compilation of Ref [39]. Foraminifer sketches show typical specimens in apertural view, to scale.

The paleontological taxonomy of *Pulleniatina* is explored in Fig 3. This emphasizes the distinction between morphospecies and lineages and that the origin and disappearance of morphospecies cannot generally be equated with biological speciation if a species is “an independently evolving group of organisms” [3]. That would only be possible if the fossil record exhibited an extreme version of punctuated equilibrium with instantaneous appearances, but the many morphometric studies that have been conducted on planktonic foraminifera and other fossil plankton show that they tend to blend and intergrade gradually with other morphospecies through time, forming ‘chronoclines’ (ref [19] and references therein). At any one time a species lineage may consist of two or more intergrading morphospecies. The appearances and disappearances of morphospecies are sometimes called ‘pseudospeciations’, and their last occurrences are ‘pseudoextinctions’ [20]. Of course it may be that a morphospecies originates at a moment of branching and therefore effectively coincides with a true speciation (as appears to be the case with *Pulleniatina primalis*), or that its disappearance coincides with a true extinction (as with *Pulleniatina spectabilis*), but that cannot be assumed.

The genus *Pulleniatina* is distinguished from related non-spinose planktonic foraminifera by the presence of a smooth and reflective thickened shell surface (cortex) in adult forms. This feature first appeared in the late Miocene around 6.4 Ma [40]. The earliest *Pulleniatina* show a clear affinity with the extinct morphospecies *Neogloboquadrina acostaensis*, from which the genus is deemed to have branched off in a biological speciation event (cladogenesis) [32, 40]. After this divergence, the first morphospecies of the genus, known as *P*. *primalis*, persisted alongside its ancestor, the *Neogloboquadrina acostaensis* lineage. The latter is also thought to be have been ancestral, by an earlier cladogenetic event, to a lineage leading to the three modern biological species of *Neogloboquadrina* [2].

In the continuous Indo-Pacific record there is a gradual morphological trend from smaller *Pulleniatina* specimens with regular trochospiral chamber addition, more chambers per whorl and low arched apertures, to larger more tightly coiled, involute and spherical forms with a tendency for the later chambers to be added in a more streptospiral arrangement, and with the aperture frequently being more broadly arched and extending on to the spiral side [32]. This chronocline encompasses the morphospecies *primalis* > *praecursor* > *obliquiloculata* > *finalis*. However a different, peripherally flattened and pinched morphospecies (*P*. *spectabilis*) is also known, restricted to the tropical Pacific Ocean of the latest Miocene and early Pliocene [36]. It is very distinctive and different from specimens of the main lineage that co-occur in the same samples. However *P*. *spectabilis* populations can also be traced back through time via the intermediate morphospecies, *P*. *praespectabilis*, to fully intergrade with *P*. *primalis*. The *spectabilis* lineage disappeared abruptly at 4.20 Ma [39] in what appears to be a genuine extinction.

The evolutionary lineages shown with grey shading in Fig 3 depict the range of morphological variation that is seen in fossil populations through the stratigraphic record. These observations are at present qualitative, but are amenable to testing with morphometric methods [41]. One moot point however is whether ‘species’ in their temporally extended concept as evolutionary species-lineages should be considered as terminating at phylogenetic branching points or can persist through them. In the case of the first cladogenesis associated with the origin of *P*. *primalis*, the event is a relatively sharply defined appearance through which the ancestral *N*. *acostaensis* lineage persists seemingly unchanged (i.e., a budding pattern). The second cladogenesis on the other hand is a more gradual divergence of *P*. *primalis* into *P*. *spectabilis* and *P*. *praecursor* (i.e., a bifurcating pattern). This problem of nomenclature is discussed in more detail elsewhere [2, 42, 43].

**Fig 4:**
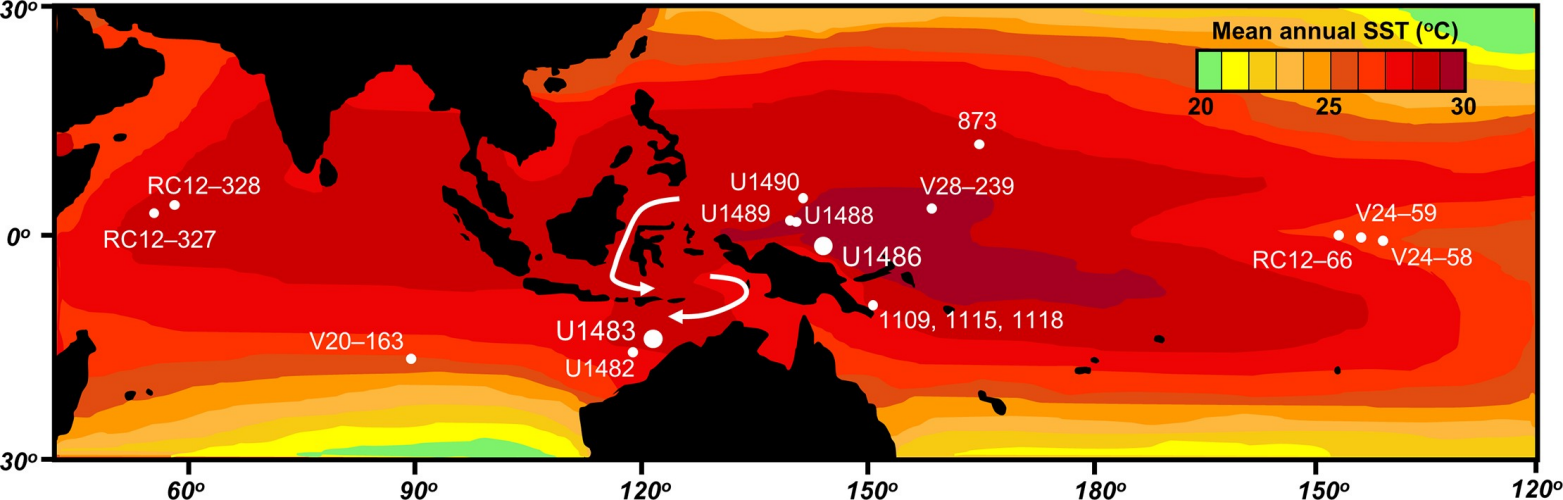
Locations of *Pulleniatina* coiling records discussed in this study. Larger circles denote the two sites from which new data are presented. Arrows indicate path of the main Indonesian through-flow from the Pacific to Indian Oceans. SST = sea surface temperature. Mean annual temperature data: NASA, redrawn.

**Fig 5:**
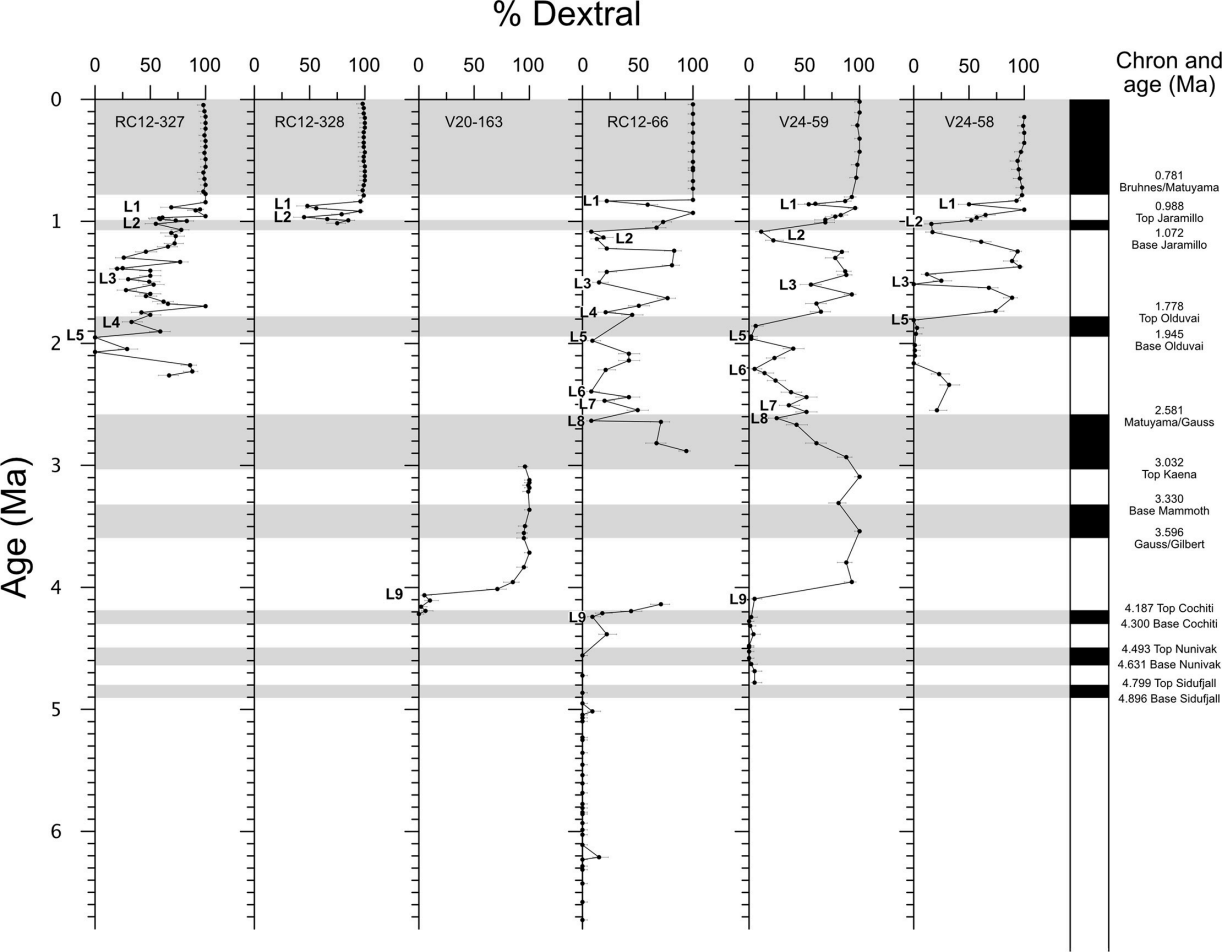
Saito’s ‘L’ events in six Indo-Pacific cores. For locations see Fig 4. Redrawn from Ref. [46] on a modern timescale and with sampling error bars added. Error bars represent 95% confidence intervals about the measured proportion. Labels for ‘L’ events are placed close to where they were originally indicated in [46].

Coiling direction in *Pulleniatina* has been used as a stratigraphic tool since the 1960s [44, 45]. The field was advanced in 1976 by Saito [46] who studied the coiling record in a set of piston cores through the tropical Atlantic, Indian and Pacific Oceans relative to a series of magnetic reversals that provide a common stratigraphic framework. Saito found that although Atlantic populations show variations in the dominant coiling directions in the period since the re-colonization of that ocean about 2.26 Ma, these do not match the variations seen across the Indian and Pacific Oceans. He attributed this (reasonably, we think) to the semi-isolation of the Atlantic from the tropical Indo-Pacific following the closure of the Isthmus of Panama in the late Pliocene. The first (down-core) interval of abundant sinistral specimens in Saito’s Indo-Pacific records occurs at a level below the Brunhes/Matuyama magnetic reversal at about 0.8 Ma. Below this, Saito found a series of prominent left-coiling intervals with trans-oceanic correlation potential. He named these ‘L’ (for left) intervals and numbered them from the top down, although we note there is some ambiguity as to whether he was labelling prominent ‘shifts’ into or out of left coiling, or the left coiling intervals themselves. Saito’s sites are shown on Fig 4 in relation to other key locations discussed in this paper. We have re-drawn Saito’s plot on an up-to-date timescale [63] and added sampling error bars on the ratios in Fig 5.

While it is clear that the six Indo-Pacific coiling records shown by Saito [46] have much in common, but given the down-core sampling resolution of his study it is not obvious that all or even most of the ‘L’ intervals are securely identified and correlated. Consider, for instance, ‘L2’–this is indicated around the top of the short Jaramillo subchron in some records, but below it in others. If significant diachrony is involved in the correlations because of the process by which genes or populations spread around the oceans and become established, it would be even more difficult to define and correlate these ‘L’ intervals with confidence. These limitations became evident when Thompson and Sciarillo [47] attempted to apply Saito’s scheme to another core site. In doing so they divided Saito’s ‘L2’ into two distinct peaks ‘L2a’ and ‘L2b’ while also finding even larger sinistral peaks between ‘L3’ and ‘L4’ that Saito had not identified. They labelled ‘L6’ as occurring within the paleomagnetic ‘Olduvai normal’ interval whereas Saito had placed it hundreds of thousands of years earlier.

The most detailed *Pulleniatina* coiling records previously published are from a study by Resig et al. [48] on three Ocean Drilling Program (ODP) sites in the Woodlark Basin southeast of Papua New Guinea. That study identified seven of Saito’s nine left-coiling intervals, the exceptions being ‘L4’ and ‘L7’, neither of which is very obvious in Saito’s original data (Fig 5). These were calibrated to the paleomagnetic timescale, but the calibration ages indicate significant diachrony. For instance, the top of ‘L1’ is located at ODP Site 1109 at 0.65 Ma but at Site 1115 it is at 0.84 Ma, despite the two locations being less than 10 km apart and sampling the same water column [48]. The problem may lie in the fact that the top of ‘L1’ at Site 1109 is the top of a slumped interval containing reworked foraminifera. Examination of the data from Resig et al. [48] shows that some of the identified ‘L’ intervals have significant dextral excursions within them and thus the terminology appears stretched to the limit, especially as none of the authors discussed up to this point plotted sampling error bars on their figures.

The most recent advance in the field is a re-study of the record at ODP Site 1115 by Chiang et al. [49] in which the absolute abundances of left and right coiling shells in the sediment were measured (as opposed to merely their ratio) across two prominent intervals that were identified as ‘L5’ and ‘L6 ‘in Saito’s scheme. Significant changes in absolute abundances occurred in both instances. A similar strategy was adopted independently by us as described in the next section.

## Methods

IODP Expedition 363 sailed in October-December 2016 to investigate the paleoceanography of the Indo-Pacific Warm Pool, an area with very abundant *Pulleniatina*. One of us (PNP) served as shipboard planktonic foraminifer biostratigrapher. From the beginning of the expedition it was decided to gather as much information on *Pulleniatina* coiling directions as time would allow to determine to what extent Saito’s ‘L’ events can be usefully correlated in the new sites, especially between the Pacific and Indian Oceans, as much of the scientific interest in the expedition relates to the Indo-Pacific through-flow. Initial shipboard data and coiling plots are available in the individual site reports for IODP Sites U1482, U1483, U1486, U1487, U1488, U1489, and U1490 [50–56].

It became evident from the shipboard work that a much higher resolution record of *Pulleniatina* coiling with a temporal resolution that approaches the mixing time of the ocean (a few thousand years) would be desirable to serve as a reference frame for future studies. Tropical Pacific Site U1486 (2°22.34ʹS, 144°36.08ʹE) lies north of New Guinea and ~215 km west-southwest from Manus Island in a water depth of 1332 m (see Fig 4). Foraminifer preservation is very good to excellent [52] although significant mechanical fragmentation was observed in the lower part of the hole where the water depth may have been considerably shallower than it is today. Apart from this, Site U1486 is an excellent place to develop such a record because of its abundant *Pulleniatina* populations, high sedimentation rate (averaging 6.9 cm/k.yr), lack of soft sediment deformation, and well-developed shipboard magnetostratigraphy back to the Matuyama / Gauss reversal at 2.581 Ma [52]. It also provides an opportunity to better understand the population dynamics underpinning the coiling reversal sequence by investigating whether co-occurring sinistral and dextral shells can be distinguished by their mean size and/or ecology as recorded in their oxygen and carbon stable isotope ratios. An ancillary aim was to increase the sampling resolution at Indian Ocean Site U1483 through the interval equivalent to Saito’s ‘L1’ and ‘L2’ intervals to investigate how precisely coiling fluctuations appear to correlate between oceans. IODP Site U1483 (13°5.24′S, 121°48.25′E) is on the northwest Australian margin of the Indian Ocean in a water depth of 1733 m, hence it is ‘down-stream’ from Site U1486 with respect to the Indonesian through-flow (see Fig 4). Planktonic foraminifer preservation is excellent and glassy [51]. The magnetostratigraphy is very good although a problem relating to the placement of the Brunhes / Matuyama boundary arose in post-expedition work and is discussed in the relevant section below.

We requested 440 sediment samples of 10 cm^3^ from throughout the inter-hole spliced stratigraphic record at IODP Site U1486 (to augment the approximately 50 samples from the same interval that had already been counted shipboard). An age model for the site (S1 Table) was established using linear interpolation between seven magnetic reversals that were identified shipboard [52] assuming no significant change in sedimentation rate through glacial-interglacial cycles. Note that the estimated ages of samples are less well constrained when they are far from magnetic reversals. A more detailed age model based on orbital cycles as detected in benthic oxygen isotope ratios is currently under development (S. Bova, personal communication 2020) but a preliminary assessment of this confirms that the age model used here is likely to be very close to the eventual astronomically tuned version. There is no stratigraphic or sedimentological evidence for any hiatus at the site although there are many visible ash bands and we note that in the lower part of the record the amount of volcanic ash increases dramatically down the succession such that the average sedimentation rate is correspondingly higher.

Samples were dried in the oven overnight at 40°C, weighed, and washed in de-ionized water over 63 and 150 μm sieves. The > 63 μm and > 150 μm fractions were dried again in an oven at 40°C and weighed. Sample residues were strewn on a picking tray and systematically scanned. The coiling directions of the first 50 specimens of the > 150 μm fraction of *Pulleniatina* were counted without removing them. The two biostratigraphic morphospecies *P*. *obliquiloculata* and *P*. *finalis* were not differentiated. The choice of counting 50 specimens in each sample was to balance stratigraphic sampling resolution and population sampling error. For each sample the 95% confidence interval about the measured coiling ratio was estimated using the modified Wald method [57] which has the advantage over other methods of allowing asymmetrical error bars for ratios that are biased in one direction or another. Sample weight and *Pulleniatina* coiling data from Site U1486 are available in S2 Table. Shipboard coiling counts in S3 Table are assigned to the same age model and the post-cruise and shipboard data sets are combined in S4 Table. The study was augmented with a further 51 new samples from Indian Ocean Site U1483 through the ‘L1’ and ‘L2’ intervals that were treated in the same way: the age model is given in S5 Table and combined post-cruise and shipboard coiling data in S6 Table.

When the full high-resolution records of coiling counts were completed, five intervals of marked coiling ratio fluctuation were selected for more intensive study. The purpose of this was to investigate whether fluctuations in coiling ratio correspond to changes in absolute abundance of left and right forms. These consisting of between 10 and 16 consecutive samples and are referred to hereafter as ‘windows’. One of these windows (W1) is in Site U1483 and four (W2–W5) in Site U1486. The washed residues from the windows were split two or three times using an ACS Scientific MS-1 microsplitter to produce a manageable residue size from which every specimen of *Pulleniatina* in the final split was counted (the number frequently exceeding 200 specimens). The more accurate estimates of the coiling ratio that resulted from this exercise were then substituted for the original counts of 50 specimens that had previously made from the same samples (which explains why the sampling error bars reduce in the windows). The data from the windows were converted to estimates of the flux of sinistral and dextral specimens to the seafloor per square metre per day using the interpolated sedimentation rate for the core and interval in question (S7 Table).

From each of the five windows we then selected a single sample and picked the first 50 sinistral and 50 dextral *Pulleniatina* specimens using a wet brush. The exception to this was Window 5 where too few dextral specimens were present and additional sinistral specimens were picked instead. Each specimen from the five samples was initially mounted with umbilical side up on a cardboard slide that had been lightly primed with water-soluble glue. Specimens were photographed, and the size (area) measured under a microscope using Image Pro Premier software which automatically finds the outline. Specimens were then removed from the cardboard slides, cleaned of glue in deionized water, and individually analysed for their oxygen and carbon isotope ratios at Cardiff University using a Thermo MAT 253 mass spectrometer with analytical precision of ± 0.05‰ for δ^18^O and ±0.03‰ for δ^13^C. By this method we are able to relate the photographed morphology, size, and the carbon and oxygen isotope ratio of every specimen. In a few cases the data were subsequently discarded because of doubts as to the correct identification. Photographs of all analysed *Pulleniatina* specimens are given in S1 Fig and the size and stable isotope data are in S8 Table.

## Results

### Site U1486

**Fig 6:**
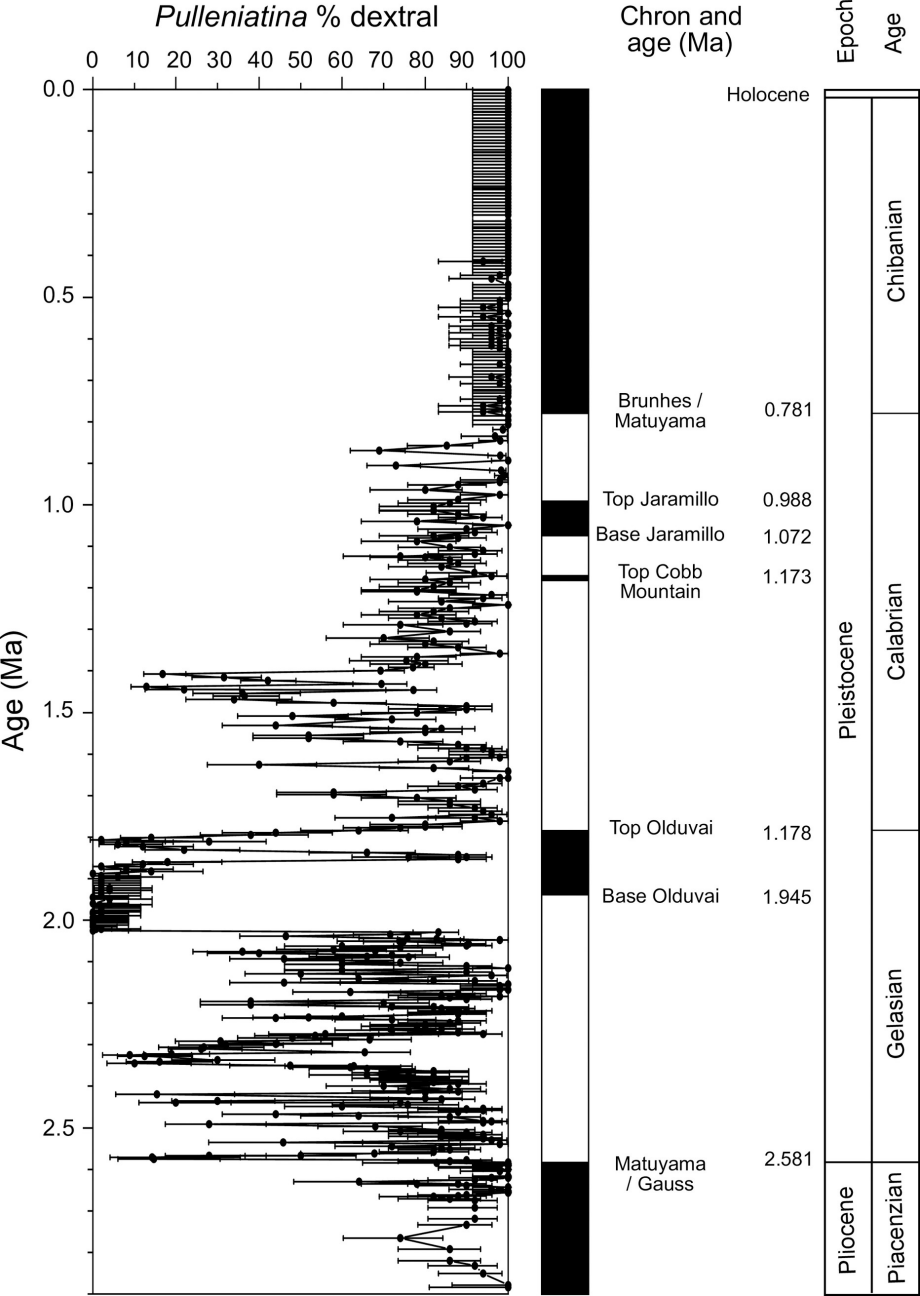
High resolution *Pulleniatina* coiling ratio record at Site U1486. Error bars represent 95% confidence intervals.

The measured coiling record, which combines shipboard and post-cruise samples is shown in Fig 6. It is possible to recognize features that correspond to several of Saito’s ‘L’ intervals but high resolution variability makes precise correlations between records problematic. For this reason, and because of ambiguity in defining coiling ‘events’ and recognizing their start and end points, we do not formally adopt Saito’s nomenclature in our study. Instead, we highlight the following features of the record, going down-core:

1. Shells are exclusively dextral down to 0.423 Ma. This corresponds to a total of 62 consecutive samples and 3,100 measured specimens, indicating that if sinistral individuals are present they must be extremely rare. The interval of exclusively dextral measurements encompasses four major glacial / interglacial cycles and the associated paleoceanographic changes. We note that occasional sinistral specimens have been found at other Indo-Pacific sites by previous authors within this time interval [46, 48] and by us at Site U1483, as discussed below.
2. Occasional sinistral specimens were found (varying from zero to eight percent per sample) from 0.423 Ma to 0.845 Ma.
3. The first peak with > 10% sinistrals consists of two samples calibrated to 0.857 and 0.869 ka, that is, some way below the Brunhes / Matuyama paleomagnetic reversal in a magnetically reversed interval known as Subchron C1r.1r. This is similar to the pattern seen in all of Saito’s sites [46] and others [47, 48] and has been identified as ‘L1’. However the abundance of sinistrals is not as great as in most previously studied sites where they usually peak at over 50%.
4. A second significant peak in % sinistrals was found in just one sample at 0.904 Ma, approximately half way through Subchron C1r.1r. This may or may not correlate with a similar transient feature previously found by Thompson and Sciarillo [47].
5. The interval from 0.95 to 1.35 Ma consists of samples averaging about 85% dextral specimens. Our sampling error is such that it does not resolve into significant peaks. The previously published ‘L2’ [46], and ‘L2a’ and ‘L2b’ [47], excursions lie somewhere in this part of the record but they are impossible to identify or correlate.
6. Two closely spaced and statistically significant peaks in % sinistral occur around 1.4 Ma. These are approximately the position of Saito’s ‘L3’ [46] which, however, cannot be correlated with confidence.
7. There are four significant fluctuations in % sinistral between 1.5 and 1.7 Ma. There are no ‘L’ events in this interval to correlate them with.
8. There is a well-defined % sinistral peak around 1.8 Ma. The top of the Olduvai normal subchron occurs in the (down-core) transition into this peak. This corresponds approximately to the top of ‘L5’ in some of Saito’s records [46]. Below this, there is a well-defined dextral excursion then a relatively long interval of sinistral dominance that extends down to approximately 2.03 Ma. This part of the record contains within it a run of eight exclusively sinistral populations. It appears to be part of ‘L5’ in Saito’s scheme [46] but is similar to a sequence that is labelled as ‘L6’ in another study [48].
9. The sharpest and most statistically significant feature of the record is the (up-core) transition from dextral to sinistral dominance at the bottom of the above-mentioned sinistrally dominated interval. This was very rapid and took less than 3 k.yr.
10. The interval below this transition contains about ten statistically significant sinistral peaks, most of which are very transient. This is the interval of Saito’s ‘L6’ through ‘L8’ excursions [46] but they cannot be correlated to our reference section with any confidence.
11. Our record does not extend down as far as Saito’s ‘L9’ interval [46].
12. In some intervals (particularly from 0.8 Ma to 1.8 Ma) the record shows possible cyclicity on frequencies of around 100 k.yr and 40 k.yr that may be related to astronomical forcing, but no statistically significant power was found in a simple power spectrum of the record as a whole. The possibility of astronomical/climatic forcing is something that we intend to address further when stable isotope and other geochemical time series from the core become available.

### Site U1483

**Fig 7:**
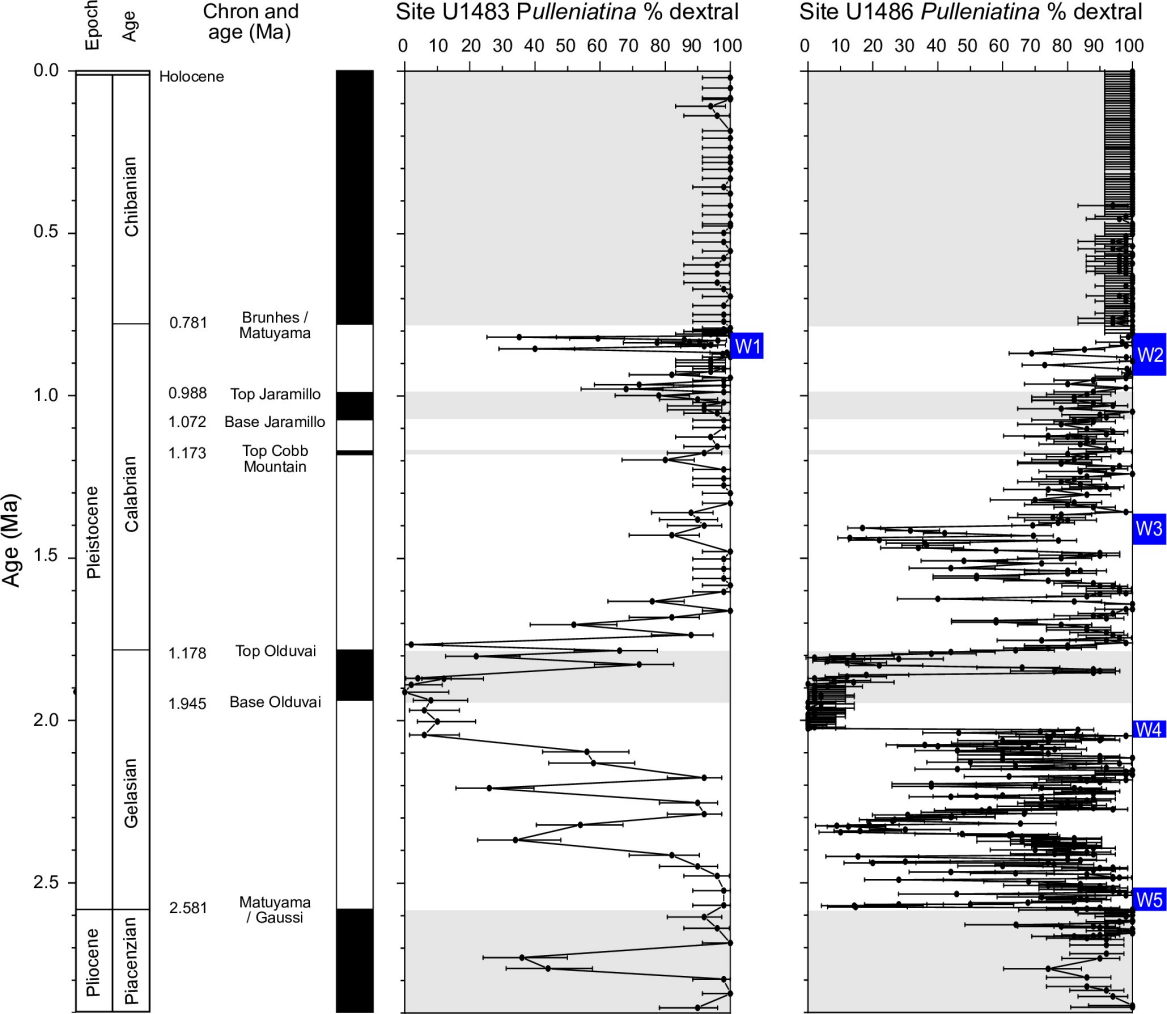
The *Pulleniatina* coiling records at Indian Ocean Site U1483 and Pacific Ocean Site U1486 compared. Error bars represent 95% confidence intervals. Blue bars represent Windows 1–5 in areas of interest that were chosen for more detailed study. Note that the Brunhes / Matuyama boundary and top of Cobb Mountain normal interval were located only at Site U1486.

A series of seven magnetic reversals were found down to the Matuyama / Gauss reversal at 2.58 Ma [51]. However, it became apparent during post-cruise discussion (R. Hatfield personal communication, 2019) that one of these reversals, the supposed Brunhes / Matuyama boundary as placed shipboard, was probably a diagenetic artefact and so is excluded from our revised age model. This is unfortunate for our study because it reduces the precision of correlation in the targeted interval. In Fig 7 we show the shipboard coiling record which is supplemented here by additional sampling at high resolution in the interval 0.75–1.20 Ma (which was initially chosen because of the likelihood of very high precision correlation between the Pacific and Indian Ocean sites). The record is calibrated against the paleomagnetic reversal record by linear interpolation and plotted alongside the Pacific record from Site U1486. Plotting the two records side-by-side allows us to consider the effect of the Indonesian through-flow on the coiling pattern. This is important because modern genotypes of *Pulleniatina* differ in the Pacific and Indian oceans [11]. Despite this, the two records show many similarities. The following features are apparent (proceeding down-hole):

1. Occasional sinistral specimens were found in three samples from the last 400 k.yr at Site U1483 whereas no sinistral specimens were found in the Pacific site in this interval.
2. There are two closely spaced peaks in the vicinity of Saito’s ‘L1’ event. The lower peak coincides within error with the first down-hole peak in Site U1486. An intriguing possibility is that the Site U1483 lags the Pacific site by about 40 k.yr which, although it is a much longer interval than it takes for water to traverse the Indonesian gateway, might plausibly be the time that a new population needs to establish itself in significant numbers within the Indian Ocean subtropical gyre so as to become visible. However, the time series are currently too low-resolution to address possible leads and lags statistically.
3. Fewer sinistrally dominant samples in the interval from 1.3–1.8 Ma were recorded at Site U1483 than at U1486 although that could be due to the low (shipboard) sampling resolution in that interval.
4. The coiling succession around the Olduvai normal interval is similar to Site U1486, but with a slight lag of ~40 k.yr insofar as there is a sinistral peak above the Olduvai subchron at U1483 whereas it is below the magnetic reversal at Site U1486.
5. The record below 2.0 Ma cannot be correlated between our two records with confidence because of the lower sampling resolution at Site U1483 and the fact that it suffers from soft sediment deformation and mixing of foraminifer assemblages from this point onward to the bottom of the hole.

In summary, it is clear that *Pulleniatina* coiling is useful for stratigraphic correlation between sites but there is also significant potential for false correlations of supposed peaks if sampling resolution is low. There is an indication that the Indian Ocean record lags the Pacific by ~20–40 kyr in places but this would need to be investigated with more high-resolution sampling at Site U1483, ideally supported by other sites.

### Investigating critical intervals

**Fig 8:**
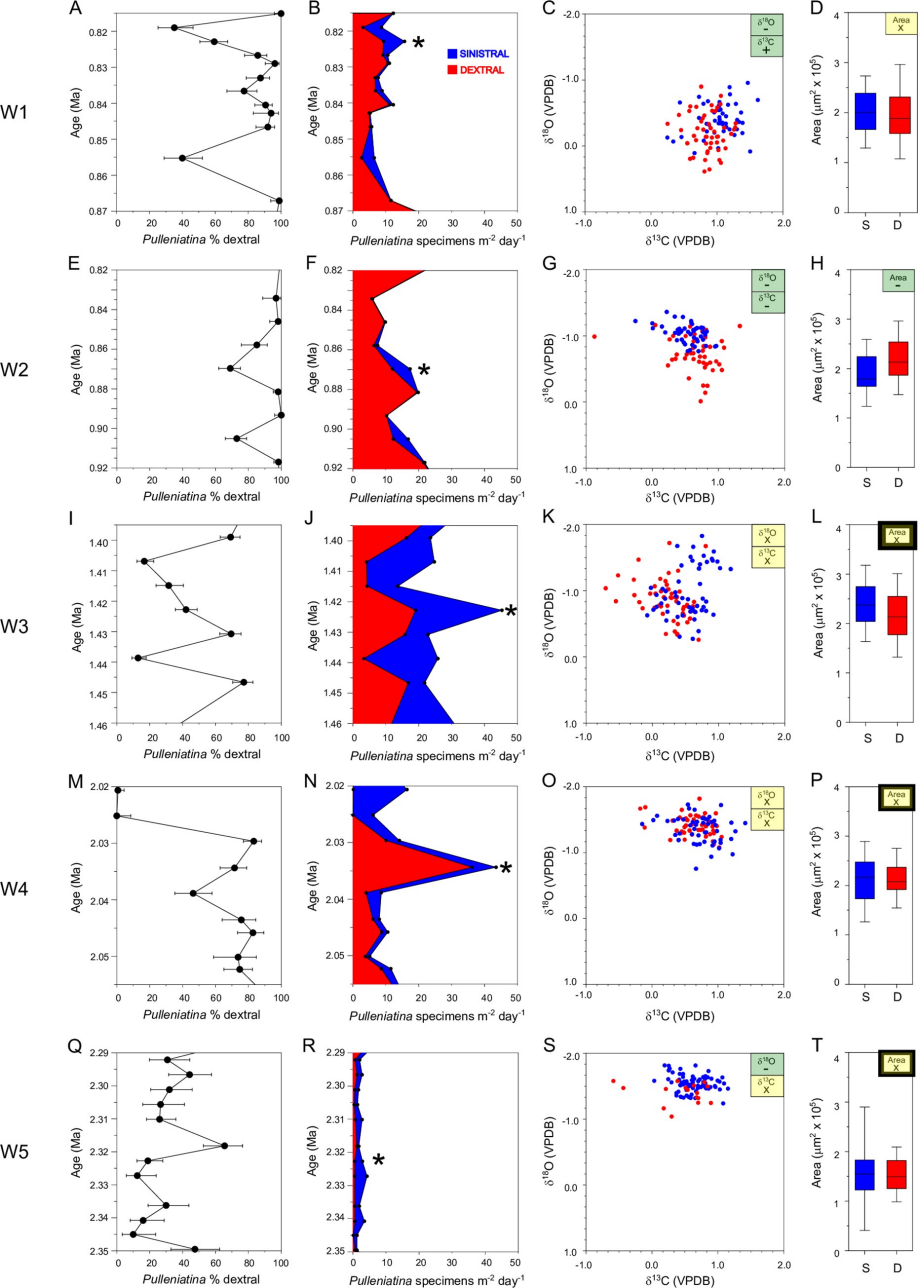
The coiling record in five intensively studied time windows at IODP Sites U1483 (W1) and U1486 (W2-W5). See Fig 7 for the location of these windows within the records. (a) (e) (i) (m) (q), detail of the coiling ratio record, error bars represent 95% confidence intervals. Asterisks indicate the sample chosen for more detailed study. (b) (f) (j) (n) (r), absolute accumulation rate of dextral (red) and sinistral (blue) added cumulatively to give total *Pulleniatina*. (c) (g) (k) (o) (s), stable isotope analyses of individual *Pulleniatina* shells from the sample level highlighted with asterisks. Blue = sinistral, red = dextral. Highlight boxes indicate whether the stable isotope ratios of sinistral specimens are significantly more positive (+) or negative (-) than dextral specimens at 95% confidence, highlighted in green, or if there is no significant difference (x), highlighted in yellow. (d) (h) (l) (p) (t), size distributions of sinistral (blue) and dextral (red) *Pulleniatina* specimens. Bar = mean, box = standard deviation, whiskers = total range. Highlight boxes indicate whether sinistral specimens are significantly larger (+) or smaller (-) than dextral specimens at 95% confidence or if there is no significant difference (x).

As described in the Methods section, five windows of interest in the record were selected because they showed significant fluctuations in the long-term coiling record. Data from each of the windows are shown in Fig 8 which shows the measured coiling ratio, an estimate of the absolute flux of left and right coiling shells to the sea floor, a comparison of stable isotope ratios of ~100 shells in one of the samples from the window, and a comparison of the measured area (size) of those specimens.

Estimates of the absolute fluxes of *Pulleniatina* to the sea floor assumes that all specimens are preserved and not dissolved or fragmented. The study sites are well above the calcite compensation depth and there is little sign of dissolution but mechanical damage is very common in the lower part (Window W5) of Site U1486. Flux estimates vary between about 10 and 50 specimens m^-2^ day^-1^ with typical values of about 15 m^-2^ day^-1^. These fluxes are comparable to deep sediment trap studies; for instance, an average of about 30 *Pulleniatina* m^-2^ day^-1^ was found in the Kuroshio current area of the West Pacific [28] and around 20 m^-2^ day^-1^ in the northern Gulf of Mexico [29]. In W5, absolute abundances are much lower than in the other windows which may be a local feature at Site U1486 caused by a shallower sea floor at that time, mechanical damage, and a more hostile environment as indicated by a high prevalence of volcanic activity and ash.

It is difficult to make a statistically robust judgment as to whether there is any anti-correlation between left and right abundances as would be expected if there was a single combined environmental carrying capacity for both types. Qualitatively, however, the record hints that there may have been a relationship, particularly in W4 where the major coiling shift is caused by the disappearance of dextral specimens, after which there is an increase in the abundance of sinistrals. This crash in dextral abundance correlates to part of the interval previously studied at ODP Site 1115 to the east of Papua New Guinea [49]. That study also found a rapid decline in the absolute abundance of dextral specimens followed by an increase in the abundance of sinistrals at a correlative level.

The spread of oxygen and carbon isotope values of individual *Pulleniatina* shells is similar to that seen in Recent sediment samples [58]. Oxygen isotopes are likely correlated inversely with ambient temperature because of depth or seasonality [59]. Carbon isotope differences may also reflect depth and seasonal variation and also metabolic rate effects such as variable incorporation of respired carbon into the shell [60]. We found no significant correlation between isotopic composition and size in any sample (analysis not shown). Several of the samples show significant differences in the stable isotope ratios of either the carbon or oxygen isotopes (or both) between sinistral and dextral shells but the pattern between samples is not consistent. For instance, the sinistral specimens in W1are slightly more positive in δ^13^C than the dextrals, but the opposite is the case in W2. In W1, W2 and W5 the sinistrals are more negative in δ^18^O, possibly indicating warmer water, and the same may be the case in W3 where the data hint at two clusters, the one more negative in δ^18^O having a higher proportion of sinistral specimens, although overall the difference between sinistral and dextral shells is not statistically significant in this sample. In only one sample, W2, was there a significant difference in size, with the sinistrals being slightly smaller on average. These differences likely point to subtly different (but overlapping) habitat preferences of sinistral and dextral forms in depth, time of year, or in response to inter-annual variations.

## Discussion

### Coiling direction in foraminifera: The bigger picture

The coiling ratio variation in *Pulleniatina* is not an isolated phenomenon; other foraminiferal lineages show similar patterns. Before moving to an explanatory model for the observed variation it is necessary to consider what may be learned from other biological groups and what is known from other genera.

Left-right asymmetry is very common in biology and generally has a heritable genetic basis [61], but the co-occurrence of both enantiomorphs of a chiral body-form in a single species (sometime called “genetic antisymmetry”) is actually quite rare in nature [61, 62]. It has been suggested that long-term maintenance of genetic antisymmetry within a reproductively isolated population requires some form of adaptive mechanism, otherwise one or other enantiomorph would soon become established to the exclusion of the other through drift [63]. Antisymmetry is known in some groups of plants [64], cichlid fishes [65] and tree snails [66]. In those cases, it appears that maintenance of a particular balance between sinistral and dextral forms is due to evolutionary stable strategies arising from the benefits of out-crossing or dis-assortative mating. However, because reproductive behaviour is more simple in planktonic foraminifera, that class of explanation is unlikely to apply in this case.

It is very common for modern species of both planktonic [67] and benthic [68] foraminifera to comprise both sinistral and dextral specimens. The following common patterns have been observed in different planktonic foraminiferal lineages on multi-million year timescales, all of which must ultimately be explicable if the problem of coiling in foraminifera is to be resolved:

1. Lineages in which the coiling direction is always close to random or ‘proportionate’; e.g. *Acarinina soldadoensis* [69].
2. Lineages in which the coiling direction is always markedly biased left or right; e.g. *Acarinina wilxoxensis* [69].
3. Lineages which tend to begin with approximately random coiling but eventually become either left or right dominant; e.g. *Globotruncana fornicata* [66], *Morozovella velascoensis* [68]; *Paragloborotalia mayeri* [69, 70].
4. Lineages which show a gradual drift from right to left dominance or vice-versa over very long time periods; e.g. *Turborotalia cerroazulensis* [19].
5. Lineages which show a single relatively sudden reversal from left to right dominance or vice-versa at some time in their long history (e.g. *Morozovella aragonensis* [69]).
6. Lineages in which the dominant coiling direction oscillates repeatedly and often rapidly between left and right dominance, either locally (e.g. *Globorotalia truncatulinoides* [71]) or globally (e.g. *Pulleniatina*, as studied here).

These taxon-specific patterns demonstrate that a component of coiling direction must be genetic and heritable although there may also be a developmental / ecophenotypic element to the coiling ratios as well, at least in some species [68]. Whether the genetic basis is a simple case of different alleles at a single locus as in snails [63] or more complicated is not currently known. A general rule in nature is that asymmetry at the macroscopic level originates with subcellular (e.g. cytoskeletal) asymmetries at the molecular level [62]. In gastropods, for instance, the shell coiling direction has traced back to asymmetric cellular cleavage in the embryo and on to the apparently symmetrical fertilized egg, the implication being that “some cytoplasmic (likely cytoskeletal and chiral) component in the egg is clearly responsible for orienting spiral cleavage and, ultimately, body asymmetry and shell coiling” (p. R475 in [62]). In foraminifer growth, the macroscopic coiling direction becomes apparent when a second chamber (deuteroconch) is added to the first-formed chamber (proloculus), and is maintained thereafter. Not only is the second chamber positioned asymmetrically on the proloculus but its shape is also asymmetrical, including the position of the aperture that defines the start-point for formation of the succeeding chamber. Hence the cell defines anterior / posterior and left /right axes from early growth,

We have observed occasional developmental abnormalities in various species of highly trochospiral foraminifera wherein a secondary aperture on the spiral side is ‘mistaken’ for the primary aperture and the shell apparently grew thereafter by adding chambers outward at both sides. In all such cases that we have seen, the two ends of the shell coil in mirror image like the horns of a ram (that is, one end is sinistral and the other is dextral). This leads us to think that the chirality is ‘inherited’ from chamber to chamber during shells growth, but that an occasional ‘mistake’ can cause a reversal in the direction of translation of the spiral relative to the anterior / posterior axis. Following from this, we hypothesize that in some species there may be a significant ‘error rate’ in the left /right placing of the deuteroconch on the proloculus with respect to the genetically inherited coiling direction. That could explain why very few species coil exclusively left or right. If the ‘error rate’ is correlated to an environmental variable like temperature it could also explain some of the ecophenotypic correlations that have been observed [68]. The case may be analogous to some flatfish where asymmetry is heritable but with a significant error rate that may be stochastic or related to environmental variables in early growth [62].

Coiling direction correlates with reproductive barriers in at least some animal species. For instance, the spermatophores of some gastropods have chiral tails with a lock-and-key element to successful reproduction that favours out-crossing [63]. In such cases a genetic mutation affecting coiling direction can set up an instant reproductive barrier [72] and contribute to speciation [73]. However foraminifera are isogamous (individuals producing large numbers of gametes of similar morphology that are not ‘male’ and ‘female’). Gamete fusion has apparently not been observed in the laboratory but it remains possible that there may be some subcellular or molecular chiral aspect to successful gamete fusion that correlates with the macroscopic coiling direction.

The best-known instance of coiling direction variability in the planktonic foraminifera is the case of *Neogloboquadrina* (the most closely related genus to *Pulleniatina*). In the 1950s it was found that left-coiling intervals of *N*. *pachyderma* correspond to climatically cold phases in Quaternary sediment cores of the North Atlantic, and hence the coiling direction was used as a climate proxy [74]. It has subsequently become clear that left- and right- dominated populations are genetically and morphologically distinct, with the polar species (*N*. *pachyderma* sensu stricto) being sinistrally dominant and the temperate species (now recognized as a closely related species, *N*. *incompta*) being dextrally dominant [75]. Hence down-core reversals in the observed coiling ratio are related to the climatically-driven movement of the sub-polar front across much of the central North Atlantic, in which *N*. *pachyderma* and *N*. *incompta* are sampled alternately in cold and warm phases. To complicate matters further it seems that both *N*. *pachyderma* and *N*. *incompta* populations have occasional (<3%) individuals that coil the other way [75]. Recent laboratory observation has confirmed the asexual reproduction of a single sinistral specimen of *N*. *pachyderma* (or possibly *N*. *incompta*) which produced offspring that coil both ways, confirming that there must be an ecophenotypic component to chirality, at least in this species [76].

Various early studies found a correlation between dextral percentage and temperature in some other species such as *Globorotalia truncatulinoides* and *G*. *menardii*. It has been a constant temptation for workers to extend a simple left/right = cold/warm model across the group as a whole but this is clearly unwarranted. For instance, coiling reversals in the *Paragloborotalia continuosa*–*Neogloboqudarina acostaensis*–*Pulleniatina* group have not been convincingly linked to climatic fluctuations [77]. Instead it appears that periodic coiling reversals in this group and some others occur across much of the planet, including throughout the tropical Indo-Pacific, indicating that at any given time, most or all of the global population has a similar coiling bias. Hence the explanation discussed above of *N*. *pachyderma* / *N*. *incompta* apparent coiling reversals being related to oceanic frontal movements cannot be generalized to all such cases, and a different explanation is required.

Apart from *N*. *pachyderma*, few studies have addressed the link between coiling direction and genetics in modern planktonic foraminifera. Darling et al. [78] found that both left and right coiling specimens of *Globigerina bulloides* belonged to a single apparent genetic type (Type IId). They suggested that this might indicate a deeper level of cryptic diversity than has so far been revealed by standard techniques at the time (SSU rRNA gene sequencing). Another illuminating case study is *Globorotalia truncatulinoides* which has been subdivided into four genetic types, all of which are exclusively sinistral except for Type II which has dextral representatives. Moreover Type II-R (right) and Type II-L (left) have distinct ecological (depth, seasonal) and biogeographic distributions [61] and so apparently represent additional cryptic diversity that has yet to be fully defined.

### A model for coiling ratio variation in foraminifera

Based on the discussion above, we suggest that coiling direction in foraminifera must be a heritable character in at least some lineages, although the genetic basis for it is not currently known. Mutation of the hypothetical coiling gene would cause an individual and its descendants to spontaneously coil the opposite way, although, as discussed above, there may also be a significant error rate during development such that some individuals spontaneously coil the ‘wrong’ way with respect to their genes. Different species may have different error rates, which would go a long way to explaining the different patterns seen across the clade. Error rates may be higher in a stressed environment or in asexual reproduction which has so far been confirmed in just one species [76].

It is difficult to imagine an adaptive advantage for a free-floating protist to coil one way or another. It is possible that a hypothetical gene that defines chirality is linked at the molecular / chromosome level to another gene that has a selective advantage, but that is currently unknown. Overall in planktonic foraminifera there are approximately equal numbers of dextrally and sinistrally biased lineages, so there is certainly no overall preferred coiling direction. It is simpler therefore to imagine that it is a neutral character. Coiling shifts might then be envisaged as a single gene mutating and evolving by random drift in one global interbreeding population, at times becoming fixed or nearly fixed (as in the current situation in *Pulleniatina*, which is exclusively dextral). But this leads to a dilemma in that the vast global population size means that it would be difficult for random drift to effect a significant change in the ratio even on geological time scales because the time to fixation of a neutral gene is a function of effective population size [79].

A potential solution to this dilemma is if the species as a whole is not a single interbreeding population, as the genetic evidence strongly suggests [11]. Genetically isolated cryptic populations might be continually appearing and replacing one another within an evolutionary species-lineage. Populations might start small and therefore could develop a separate coiling preference through a random founder effect or by drift in small groups. If such a population had a selective advantage within the overall niche it could then rapidly replace its competitors and thereby fix a neutral gene or character within the species-lineage as a whole.

We interpret our isotope and size data to indicate that at some times at least, sinistral and dextral forms belonged to distinct sub-populations that had subtly different habitat preferences. There is an analogy to be drawn with the different genotypes of *Pulleniatina* in the modern ocean which, as discussed above, show different geographic preferences that correlate with temperature [11]. An even closer analogy is with the modern genotypes of *Globorotalia truncatulinoides* which vary in both habitat and coiling preference [7, 61]. If modern *G*. *truncatuloides* Type II, which alone among the genotypes of that species has dextral representatives [61], happened to evolve some advantage and replace all the other genotypes within the species-lineage, a global coiling shift would occur.

Because the isotopic differences we observed at different time intervals are not consistent, our data do not support the idea that there are just two groups of *Pulleniatina*, left and right dominant respectively, that have maintained consistent differences throughout the record. It is therefore likely that there were multiple cryptic populations at any one time, any of which may or may not have shown significant differences in their coiling preferences. The crash in dextral abundance at 2.035 Ma is especially interesting because it likely occurs across the entire Indo-Pacific [46, 48, 49]. We suggest that it indicates the widespread extinction of a population that would have remained entirely cryptic were it not for its preference for dextral coiling. More single-specimen isotope analysis at different sites would test this possibility.

Shipboard investigations during Expedition 363 [54–56] confirmed that the ancestral lineage to *Pulleniatina*, which involves the morphospecies *Paragloborotalia continuosa* and *Neogloboquadrina acostaensis*, shows a similar coiling reversal history back to at least 9.8 Ma, so the process has been going on for a long time. However a successful explanatory model must also account for the various patterns seen in other lineages of planktonic foraminifera including, for instance, those in which there are only occasional coiling shifts. These might simply be less prone to producing genetically isolated populations. Lineages which show long-term gradual drift in coiling direction might have more cryptic genotypes. For cases that are apparently randomly coiled throughout their existence and those with only slight biases it may be that the hypothetical ‘coiling gene’ is expressed only weakly during development, or not at all, so that randomness or ecophenotypic variation predominates at the moment the organism begins to build its shell. An interesting example of this is the modern species *Trilobatus sacculifer* which, unusually, has just one known genotype [80] but for which the coiling direction, as has been measured in hundreds of individuals, is consistent with being entirely random [81].

A problem with the model proposed here, however, is that it is difficult to relate the coiling history in *Pulleniatina* to the supposedly long existence of the modern Type I, Type IIa and Type IIb genotypes, all of which are exclusively dextral. If the Type I and Type II genotypes diverged around three million years ago as has been suggested from molecular clock estimates [11] then that must have happened in the Indo-Pacific because the genus was absent from the Atlantic at that time. In all the Indo-Pacific records there are periods of very high sinistral dominance between 1.8 and 2.0 Ma (e.g., see Figs 5 and 7). This would indicate either that the modern dextral genotypes were present at very low abundance relative to other sinistral genotypes that are now extinct, or that they independently changed their coiling preferences from sinistral to dextral over time. The genotypes would also have had to experience a degree of parallel evolution because there has been significant morphological change in the lineage as a whole since 3 Ma. These things are possible, but another explanation worth considering is that the divergences between the modern genotypes are more recent than has been proposed. The high variability in inferred substitution rate of ribosomal genes across the planktonic foraminifer clade as a whole is well known [8–10] and might compromise attempts to determine divergence times from genetic data.

### Ecological speciation and genotype sweeps

At the beginning of this paper we defined our terms by adopting Barraclough’s species definition [3]. An alternative would have been to base the species definition on reproductive isolation (as is conventionally referred to as the ‘biological species concept’), in which case we would have referred to the distinct genotypes that exist in the modern ocean as probable cryptic species within a larger evolving ‘species-cluster’ (or similar term) called *Pulleniatina obliquiloculata*. This semantic decision has a ‘Necker cube’ like quality that led to much fruitful discussion while this paper was in review. The advantage of taking the Barraclough definition as a standpoint is that it allows a clearer focus on the long-term evolving lineage as an entity to be explained. The fossil record shows ‘evolutionary lineages’ that span millions to tens of millions of years [2]. Are these lineages merely the sum result of an ever-changing patchwork of closely related but morphologically identical or barely distinguishable species, or are they, as Barraclough’s definition requires ‘independently evolving’, that is, something cohesive in and of themselves with meaningful start and end points in time? Morphometric studies of fossil lineages suggest the latter [e.g., 19].

The discovery of cryptic genetic diversity in many types of organisms is challenging long-established theories of evolution and speciation and is currently a major area of debate [3, 17]. Evidence from planktonic foraminifera offers unique insights into this wider debate precisely because of the extended time dimension [2]. A fundamental question is therefore exposed: why has the high level of cryptic diversity in the group not ultimately led to greater divergence of form and a much larger number of biological species than are actually observed?

The classic neo-Darwinian description of natural selection focuses on the differential survival and reproduction of individuals within sexually interbreeding populations. The environment provides a limit or carrying capacity to a species that prevents endless geometric growth in numbers, hence individuals compete for resources to achieve reproductive success. Genetic mutations that are advantageous to survival and reproduction spread through subsequent generations until they become fixed, while deleterious genes are eliminated. This process means that species are constantly adapting to their physical and biological environment and can potentially persist indefinitely, although extinction will occur if populations become ill-adapted to their environment for some reason. Of course it is generally acknowledged that reproductive isolation may not be absolute, and that hybridism can occur, as can horizontal gene transfer even between distantly related species [3].

In this classical model, speciation is generally caused by circumstances that isolate populations for long enough that they accumulate distinct genetic identities and, for whatever reason, are subsequently unable to interbreed. The resulting populations can then develop distinct ecological strategies and adaptations allowing them to diverge in form and function indefinitely, generating biodiversity. In this view of evolution, the ’cohesive force’ that binds a species in the long term is sexual reproduction, so much so that the very definition of a species has often been taken to be that group of individuals that has the potential to successfully reproduce to give fertile offspring [82]. Sexual reproduction has been proposed as a general explanation for why life is strongly granular in genes and morphology and does not blend through an endless variety of intermediate forms. This is the essence of the ’biological species concept’, a key part of the so-called ‘modern synthesis’ of twentieth century evolutionary theory (see discussion in [3, 83]).

Evolution does not, however, require sex. Asexual organisms such as bacteria also engage in a struggle for survival and reproduction, and they too adapt to their environment through natural selection. Despite that, there is not an endless diversity of form or genetic variety among bacteria and other asexual organisms; they too cluster into distinct groups based on shared inherited characteristics that, importantly, evolve through time [84]. This has led to the concept of the ’ecotype’ in asexual evolution, in which the ’force of cohesion’ is not the reproductive connectivity of a species but a shared ecological strategy that places its members in competition with each other for resources within a set environmental carrying capacity. If one genotype has a slight advantage over others in the ecotype it will replace its competitors in a ‘selective sweep’ [85–87]. Ecotypes are described as "populations of organisms occupying the same ecological niche, whose divergence is purged recurrently by natural selection" (p. 457 in [87]).

Some modern species definitions such as the one preferred here seek to be inclusive of asexual organisms and acknowledge that species do not have to be defined by sexual inter-breeding and, consequently, emphasize that speciation is not a necessary result of the appearance of reproductive isolation [3, 83]. If a sexually interbreeding population were to divide in two with the onset of a reproductive barrier for whatever reason, it does not necessarily follow that there are suddenly two ecological niches and the environmental carrying capacity for organisms is somehow doubled. Instead, there is a growing appreciation that ecological divergence is the key factor in speciation, and divergent selection the driving force. This is the theory of ‘ecological speciation’ [88–90]. In this model, reproductively isolated populations will compete for dominance within the same niche unless and until new ecological opportunities in the physical or biological environment encourage long-term divergence.

Similar concepts are increasingly being applied to planktonic foraminiferal species [8, 91–93]. In line with these studies, we suggest that planktonic foraminifer species are analogous to bacterial ecotypes in that they are primarily constrained by a shared ecology rather than reproductive inter-breeding. Within the species, sexual isolation and partial ecological divergence may be quite common, accounting for the fact that most modern species comprise multiple separate genotypes that sometimes have distinct coiling characteristics. Despite usually developing somewhat different environmental preferences and geographic or depth distributions, these genotypes remain locked in global competition for shared resources. It follows from this that ecological speciation is much less common than reproductive isolation. In the history of *Pulleniatina*, clear and lasting ecological speciation has happened just once, when the *spectabilis* lineage diverged from the main *obliquiloculata* lineage and eventually became a distinctly different form, hence meeting the definition of species we have adopted. This ecological cohesiveness explains why the densely sampled fossil record shows discrete and coherent independently evolving lineages that often persist for millions to tens of millions of years before splitting or becoming extinct [92].

To what extent lasting evolutionary change in biological species occurs by gene fixation in reproductively isolated groups or genotype sweeps within the broader ecotype likely varies markedly from one type of organism to another. Some of the classic evolutionary case studies involving large complex macro-organisms with elaborate courtship rituals that live in relatively small, geographically restricted populations may correspond closely to Mayr’s ‘biological species concept’ [82, 83]. The vastly abundant bacteria and other asexual microorganisms lie at the other end of the spectrum. In our view, the sexual planktonic foraminifera lie somewhere in between, probably because their large, intermixed populations have acted to limit the likelihood of ecological speciation [91–93]. Lineages undoubtedly evolve substantially over time, as did both the *spectabilis* and *obliquiloculata* lineages. Evolution could occur both by the classical neo-Darwinian mechanism involving the spread and fixation of advantageous genes in sexually isolated populations *and* a process more analogous to bacterial ‘sweeps’ in which isolated genotypes replace some or all of their competitors within the niche. They would be very difficult to distinguish in the fossil record. Both mechanisms would result in new genetic configurations becoming fixed as the ecologically confined and defined lineage evolves [93].

## Supporting information

S1 Fig Photographs of all analysed specimens from Windows W1-W5. Specimen numbers are keyed to size and isotopic data in S8 Table. All photographs to same scale.
(PDF)

S1 Table Age model for IODP Site U1486. Age model is constructed by linear interpolation through the mid-points of magnetic reversals documented in Ref [52] and converted to CCSF scale. The Lower Cobb Mountain reversal is omitted.
(XLSX)

S2 Table Data collected in this study from IODP Site U1486. Samples are ordered by depth within each segment of the inter-hole splice. Depths below sea floor on the CSF-A scale are converted to composite depths on the CCSF scale according to offsets given in the affine table in Ref [52]. Ages are calculated within the age-model tie points indicated and as given in S1 Table. Sample weights and *Pulleniatina* coiling counts of 50 specimens are reported. In the intensive study windows indicated, samples were split and the absolute number of sinistral and dextral specimens recorded; these data were substituted for earlier counts, and those counts discounted, in these windows only.
(XLSX)

S3 Table Shipboard coiling data from IODP Hole 1486B from Ref [52]. Sample depths are converted from the CSF-A scale to the CCSF scale according to offsets given in the affine table in Ref [52]. Ages are calculated within the age-model tie points indicated and as given in S1 Table. Magnetic reversals within the hole are shown for reference although the tie-points used for the age model are from the multi-hole splice and not necessarily Hole U1486B.
(XLSX)

S4 Table Combined coiling data from IODP Site U1486. Data from S2 and S3 Tables are combined and ordered according to age, with small samples (<20 *Pulleniatina* omitted). 95% Confidence Intervals on the proportion of dextral *Pulleniatina* are calculated according to the Modified Wald method with fixed upper and lower limits at 100% and 0.15% to facilitate graph plotting.
(XLSX)

S5 Table Age model for IODP Hole U1483A. Age model is constructed by linear interpolation through the mid-points of magnetic reversals documented in Ref. [51] on the CSF-A scale. The Brunhes / Matuyama reversal is omitted.
(XLSX)

S6 Table Combined shipboard and new *Pulleniatina* data from IODP Hole U1483A. Data from this study include sample weights. Shipboard data are from Ref [51] with depths re-calculated from the affine table in Ref [51]. Age model by linear interpolation from paleomagnetic reversals in Ref [51] as given in S5 Table.
(XLSX)

S7 Table *Pulleniatina* absolute abundances and flux calculations from Windows W1-W5. (XLSX)

S8 Table Sinistral and dextral *Pulleniatina* size and stable isotope data from Windows W1-W5. Pairwise statistical tests for differences between sinistral and dextral populations for size, δ^13^C and δ^18^O are included.
(XLSX)
