# Supplementary material for: Coiling directions in the planktonic foraminifer Pulleniatina: A complex eco-evolutionary dynamic spanning millions of years
Source: PLoS One. 2021 Apr 13;16(4):e0249113. doi: 10.1371/journal.pone.0249113 (PMC8043407; doi:10.1371/journal.pone.0249113)
Supplement: S1 Fig — Specimen numbers are keyed to size and isotopic data in S8 Table. All photographs to same scale. (PDF) [file pone.0249113.s001.pdf]

S1 Fig. Photographs of analysed specimens

Sample	Coiling	Number	Page
U1483A/9H/4, 88-90 cm	Sinistral specimens	1-25	2
	Sinistral specimens	26-50	3
	Dextral specimens	51-75	4
	Dextral specimens	76-100	5
U1486D/7H/2, 57-59 cm	Sinistral specimens	1-25	6
	Sinistral specimens	26-50	7
	Dextral specimens	51-75	8
	Dextral specimens	76-100	9
U1486D/10H/3, 107-109 cm	Sinistral specimens	1-25	10
	Sinistral specimens	26-50	11
	Sinistral specimens	51-52	12
	Dextral specimens	53-77	13
	Dextral specimens	78-102	14
	Dextral specimens	103-106	15
U1483A/15H/6, 58-60 cm	Sinistral specimens	1-25	16
	Sinistral specimens	26-50	17
	Dextral specimens	51-75	18
	Dextral specimens	76-100	19
U1483A/17H/4, 57-59 cm	Sinistral specimens	1-25	20
	Sinistral specimens	26-50	21
	Sinistral specimens	51-75	22
	Sinistral specimens	76-7	23
	Dextral specimens	78-95	24

Sample U1483A/9H/4, 88-90 cm: sinistral specimens

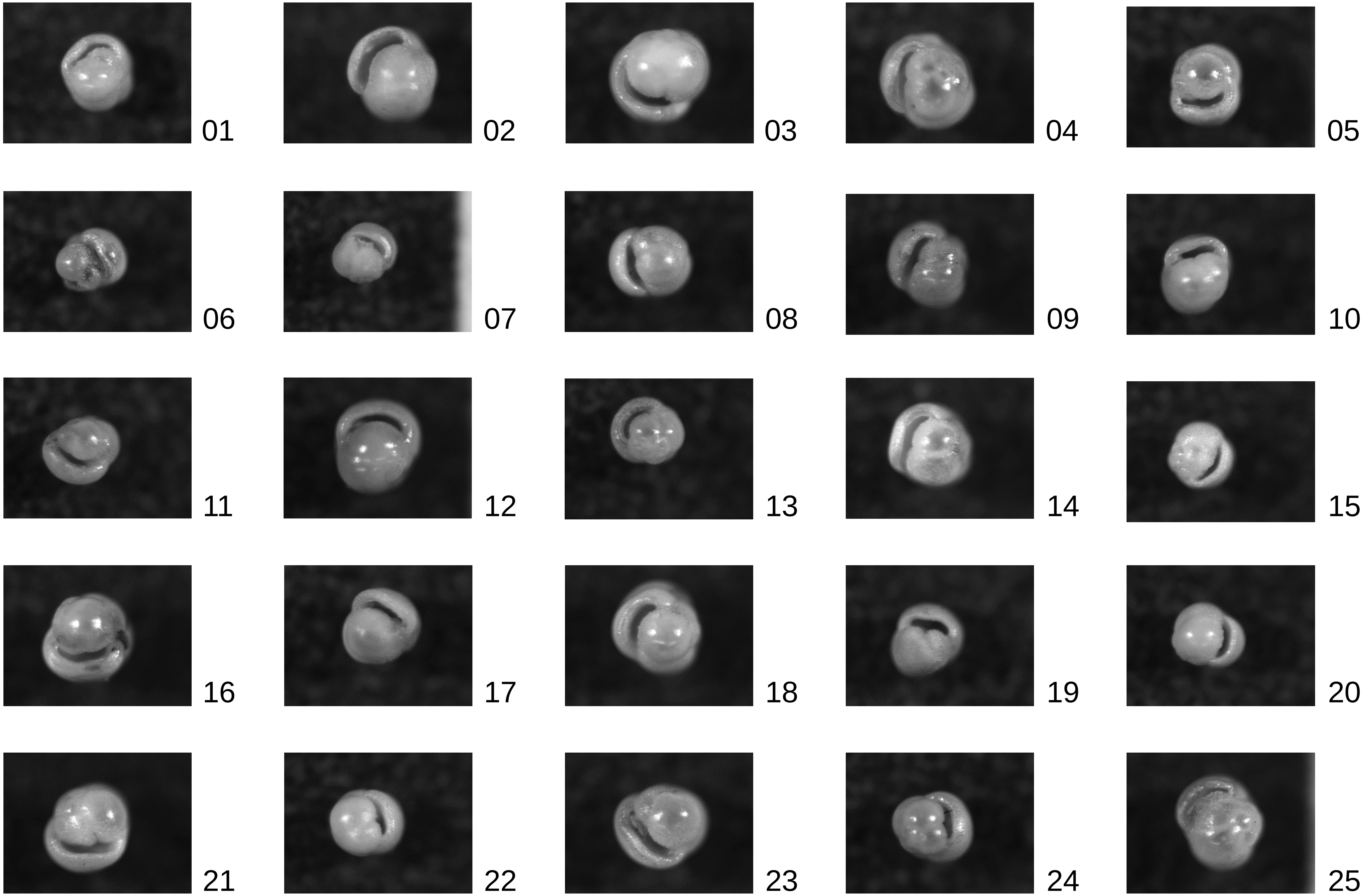

Sample U1483A/9H/4, 88-90 cm: sinistral specimens

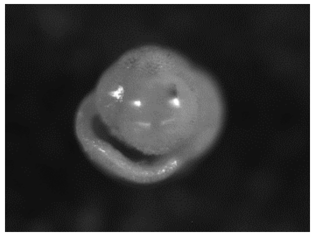
26

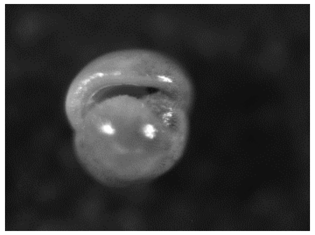
27

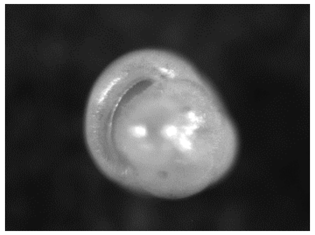
28

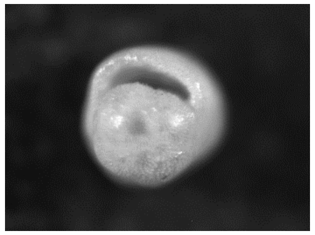
29

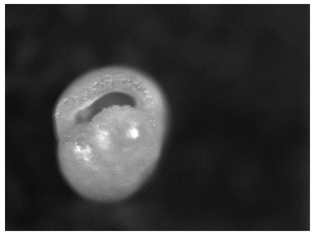
30

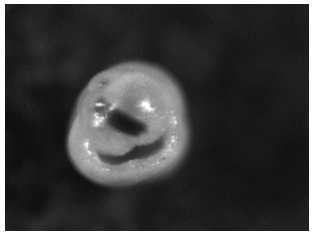
31

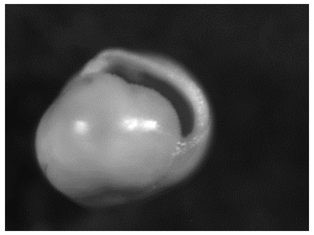
32

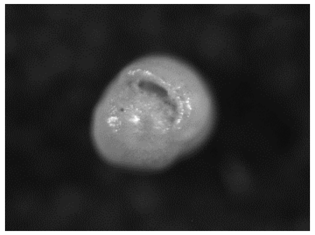
33

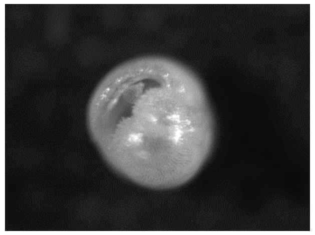
34

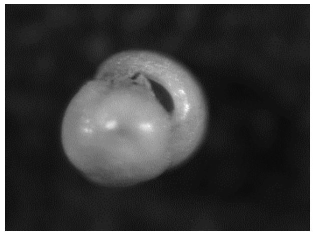
35

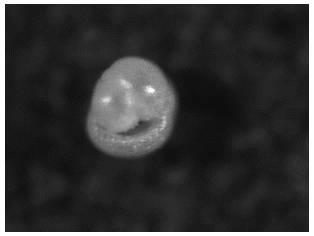
36

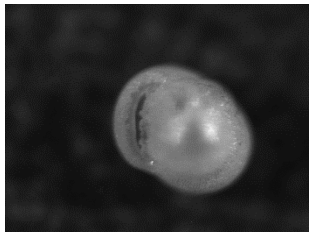
37

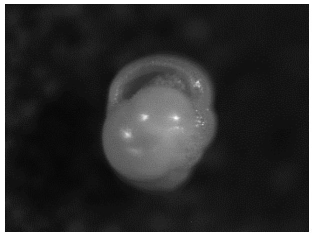
38

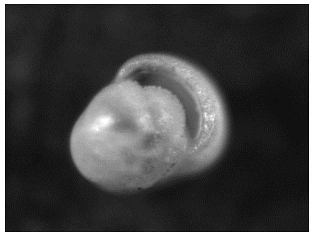
39

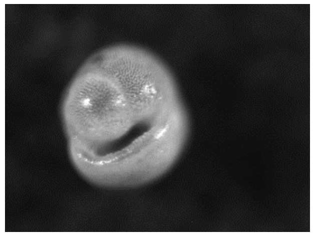
40

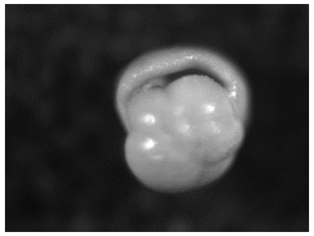
41

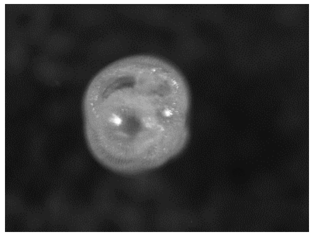
42

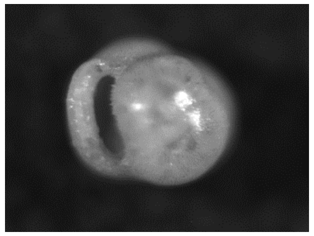
43

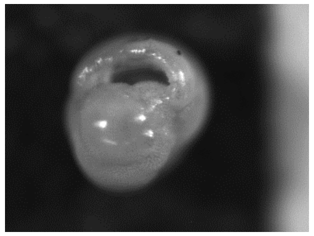
44

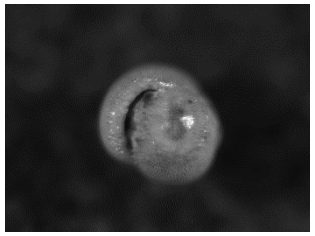
45

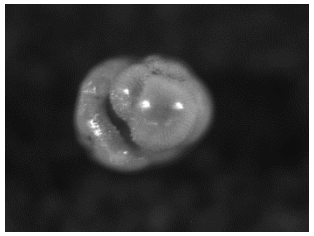
46

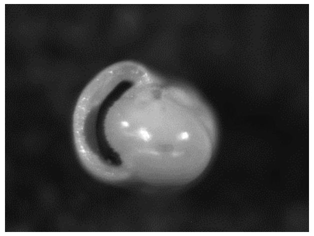
47

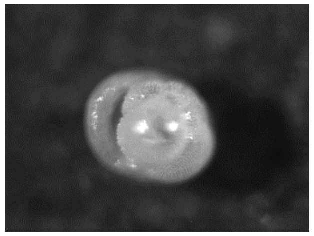
48

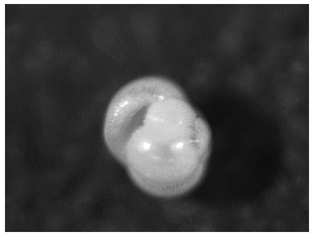
49

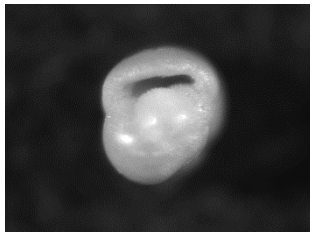
50

Sample U1483A/9H/4, 88-90 cm: dextral specimens

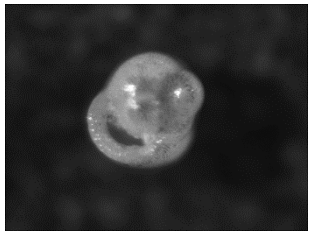
51
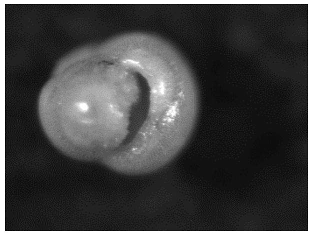
52
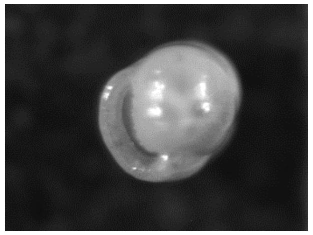
53
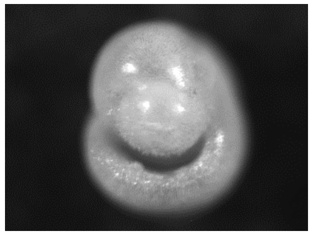
54
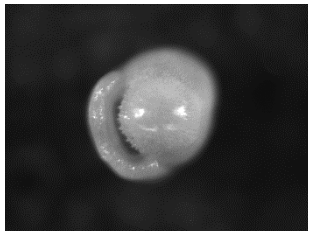
55
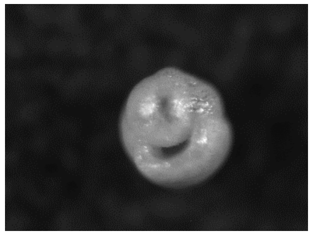
56
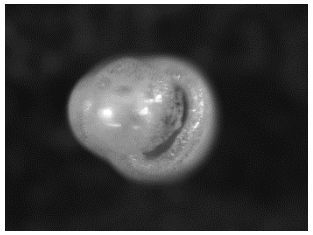
57
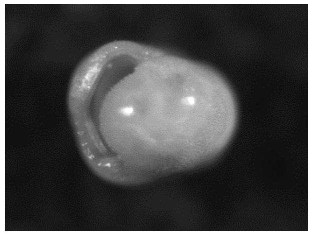
58
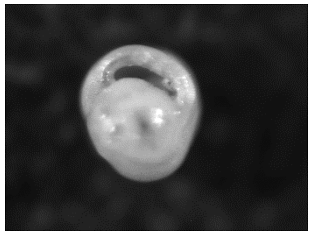
59
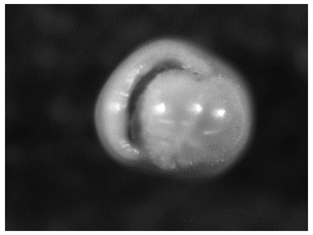
60
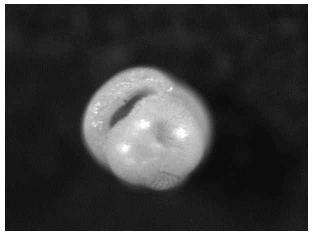
61
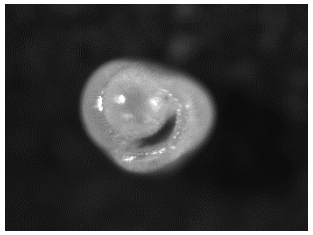
62
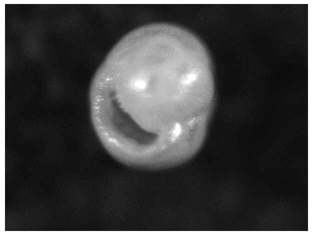
63
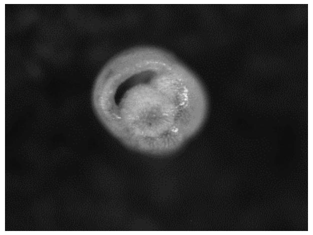
64
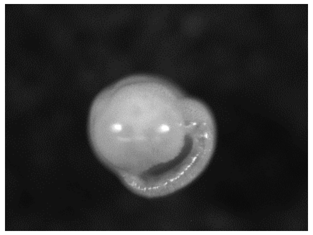
65
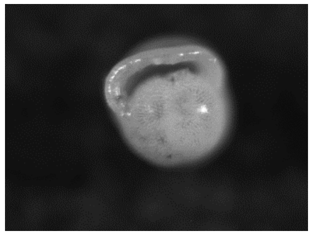
66
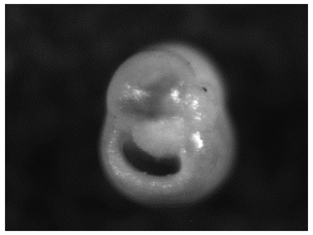
67
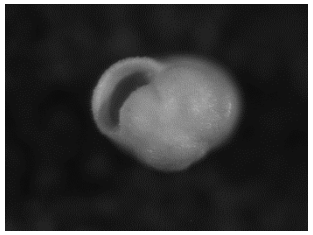
68
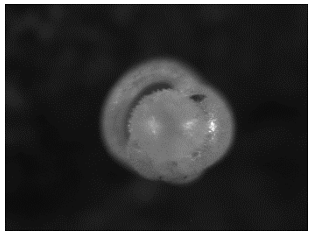
69
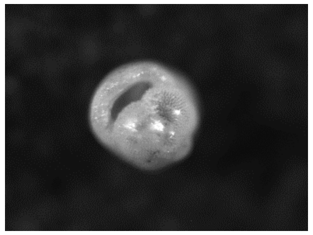
70
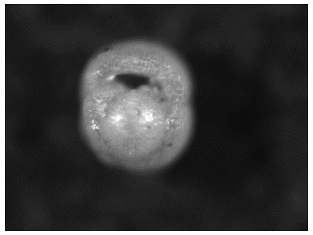
71
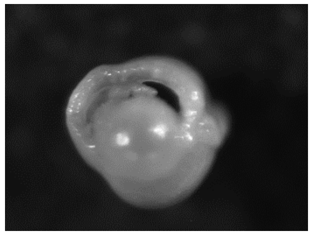
72
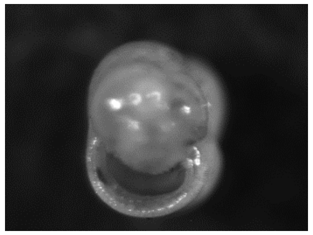
73
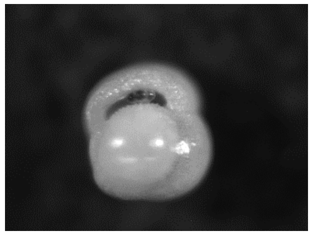
74
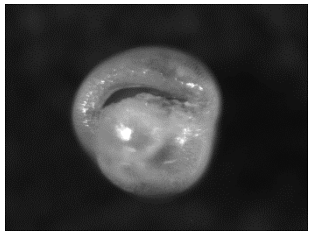
75

Sample U1483A/9H/4, 88-90 cm: dextral specimens

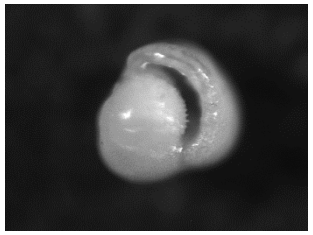
76
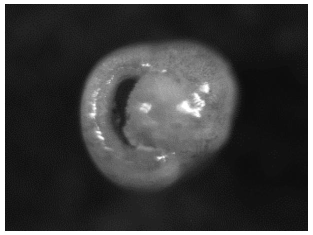
77
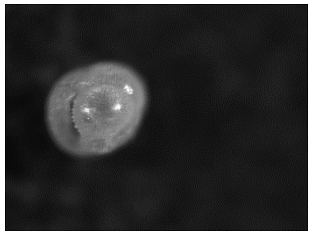
78
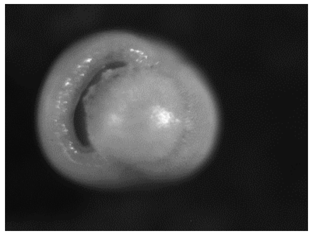
79
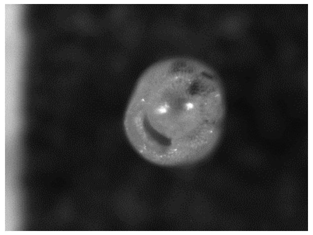
80
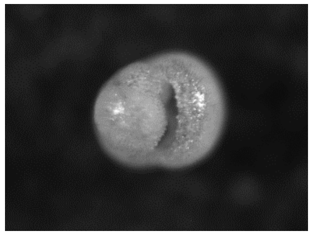
81
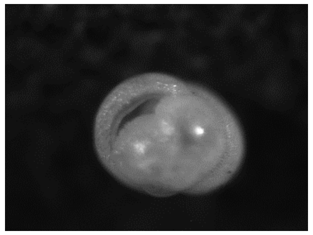
82
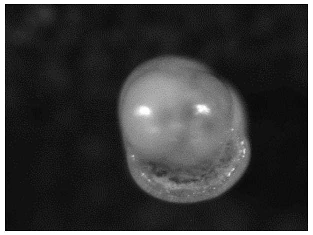
83
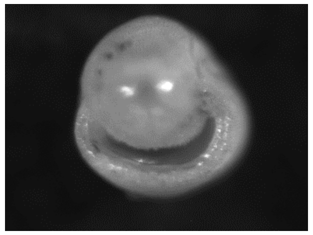
84
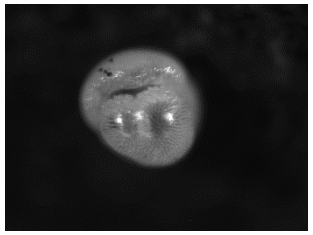
85
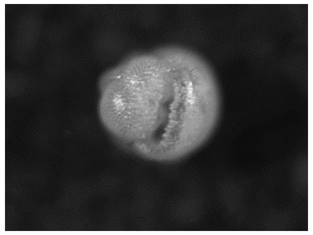
86
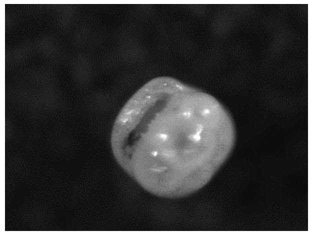
87
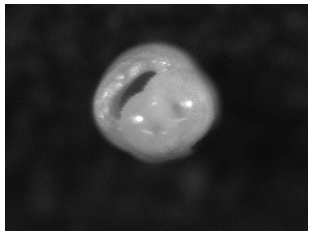
88
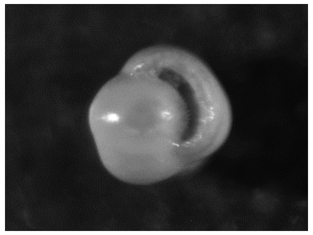
89
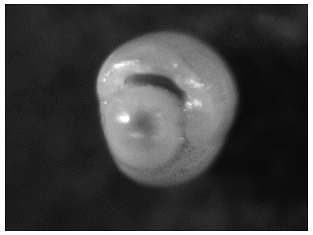
90
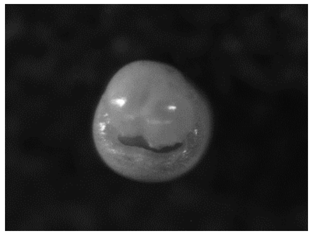
91
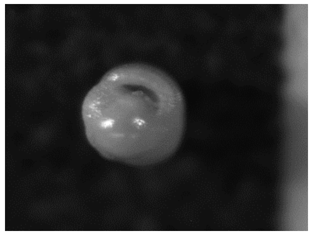
92
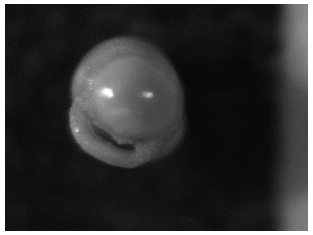
93
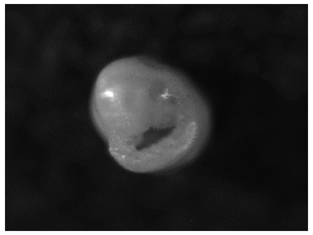
94
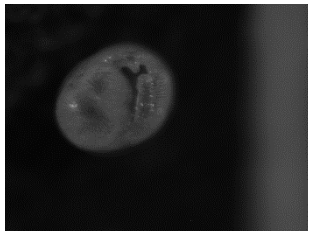
95
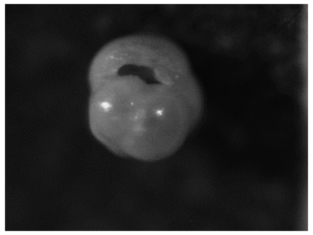
96
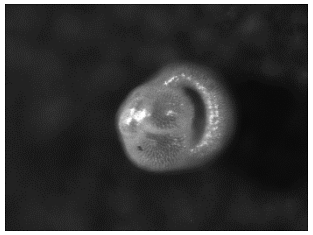
97
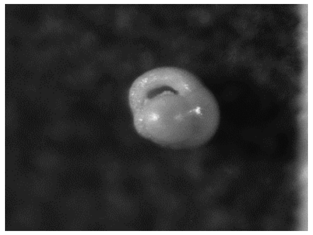
98
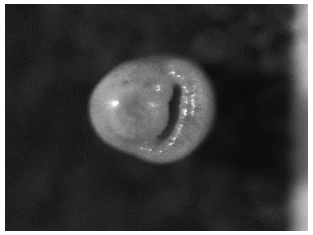
99
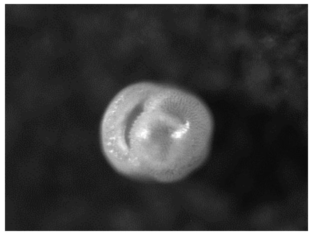
100

Sample U1486D/7H/2, 57-59 cm: sinistral specimens

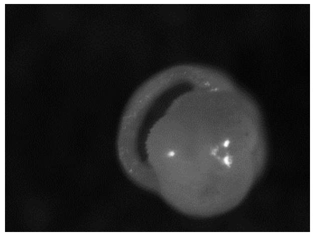
01

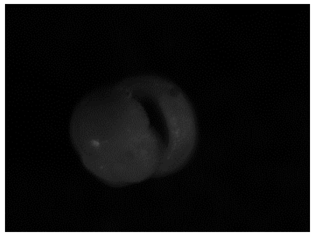
02

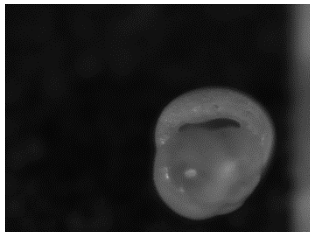
03

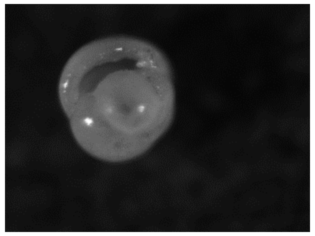
04

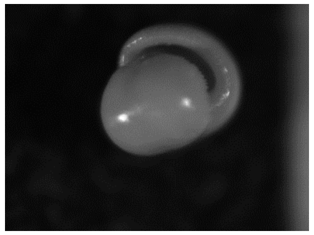
05

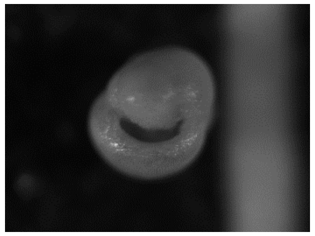
06

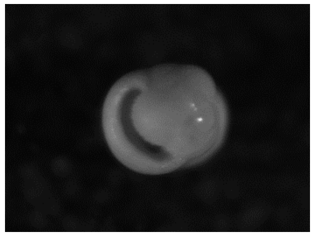
07

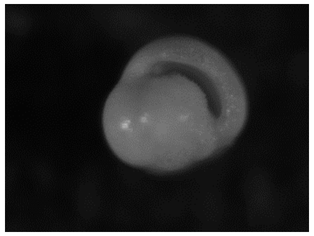
08

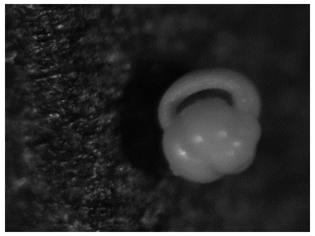
09

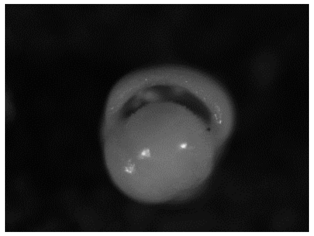
10

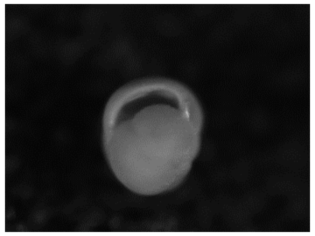
11

deleted
= *G. inflata*
12

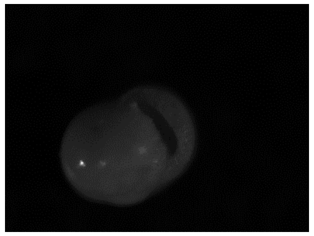
13

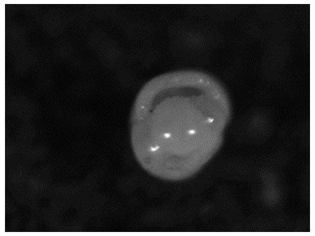
14

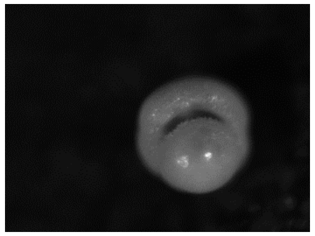
15

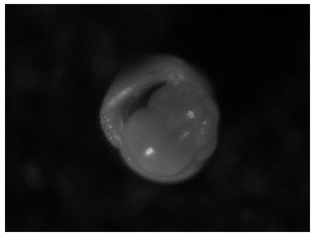
16

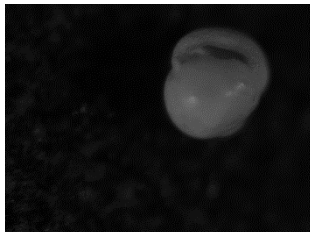
17

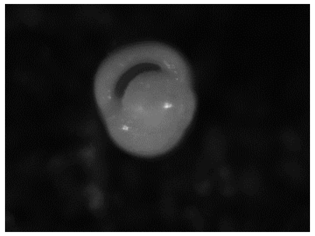
18

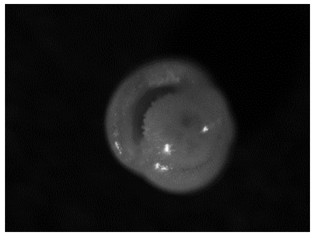
19

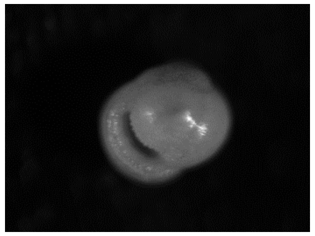
20

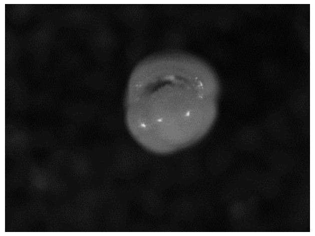
21

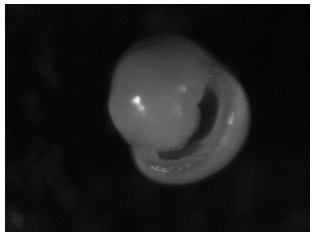
22

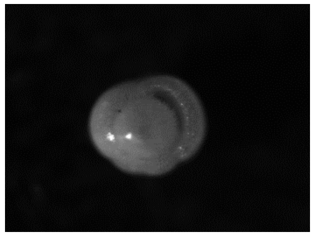
23

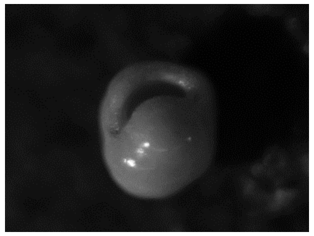
24

deleted
= *G. inflata*
25

Sample U1486D/7H/2, 57-59 cm: sinistral specimens

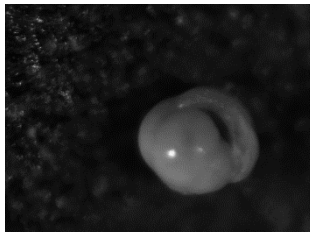
26

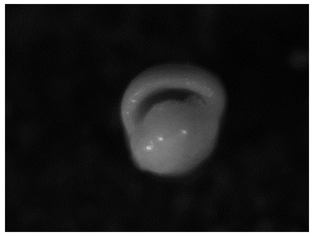
27

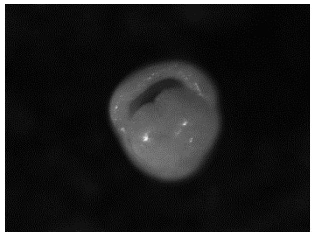
28

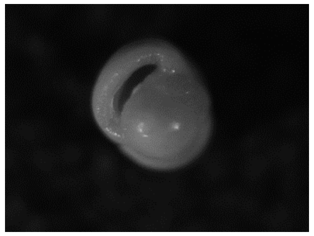
29

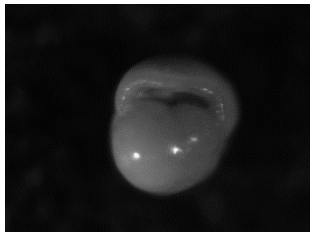
30

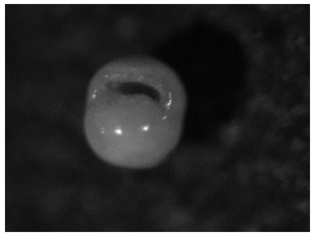
31

deleted
= *G. inflata*
32

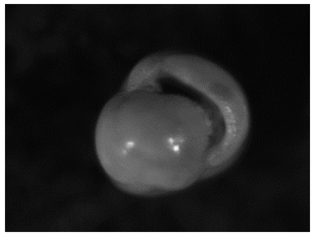
33

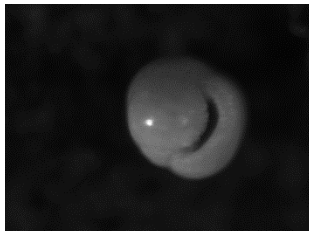
34

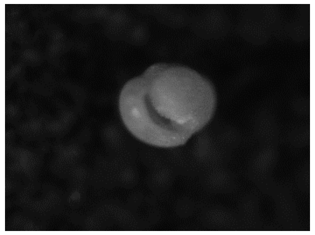
35

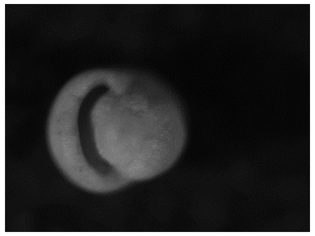
36

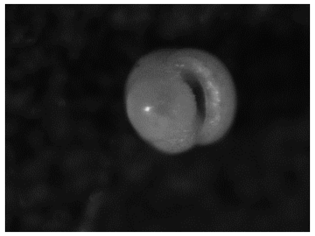
37

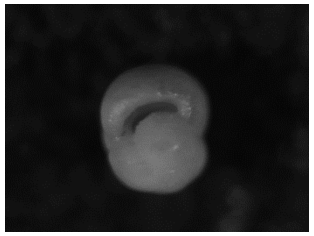
38

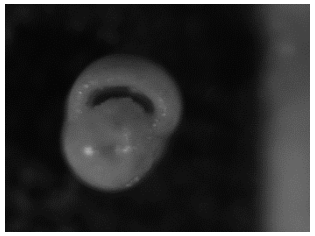
39

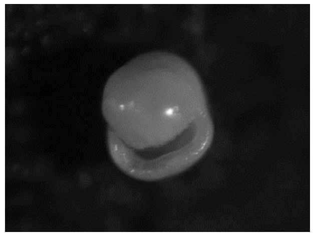
40

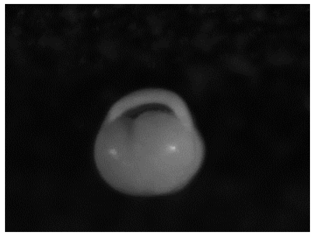
41

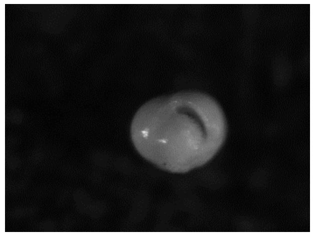
42

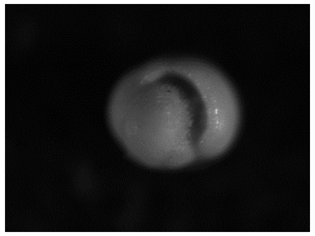
43

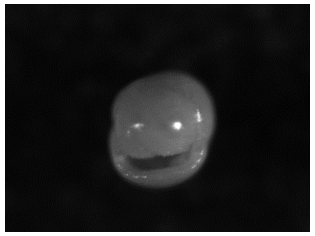
44

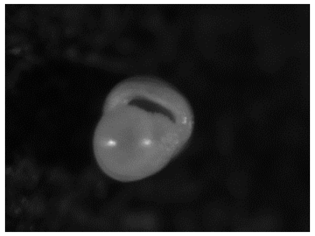
45

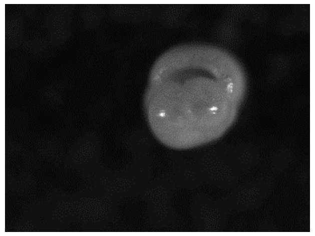
46

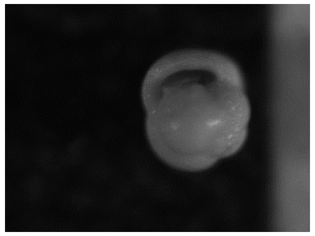
47

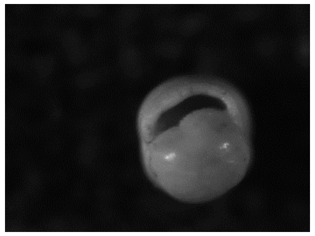
48

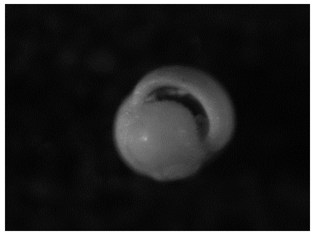
49

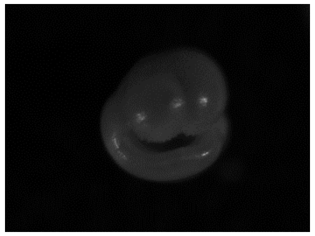
50

Sample U1486D/7H/2, 57-59 cm: dextral specimens

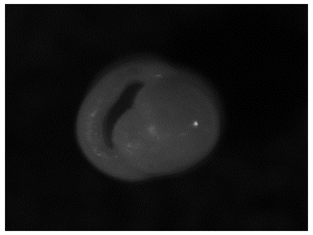 51

deleted
= *G. inflata* 52

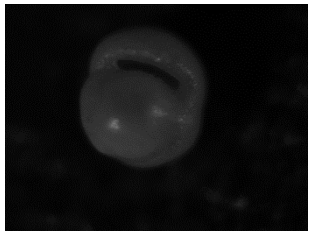 53

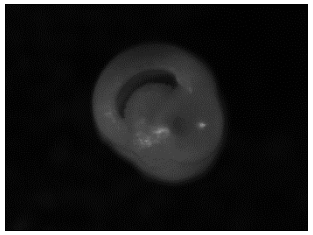 54

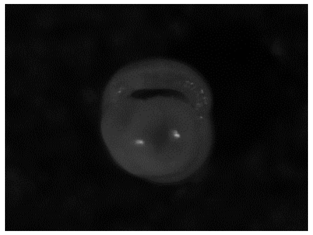 55

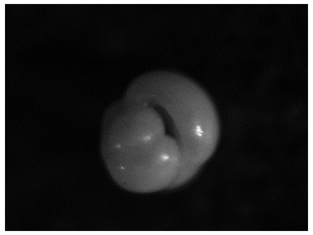 56

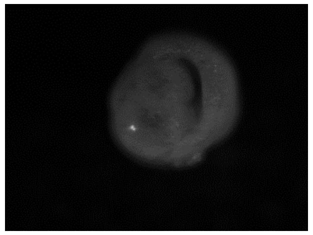 57

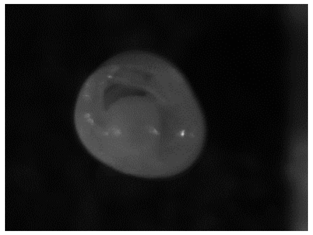 58

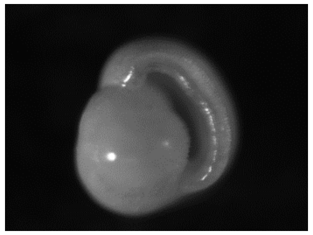 59

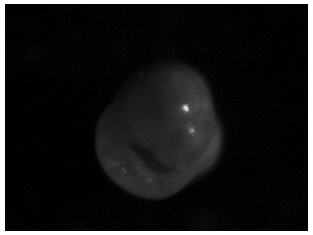 60

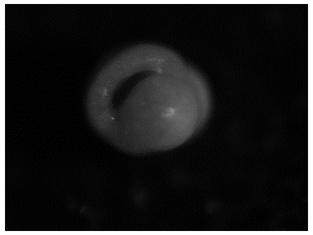 61

deleted
= *G. inflata* 62

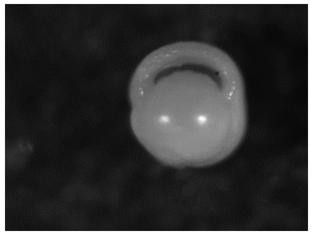 63

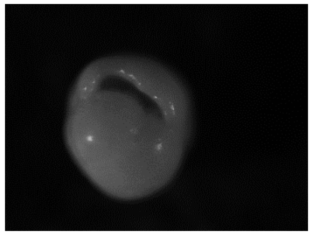 64

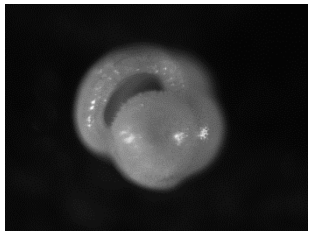 65

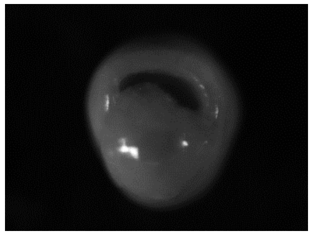 66

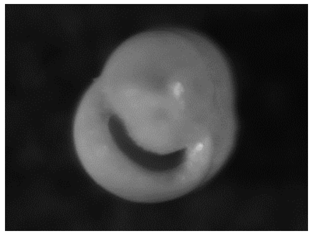 67

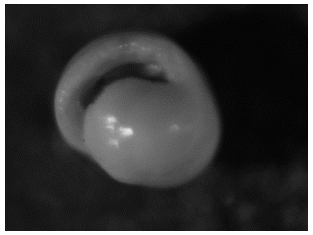 68

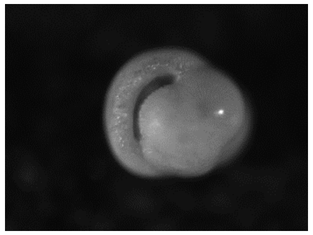 69

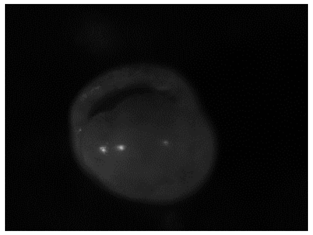 70

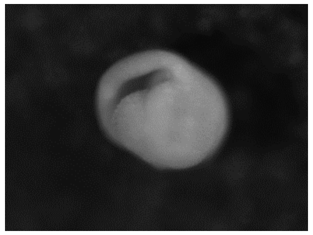 71

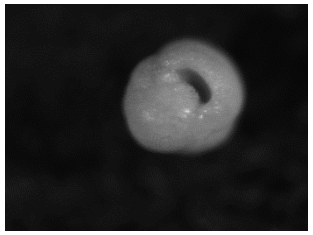 72

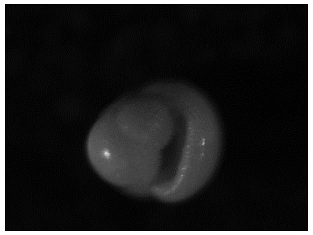 73

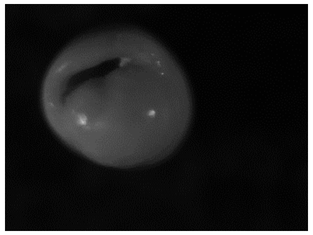 74

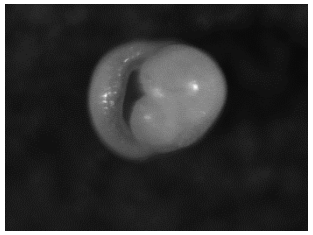 75

Sample U1486D/7H/2, 57-59 cm: dextral specimens

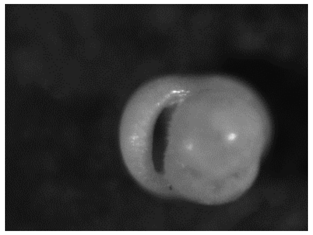
76

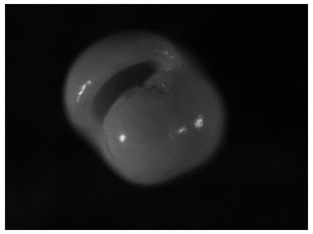
77

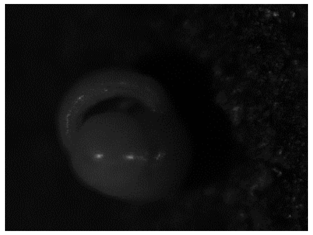
78

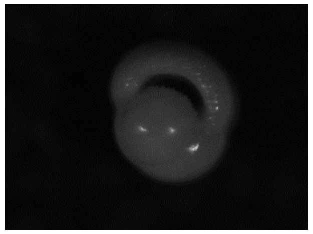
79

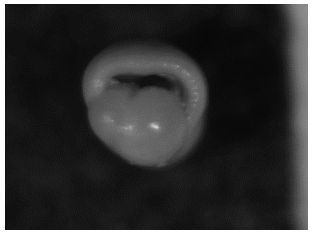
80

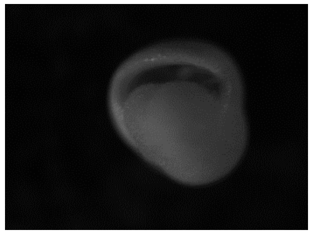
81

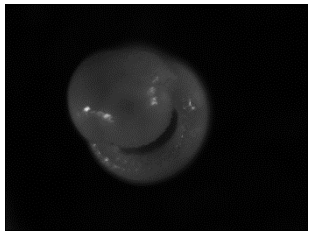
82

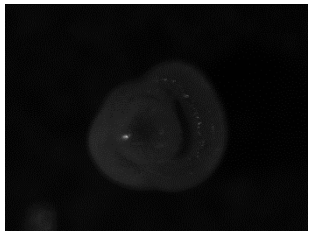
83

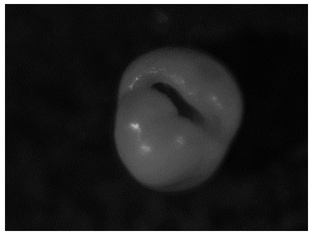
84

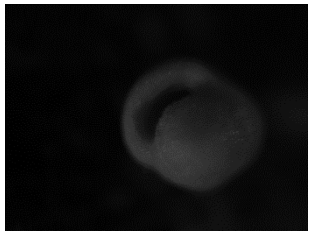
85

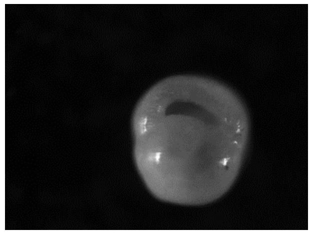
86

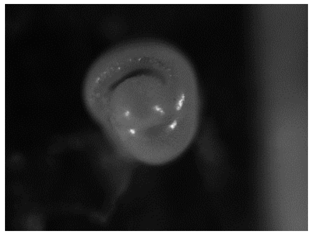
87

deleted
= *G. inflata*
88

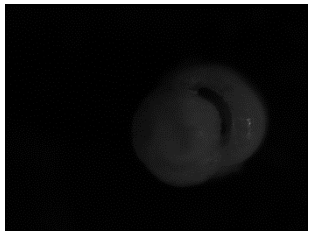
89

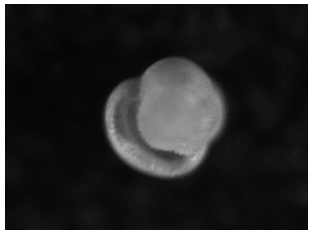
90

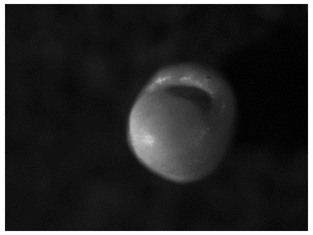
91

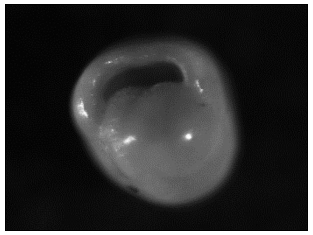
92

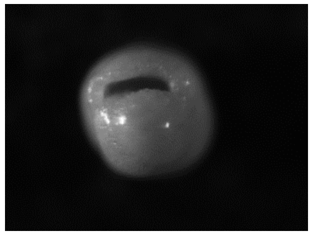
93

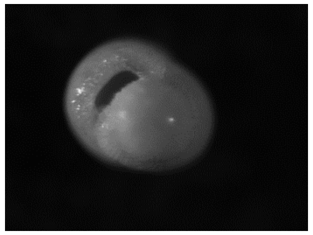
94

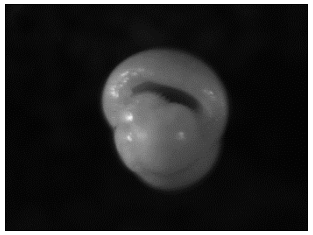
95

deleted
= *G. inflata*
96

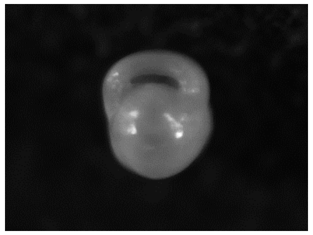
97

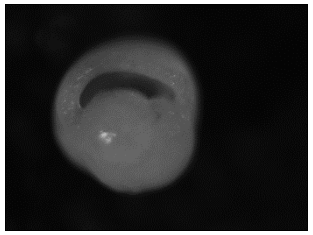
98

deleted
= *G. inflata*
99

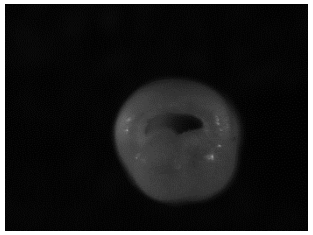
100

Sample U1486D/10H/3, 107-109 cm: sinistral specimens

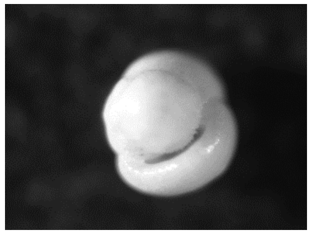
01

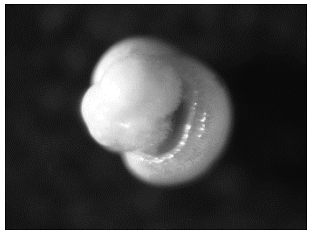
02

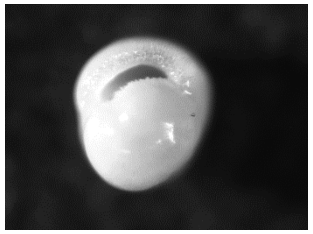
03

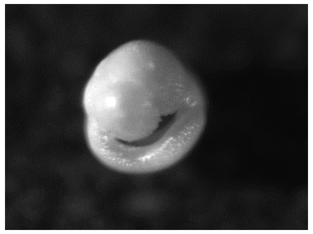
04

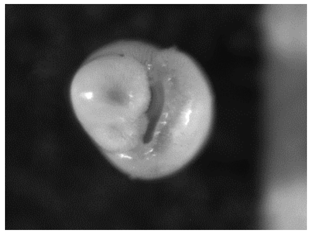
05

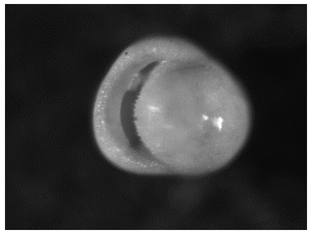
06

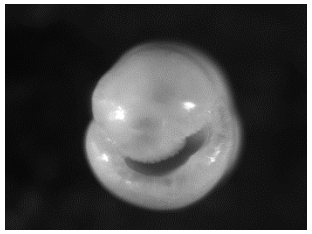
07

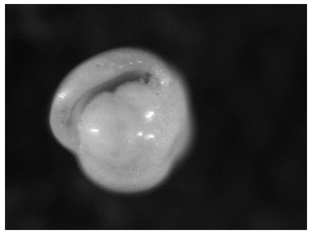
08

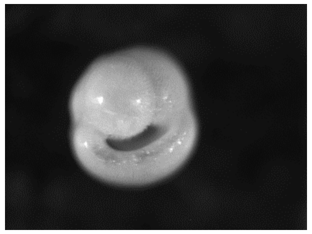
09

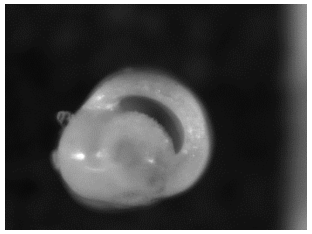
10

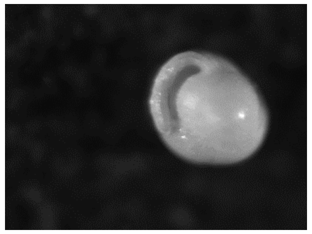
11

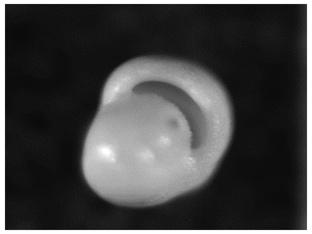
12

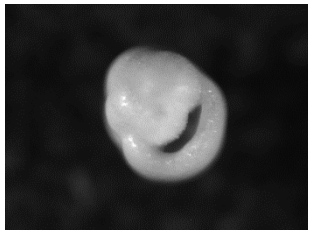
13

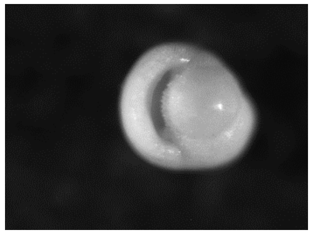
14

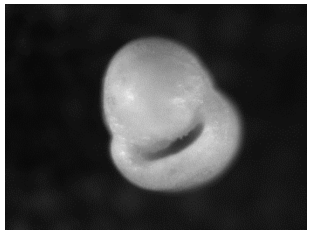
15

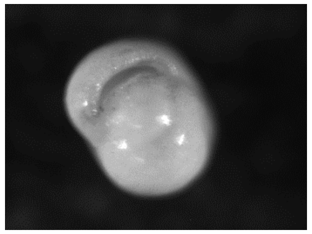
16

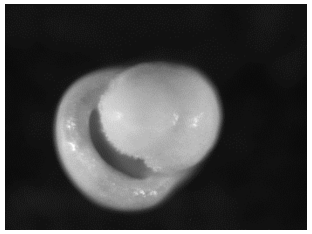
17

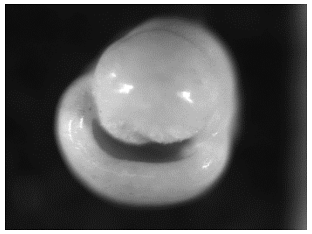
18

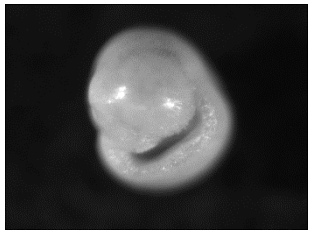
19

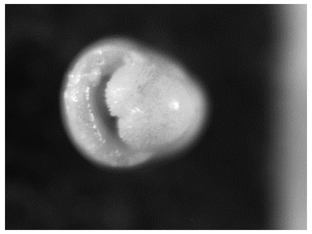
20

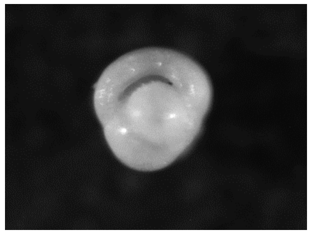
21

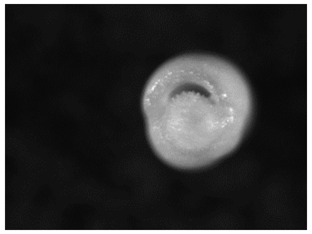
22

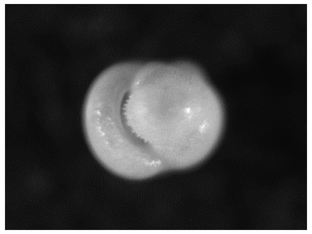
23

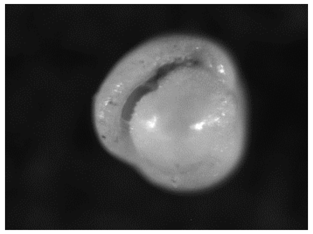
24

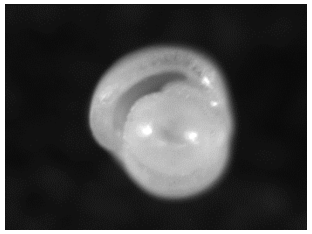
25

Sample U1486D/10H/3, 107-109 cm: sinistral specimens

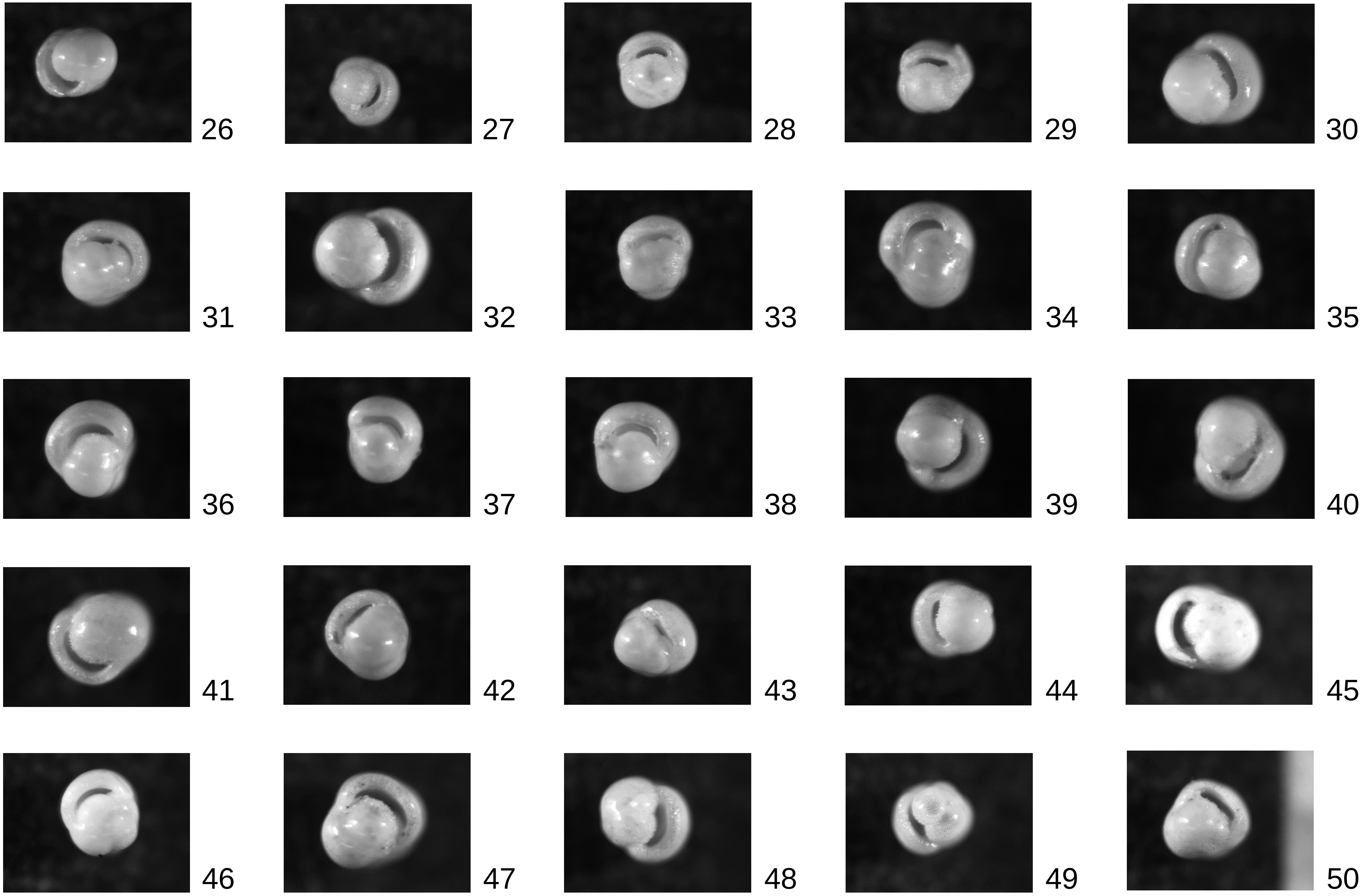

Sample U1486D/10H/3, 107-109 cm: sinistral specimens

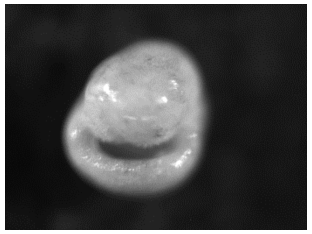
51

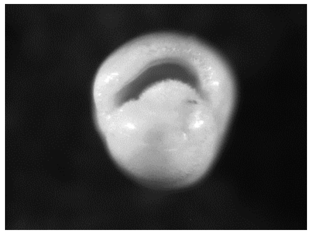
52

Sample U1486D/10H/3, 107-109 cm: dextral specimens

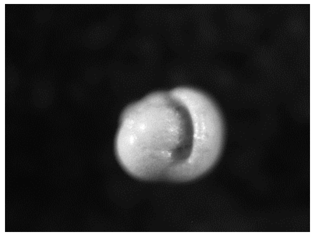
53
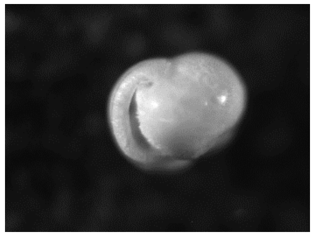
54
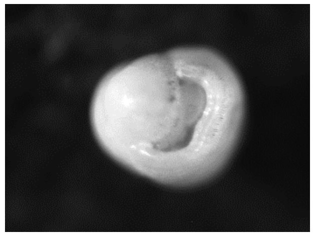
55
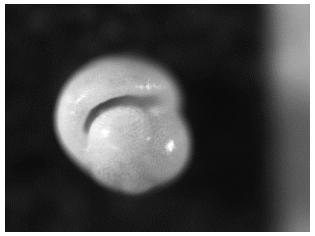
56
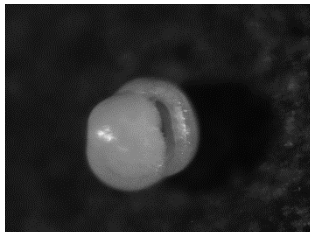
57
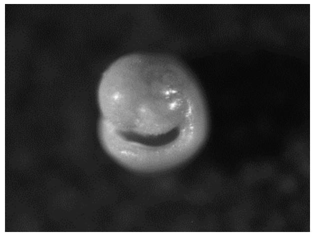
58
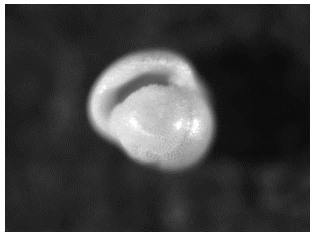
59
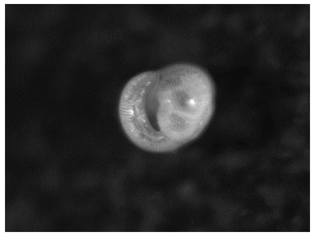
60
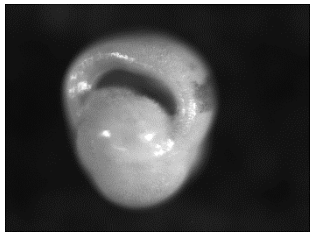
61
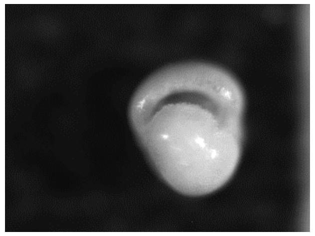
62
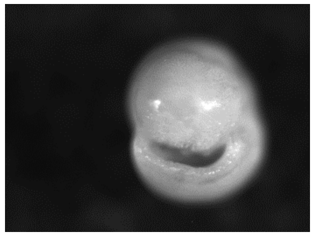
63
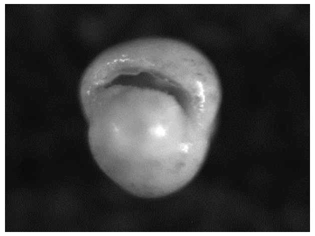
64
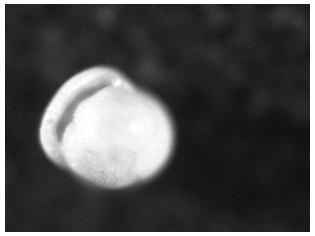
65
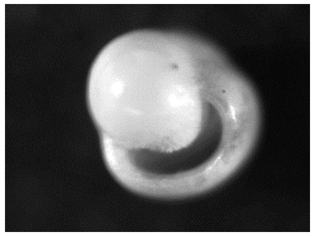
66
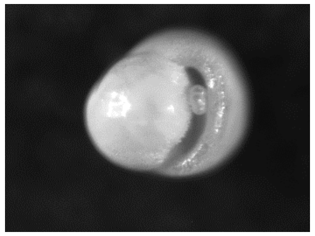
67
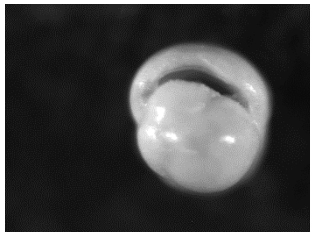
68
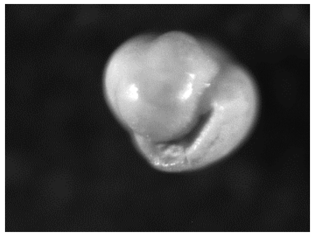
69
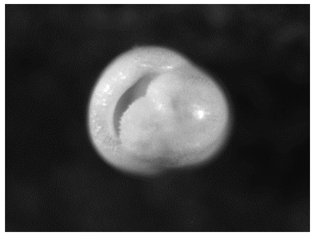
70
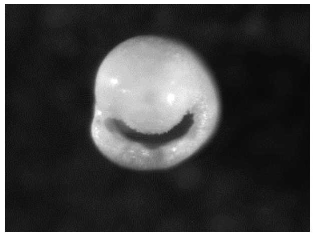
71
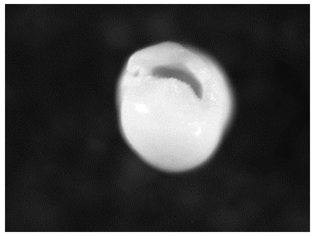
72
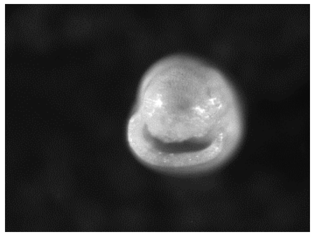
73
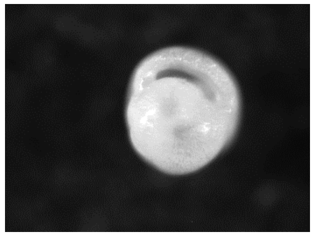
74
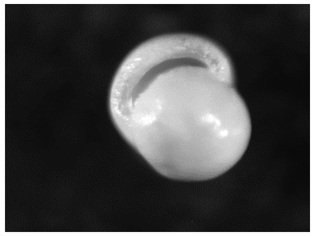
75
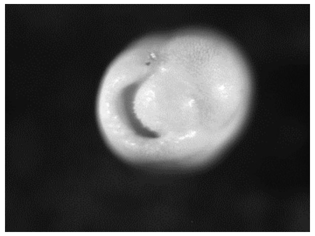
76
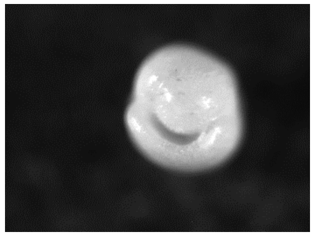
77

Sample U1486D/10H/3, 107-109 cm: dextral specimens

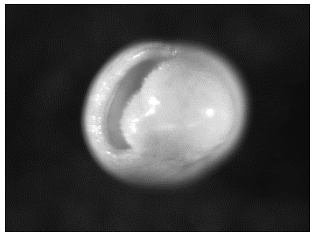
78
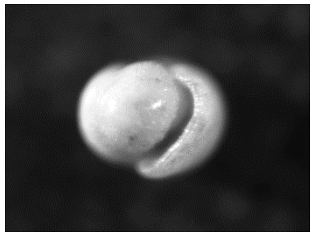
79
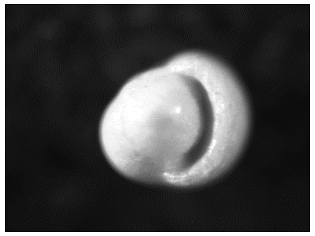
80
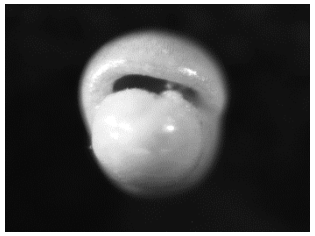
81
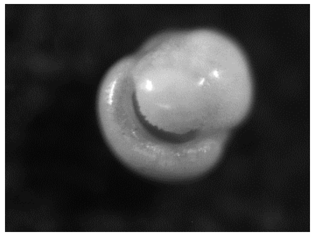
82
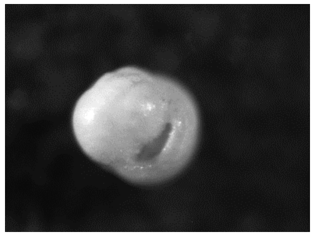
83
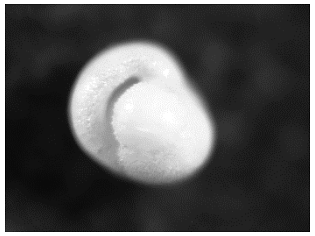
84
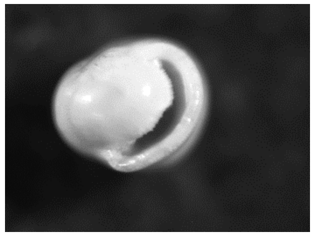
85
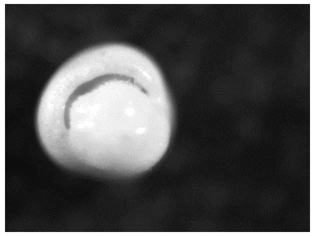
86
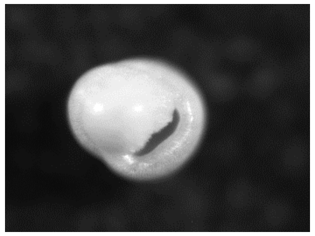
87
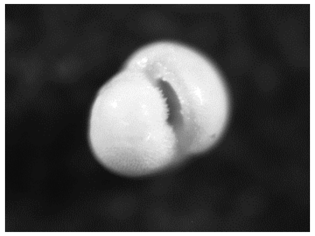
88
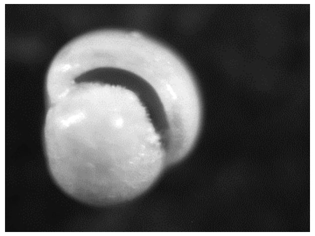
89
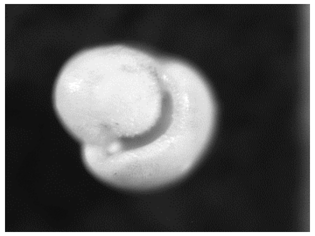
90
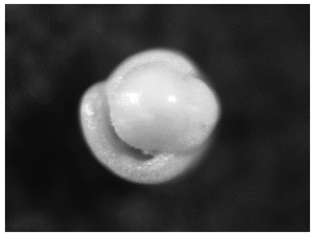
91
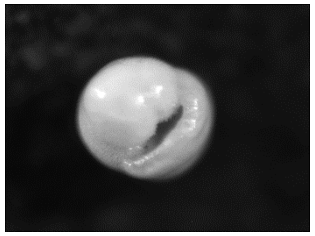
92
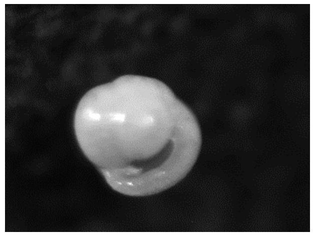
93
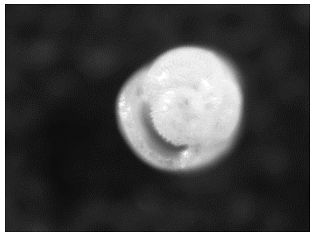
94
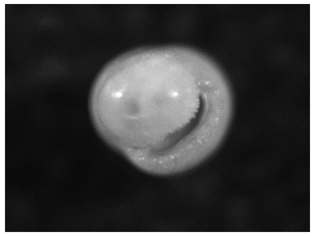
95
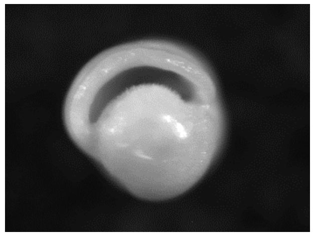
96
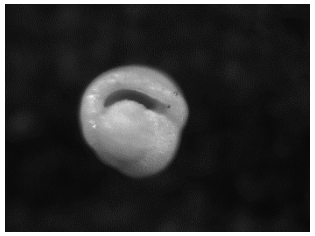
97
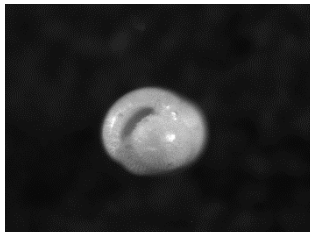
98
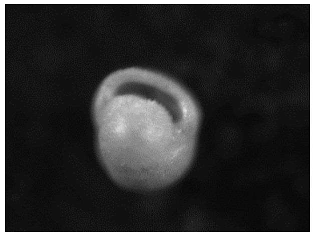
99
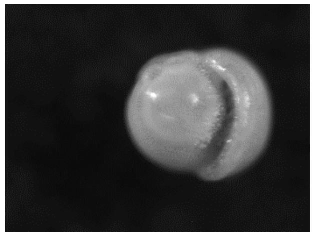
100
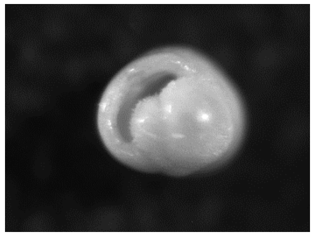
101
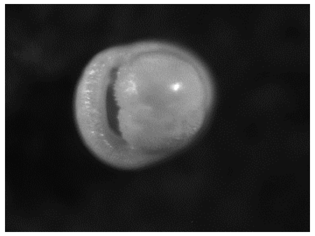
102

Sample U1486D/10H/3, 107-109 cm: dextral specimens

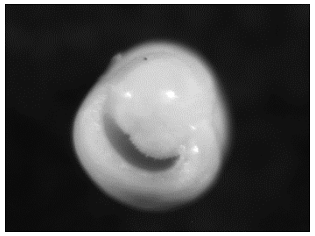
103

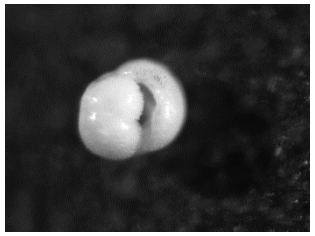
104

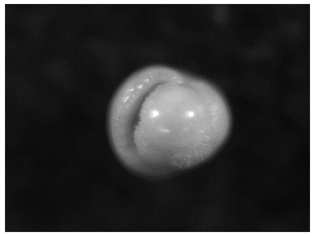
105

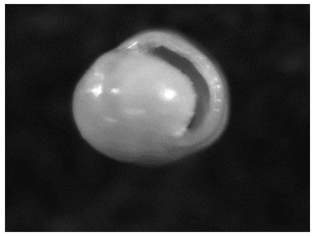
106

Sample U1483A/15H/6, 58-60 cm: sinistral specimens

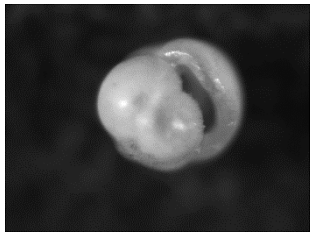
01

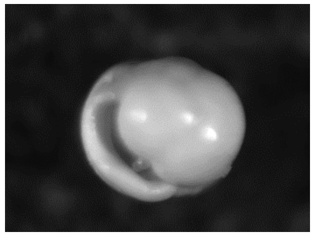
02

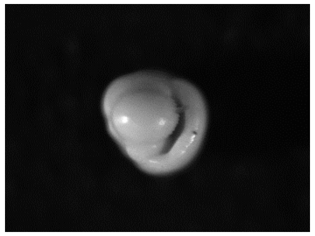
03

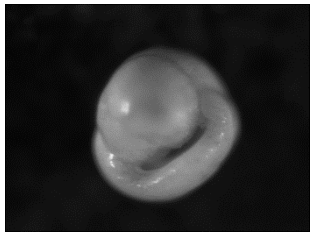
04

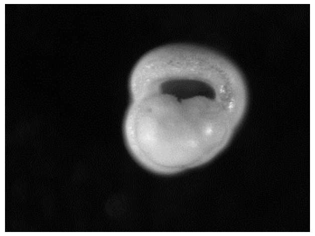
05

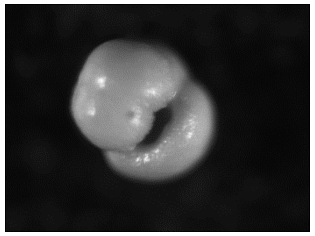
06

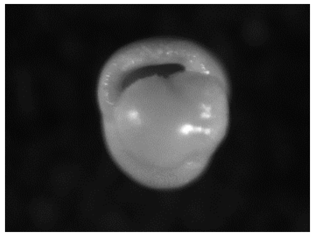
07

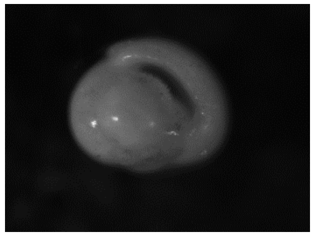
08

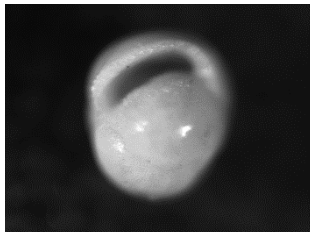
09

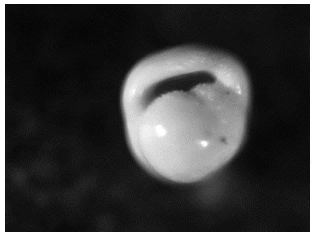
10

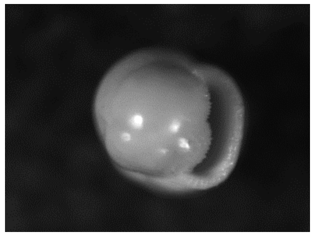
11

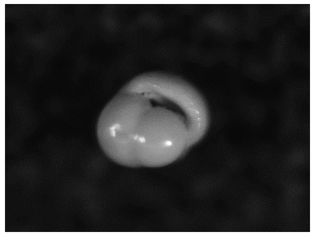
12

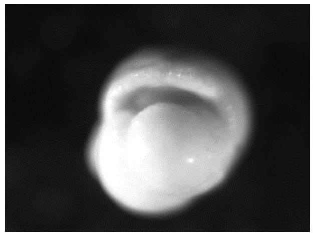
13

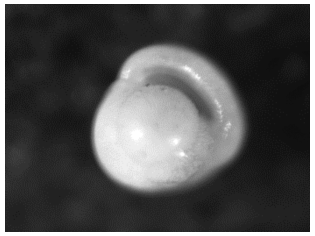
14

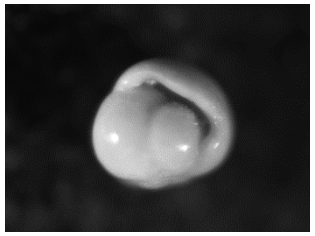
15

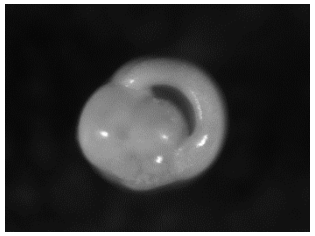
16

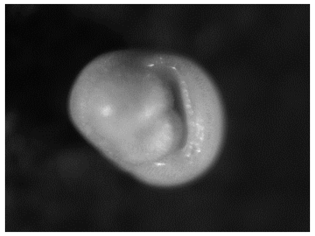
17

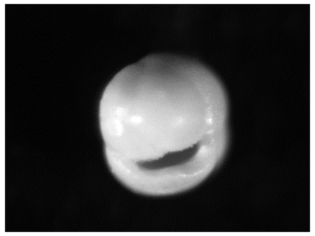
18

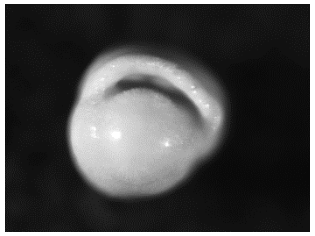
19

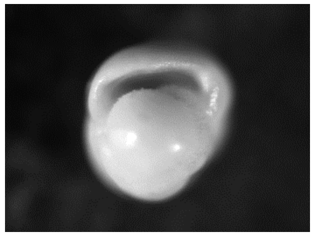
20

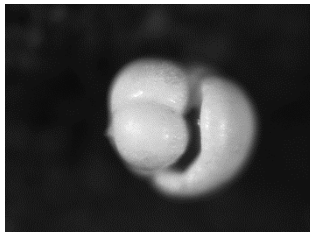
21

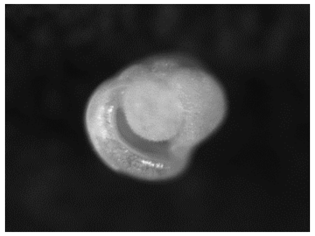
22

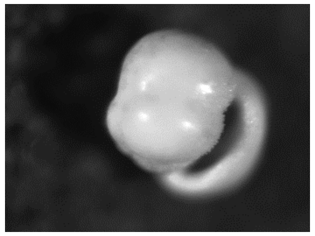
23

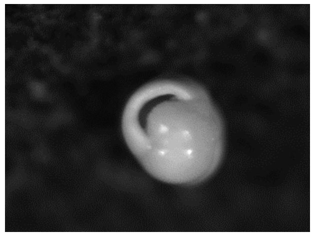
24

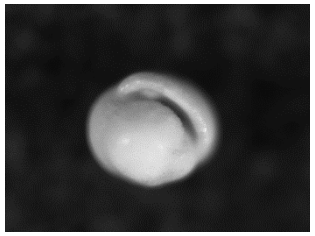
25

Sample U1483A/15H/6, 58-60 cm: sinistral specimens

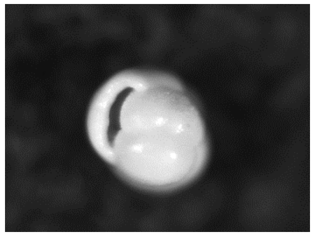
26
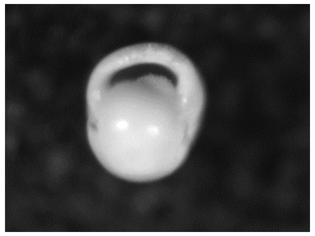
27
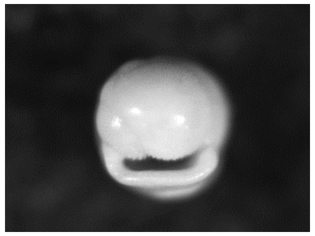
28
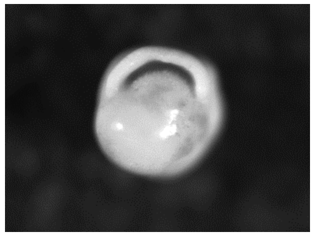
29
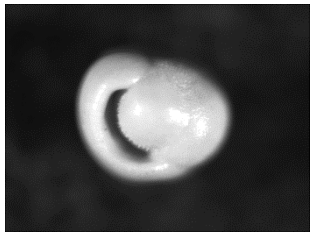
30
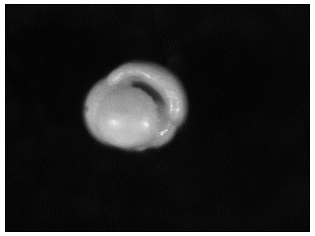
31
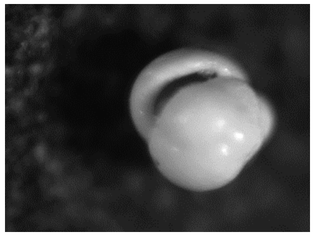
32
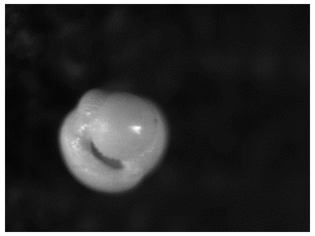
33
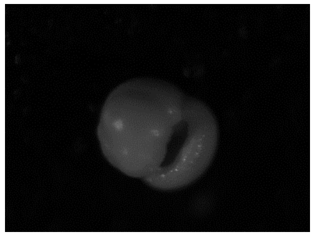
34
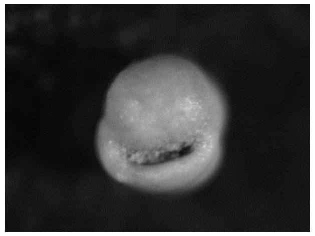
35
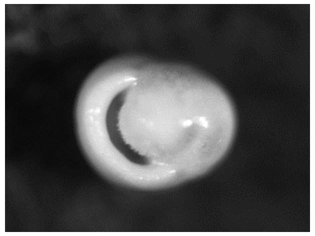
36
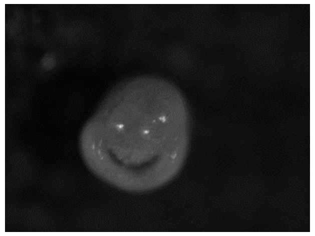
37
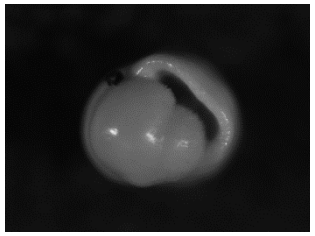
38
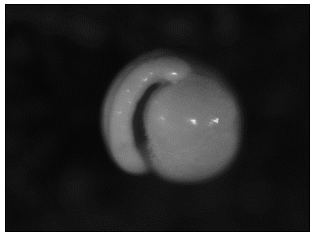
39
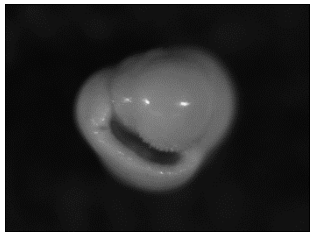
40
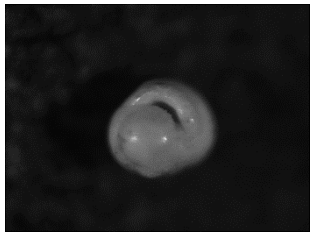
41
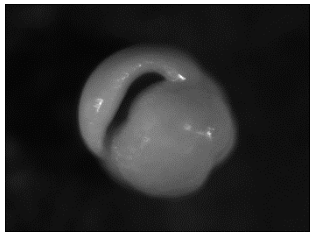
42
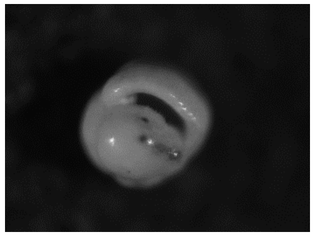
43
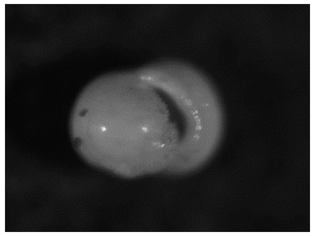
44
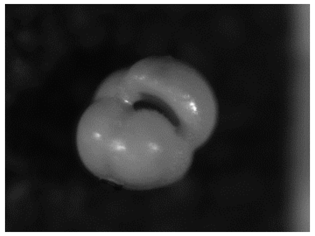
45
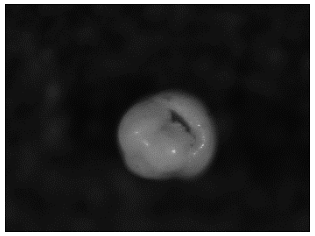
46
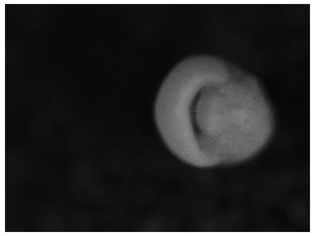
47
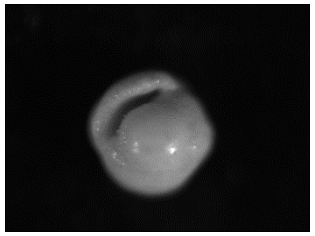
48
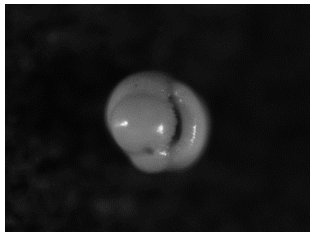
49
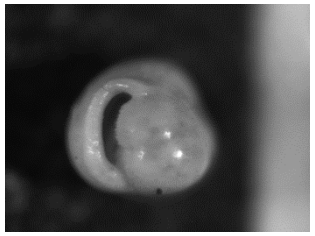
50

Sample U1483A/15H/6, 58-60 cm: dextral specimens

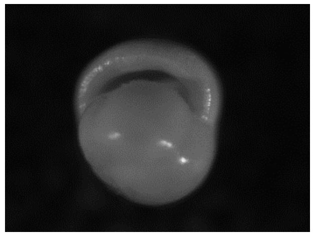
51
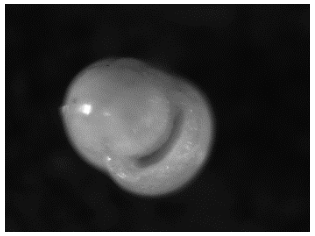
52
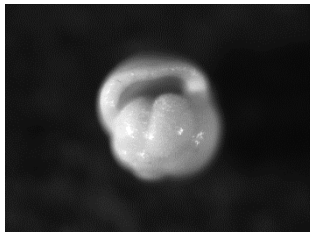
53
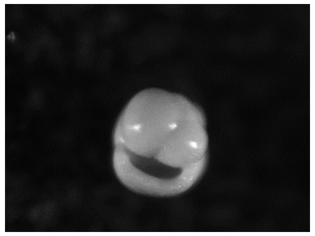
54
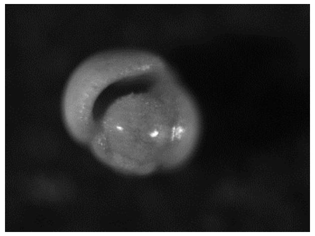
55
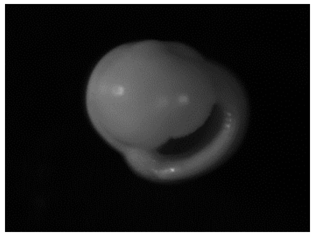
56
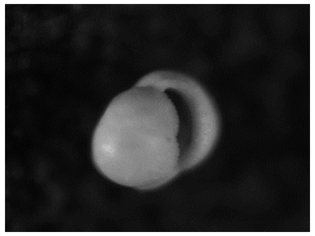
57
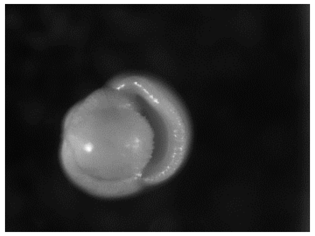
58
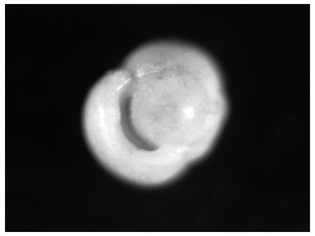
59
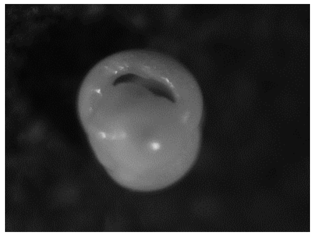
60
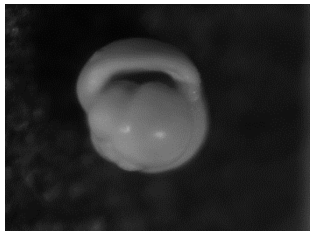
61
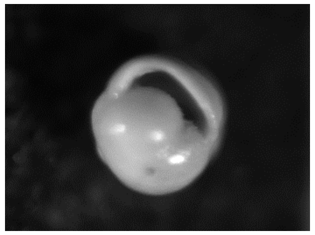
62
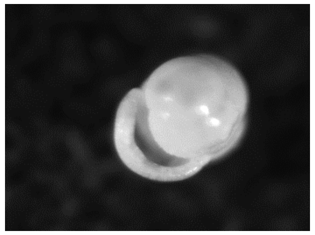
63
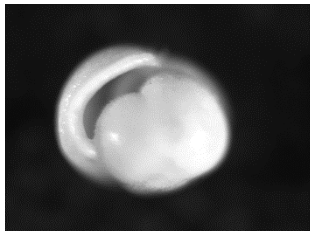
64
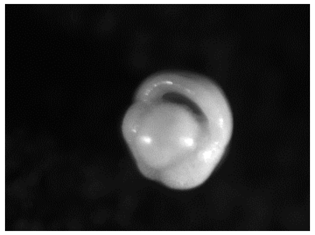
65
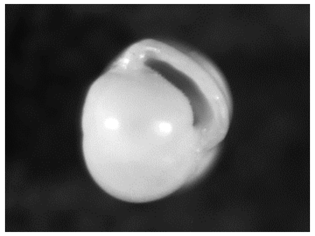
66
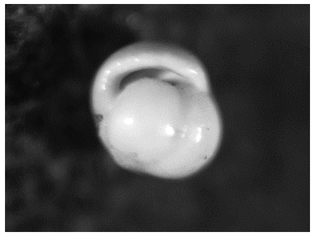
67
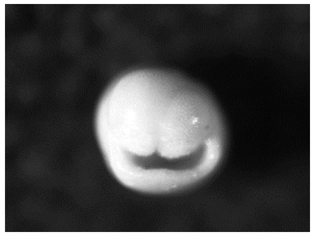
68
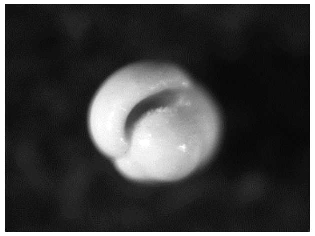
69
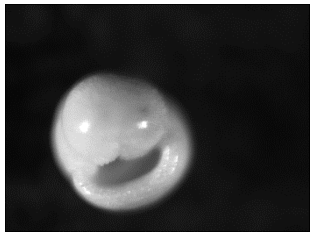
70
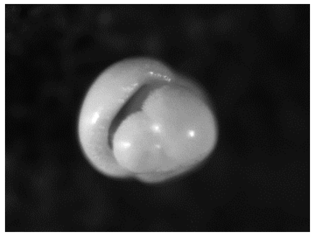
71
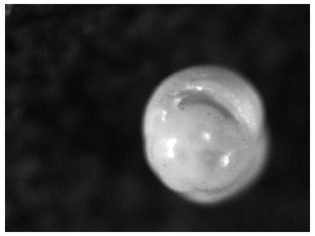
72
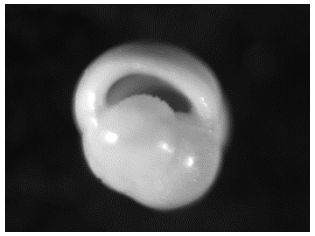
73
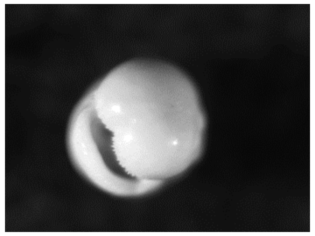
74
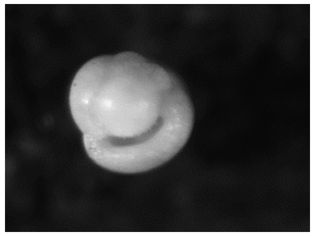
75

Sample U1483A/15H/6, 58-60 cm: dextral specimens

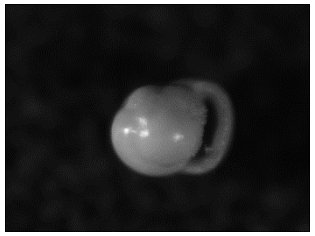
76
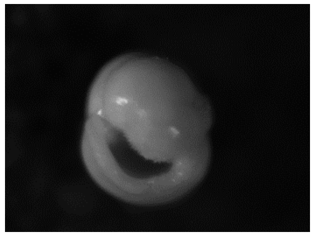
77
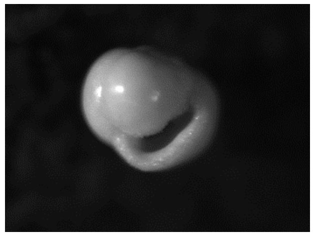
78
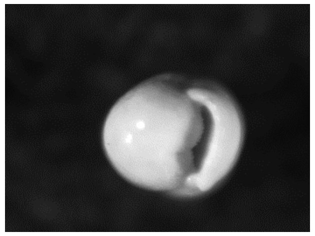
79
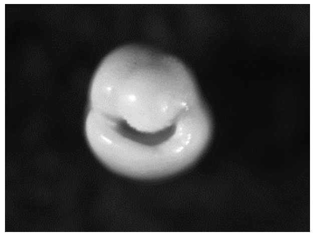
80
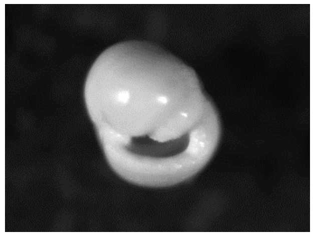
81
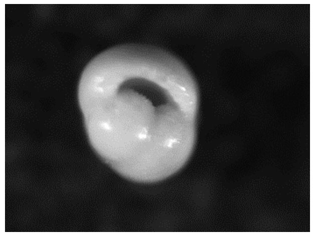
82
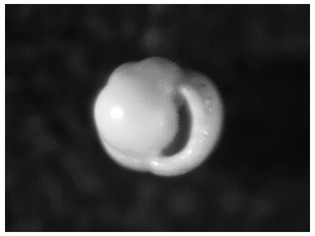
83
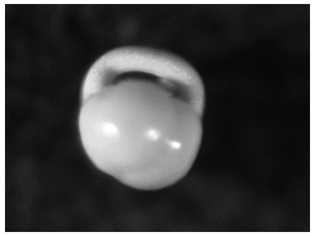
84
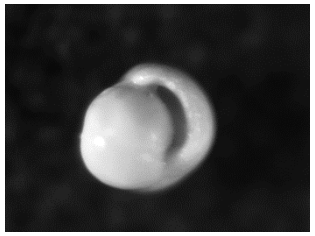
85
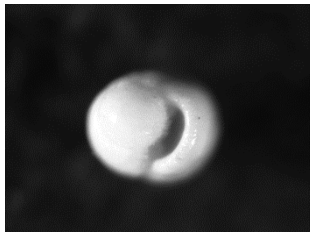
86
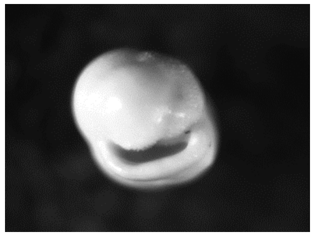
87
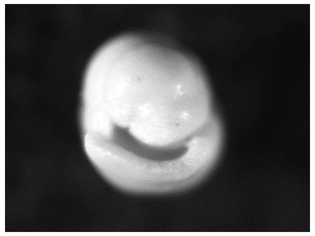
88
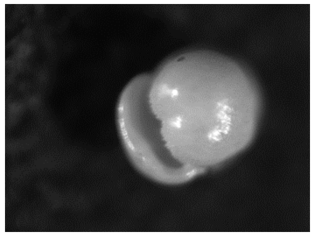
89
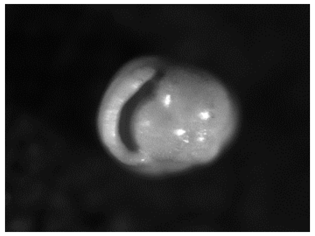
90
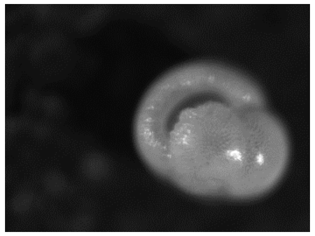
91
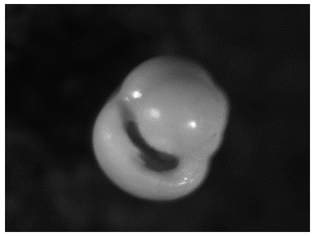
92
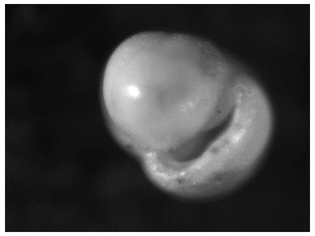
93
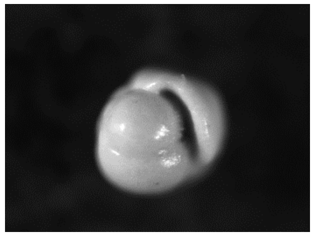
94
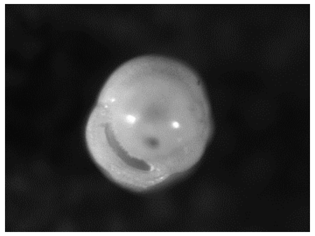
95
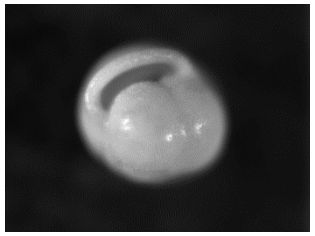
96
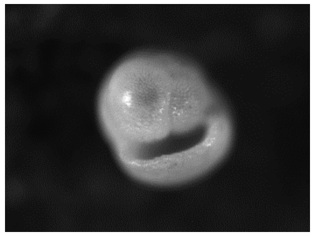
97
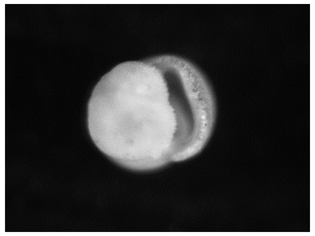
98
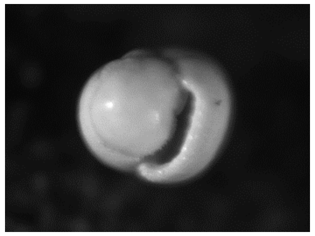
99
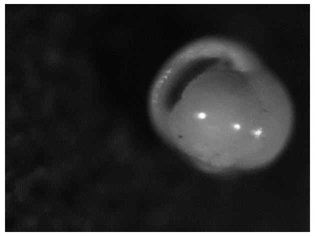
100

Sample U1483A/17H/4, 57-59 cm: sinistral specimens

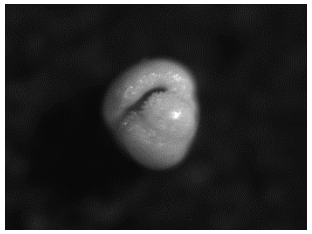
01

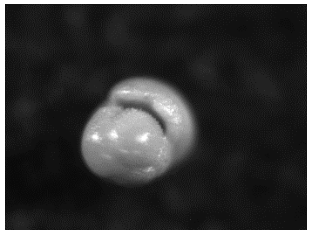
02

deleted
= *G. inflata*
03

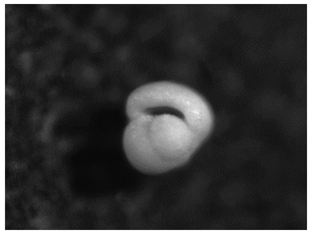
04

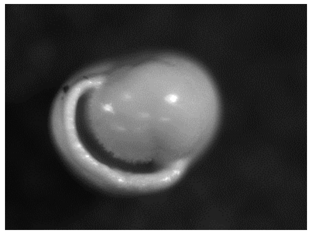
05

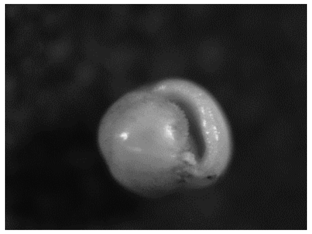
06

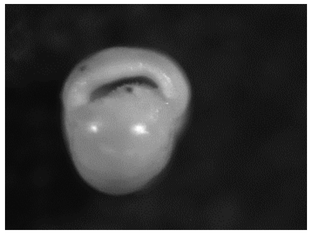
07

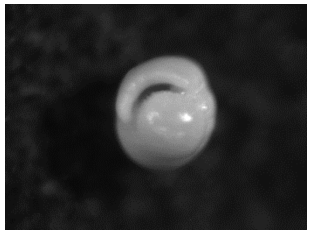
08

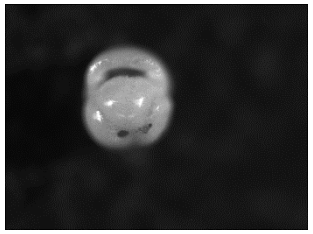
09

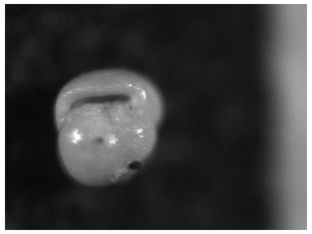
10

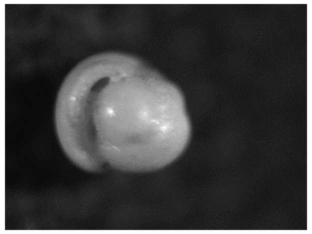
11

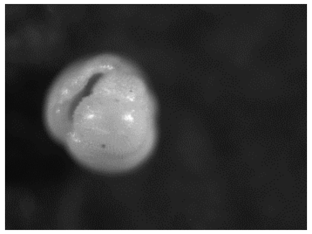
12

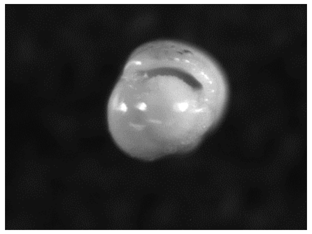
13

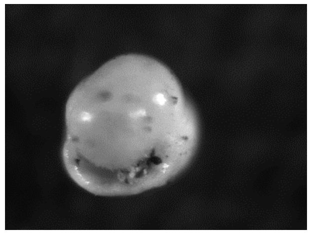
14

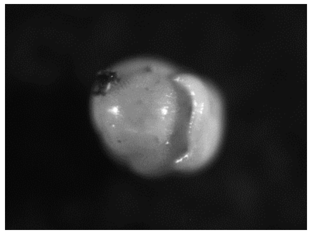
15

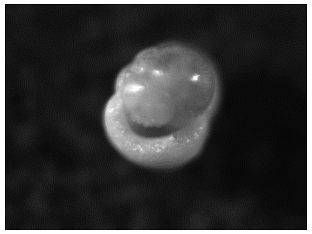
16

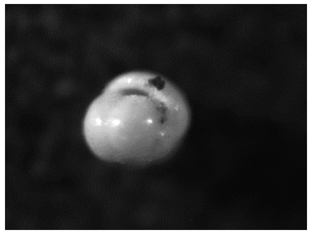
17

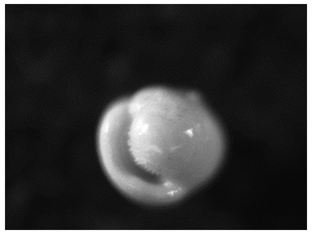
18

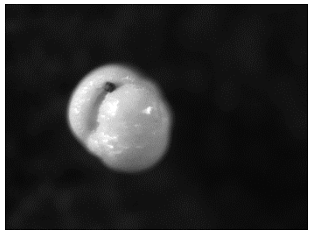
19

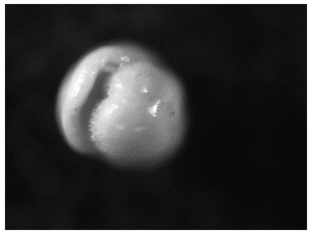
20

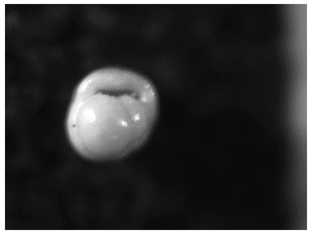
21

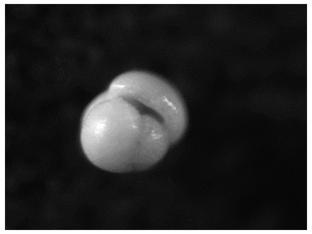
22

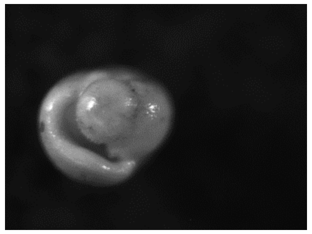
23

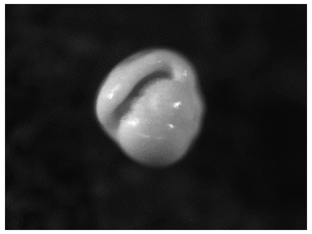
24

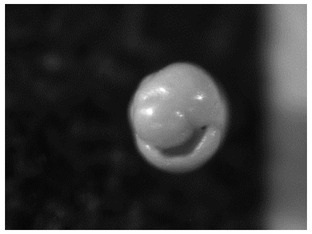
25

Sample U1483A/17H/4, 57-59 cm: sinistral specimens

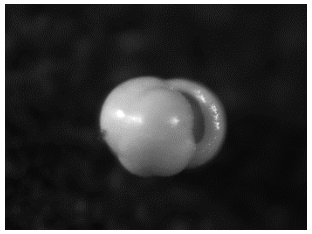
26

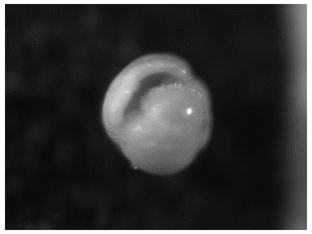
27

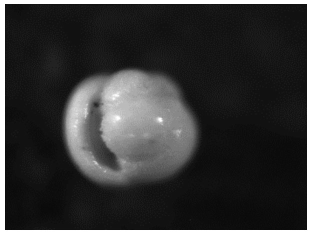
28

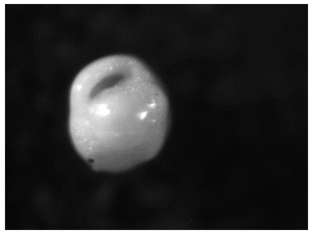
29

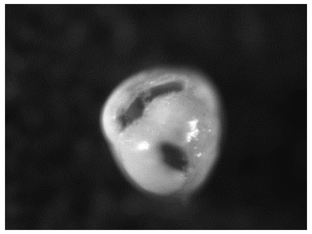
30

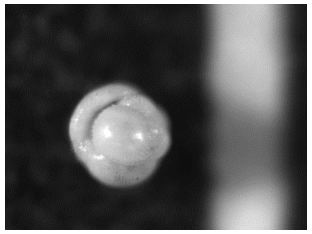
31

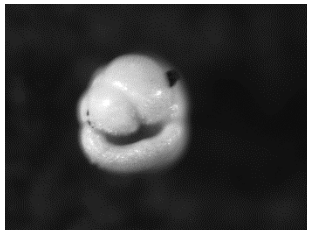
32

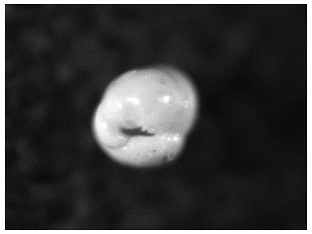
33

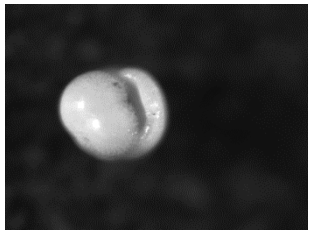
34

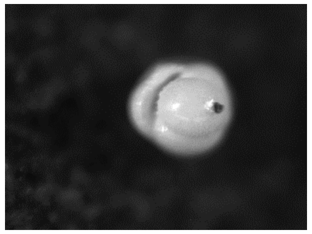
35

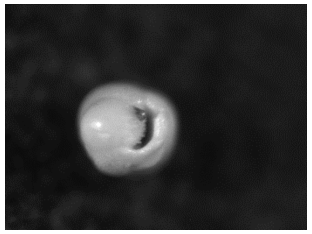
36

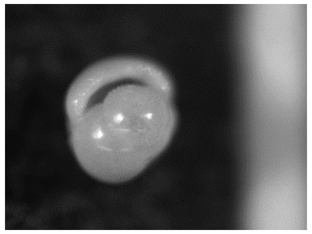
37

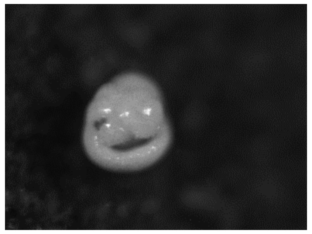
38

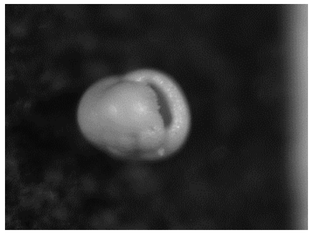
39

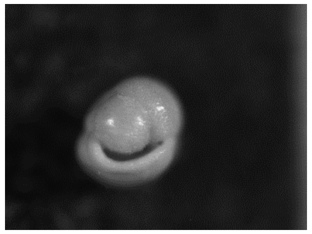
40

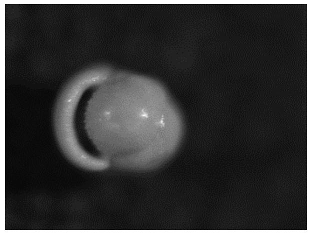
41

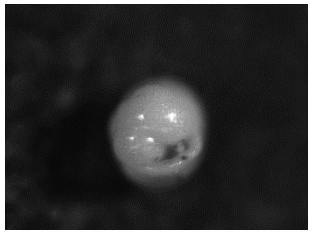
42

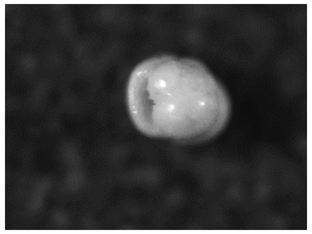
43

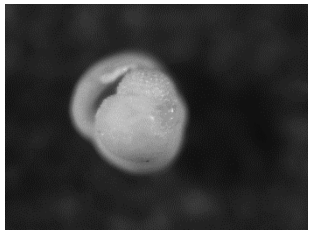
44

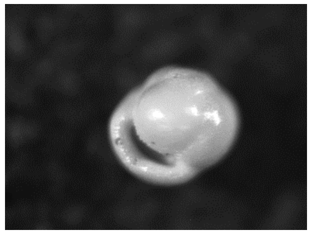
45

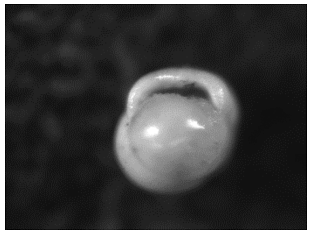
46

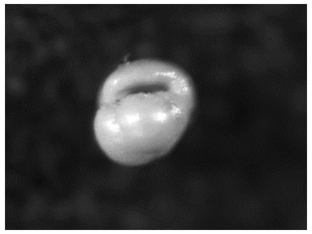
47

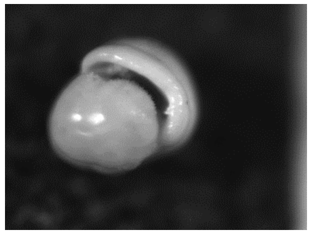
48

deleted
= *G. inflata*
49

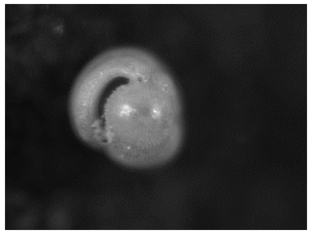
50

Sample U1483A/17H/4, 57-59 cm: sinistral specimens

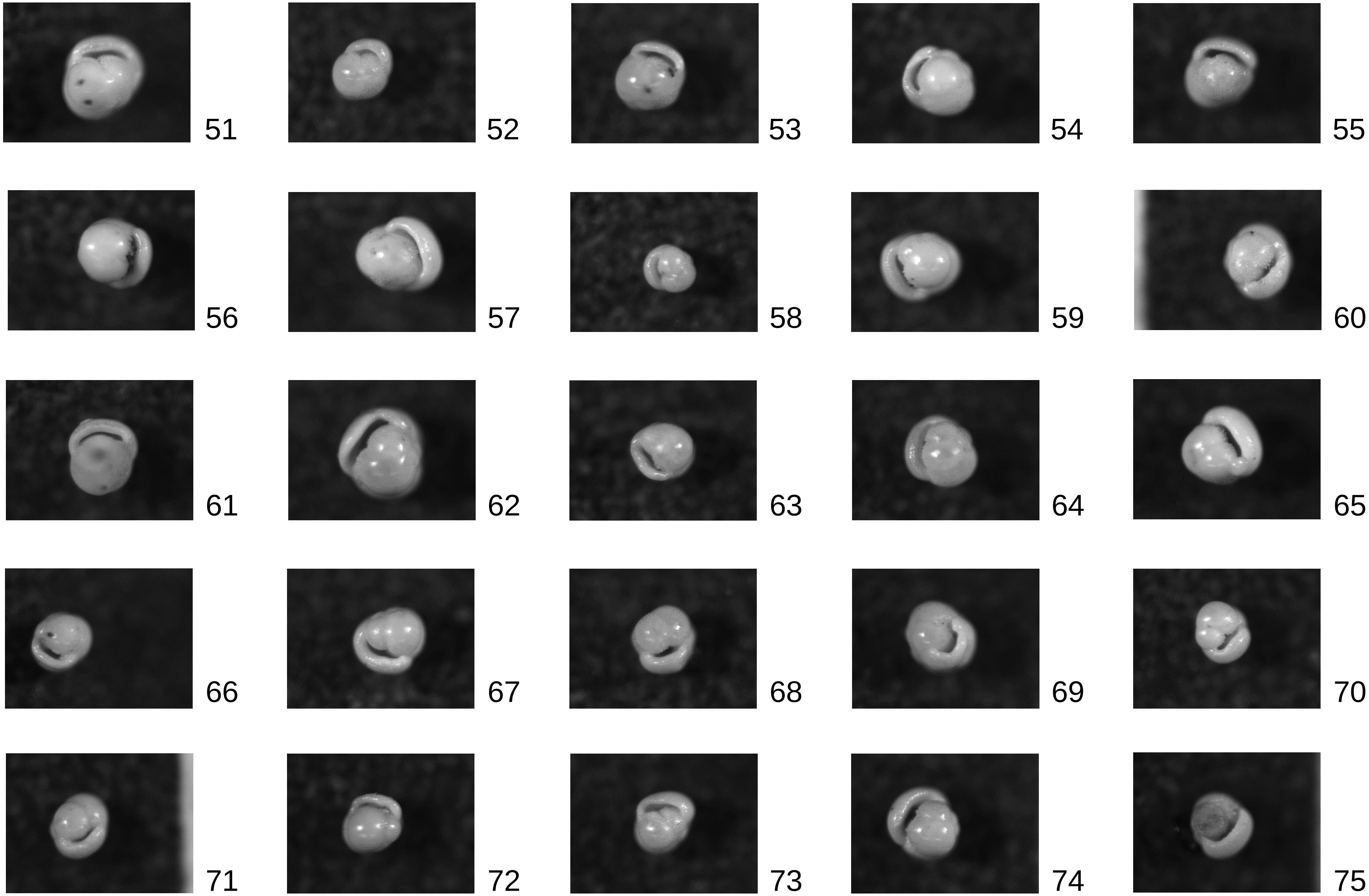

Sample U1483A/17H/4, 57-59 cm: sinistral specimens

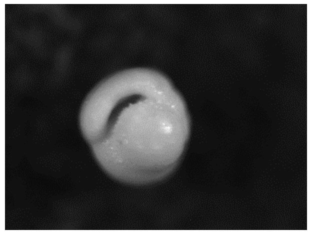
76

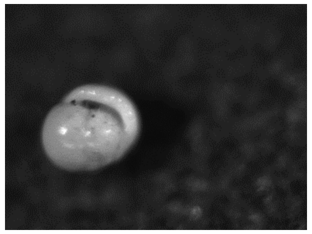
77

Sample U1483A/17H/4, 57-59 cm: dextral specimens

deleted
= *G. inflata*
78

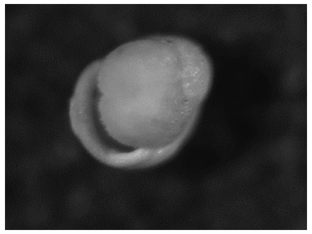
79

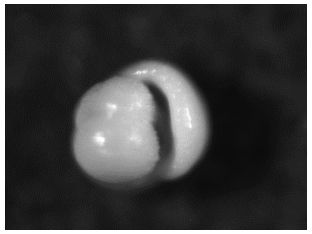
80

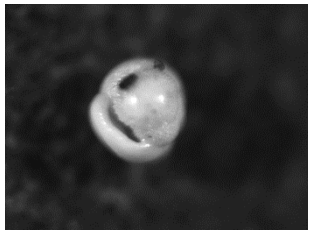
81

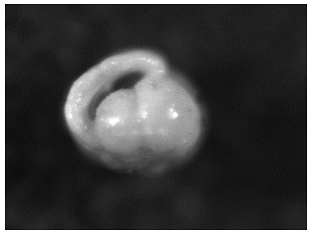
82

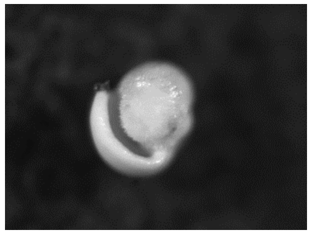
83

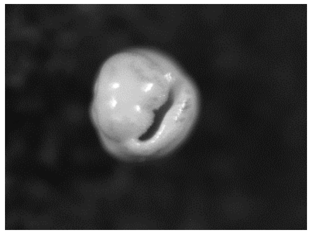
84

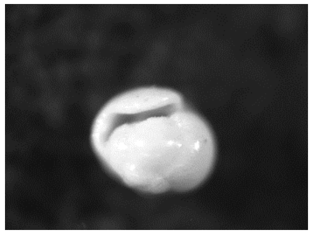
85

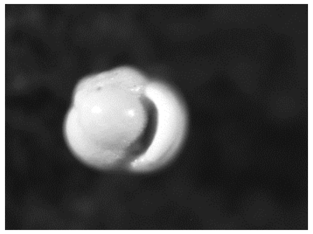
86

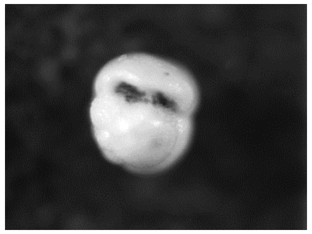
87

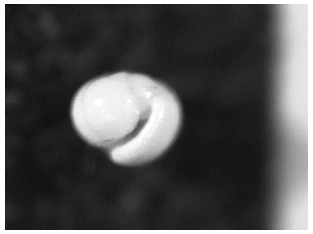
88

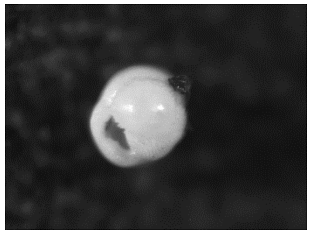
89

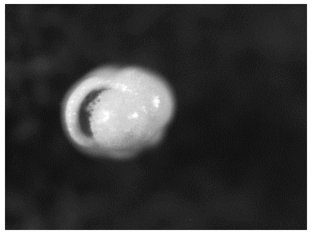
90

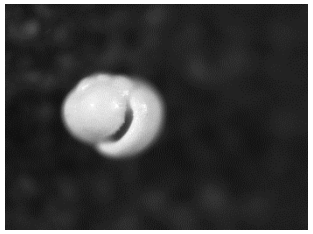
91

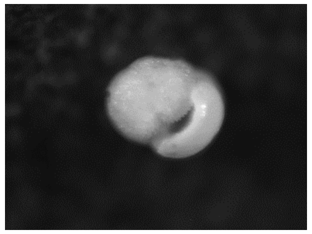
92

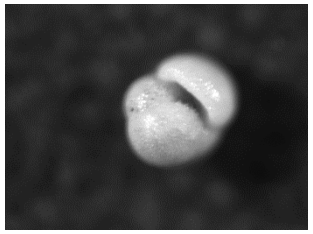
93

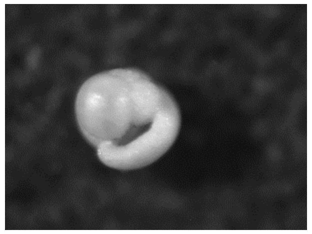
94

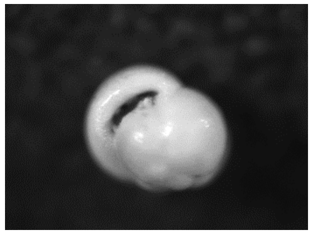
95
